# Epistatic hotspots organize antibody fitness landscape and boost evolvability

**DOI:** 10.1073/pnas.2413884122

**Published:** 2025-01-08

**Authors:** Steven Schulz, Timothy J. C. Tan, Nicholas C. Wu, Shenshen Wang

**Affiliations:** ^a^Department of Physics and Astronomy, University of California, Los Angeles, CA 90095; ^b^Center for Biophysics and Quantitative Biology, University of Illinois at Urbana-Champaign, Urbana, IL 61801; ^c^Department of Biochemistry, University of Illinois at Urbana-Champaign, Urbana, IL 61801; ^d^Carl R. Woese Institute for Genomic Biology, University of Illinois at Urbana-Champaign, Urbana, IL 61801

**Keywords:** combinatorial mutagenesis, sequence–function map, epistasis, heterogeneity, evolvability

## Abstract

The course of evolution is strongly shaped by interaction between mutations. Such epistasis can yield rugged sequence–function maps and constrain the availability of adaptive paths. While theoretical intuition is often built on global statistics of large, homogeneous model landscapes, mutagenesis measurements necessarily probe a limited neighborhood of a reference genotype. It is unclear to what extent local topography of a real epistatic landscape represents its global shape. Here, we demonstrate that epistatic landscapes can be heterogeneously rugged and this heterogeneity may render biomolecules more evolvable. By characterizing a multipeaked fitness landscape of a SARS-CoV-2 antibody mutant library, we show that heterogeneous ruggedness arises from sparse epistatic hotspots, whose mutation impacts the fitness effect of numerous sequence sites. Surprisingly, mutating an epistatic hotspot may enhance, rather than reduce, the accessibility of the fittest genotype, while increasing the overall ruggedness. Further, migratory constraints in real space alleviate mutational constraints in sequence space, which not only diversify direct paths taken but may also turn a road-blocking fitness peak into a stepping stone leading toward the global optimum. Our results suggest that a hierarchy of epistatic hotspots may organize the fitness landscape in such a way that path-orienting ruggedness confers global smoothness.

## Significance

Epistatic interactions between mutations are thought to constrain evolution since they make an adaptive landscape in sequence space very rugged. However, an increasing variety of empirical fitness landscapes appear to be both strongly rugged and highly navigable. That is, increased ruggedness tends not to reduce, and may even enhance, the evolutionary accessibility of the fittest genotype. By mapping a combinatorially complete antibody folding-stability landscape, we find that mutating an epistatic hotspot—an outgoing hub of epistatic interactions—generates heterogeneous ruggedness, which “funnels” the landscape toward its global optimum. Our results suggest that sparse hierarchical epistatic hotspots may render high-dimensional solution spaces easy to navigate, potentially underlying biomolecular evolvability and cell-fate plasticity alike.

A single mutation can modify the collective state of a biological system, such as the preferred structure of a protein molecule (1), which in turn alters the effect of subsequent mutations (2). Such nonadditivity, or epistasis, can strongly shape the course of evolution by making the fitness landscape (i.e., sequence–function map) highly rugged, with a multitude of distinct adaptive peaks. Understanding the structure of this map is key to predicting and steering evolution, because the shape of the fitness landscape carries information about viable paths and predictability of evolution, relative importance of genetic drift and selection, likelihood of convergent evolution, and the best achievable optimization, among others (see ref. 3 for a concise review).

Rapid advances in high-throughput methods now allow hundreds of thousands of genotype–phenotype pairs to be assayed in a single experiment (4). Still, these deep mutational scans (DMS) only probe a limited neighborhood of a particular parental sequence. In addition, mutational targets are chosen ad hoc; empirical fitness landscapes typically comprise individually identified beneficial mutations or those constituting an observed adaptive path. As a result, landscapes thus obtained may or may not represent the global shape. On the other hand, theoretical intuition often comes from model landscapes whose level of ruggedness is treated as a “bulk” property based on global statistics, assuming individual genetic bases or amino acids to be of similar epistatic importance.

Recent studies of empirical fitness landscapes challenge our understanding of evolutionary constraints, revealing inadequacy of existing landscape models: first, high-order genetic interactions introduce additional functional constraints and yet make evolution less predictable, by causing a delay of commitment to a genotypic fate (5). Second, high ruggedness needs not to lower the accessibility of the adapted states (6, 7). Third, epistatic involvement may vary strongly along the sequence of functional proteins (8–10). Are these behaviors related? Which features are generic across systems and functions? How does unequal epistatic involvement imprint landscape topography and impact navigation?

Here, we measure and characterize a multipeak fitness landscape of a SARS-CoV-2 antibody mutant library selected for folding stability, and reveal how the structure of landscape ruggedness influences adaptive paths and outcomes. To detect epistatic inequality, we infer models of specific and global epistasis, which are found to consistently pick up epistatically important sites. To probe heterogeneity of ruggedness, we analyze sublandscapes of varying size and location. Combinatorial completeness of the map allows us to uncover via simulations the role of higher-order epistasis in shaping the ruggedness–accessibility relationship. We finally explore how real-space migratory constraints affect sequence-space exploration, with or without epistatic inequality.

We find that real fitness landscapes can be heterogeneously rugged and that such heterogeneity may arise from the presence of sparse epistatic hotspots, whose mutation impacts the fitness effect of numerous sites. Counterintuitively, evolutionary constraints resulting from hotspot mutation act to promote, rather than impede, the access to the fittest state. In fact, the induced ruggedness appears to reorient the adaptive paths toward more productive directions, using suboptimal peaks as guideposts. Furthermore, spatial structure may turn an otherwise road-blocking peak into a stepping stone, significantly enhancing the success rate of enriching the fittest genotype. In contrast, model landscapes with homogeneous ruggedness show limited success—path-orienting features are generally lacking and populations get repeatedly trapped at suboptimal peaks. Our results suggest that heterogeneity among evolutionary degrees of freedom, induced by a hierarchy of epistatic involvement, can organize the fitness landscape and boost evolvability: not only that many starting sequences permit viable paths to the global optimum but that starting from the antibody germline, a majority of populations can navigate effectively. Such global “funneling” of an evolutionary fitness landscape via few epistatic hotspots may well be a target for antibody evolution that potentiates future evolution.

## Epistatic Hotspots Shape the Sequence–Function Map

### Mapping the Antibody Stability Landscape.

**Fig. 1.**
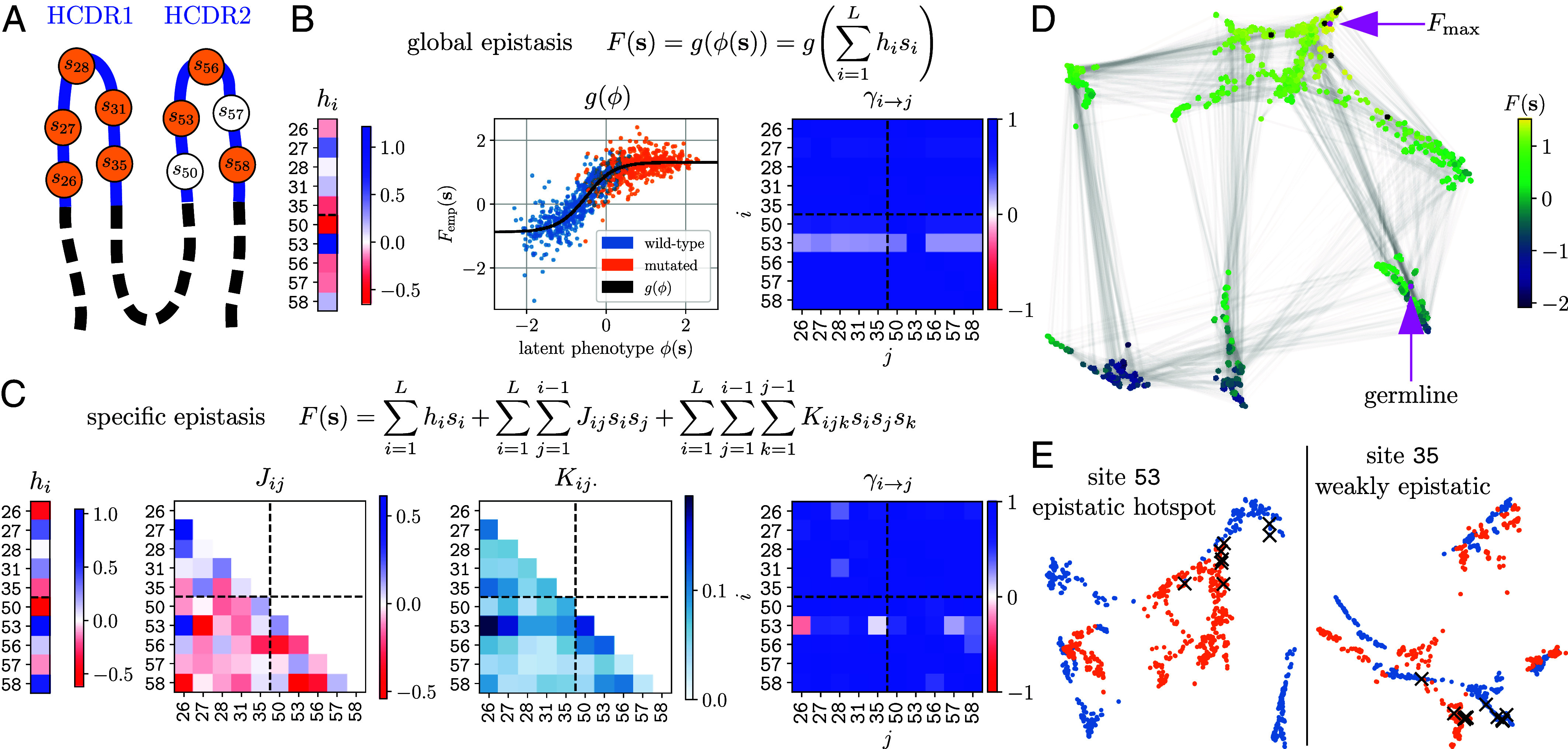
Inferring local and global epistasis in an antibody fitness landscape: topographical impact of an epistatic hotspot. (*A*) Sequence space is defined by L=10 frequently mutated residues across HCDR1 and HCDR2 (5 sites each) of SARS-CoV-2-specific antibody COV107-23. Each residue i is endowed with a spin variable $s_{i}$ that denotes the wild-type ($s_{i}=0$) or mutated ($s_{i}=1$) state. Orange sites are mutated in the global fitness maximum of the specific epistasis model (*C*). (*B* and *C*) Fitness landscape models F(s) fitted to the enrichment data of all $2^{L}$ possible sequences, namely a global epistasis model (*B*) and an Ising-type specific epistasis model (*C*). For global epistasis, the latent phenotype $\phi =\sum_{i}h_{i}s_{i}$ (colored strip of $h_{i}$) and the global nonlinearity g(ϕ) (black curve) are inferred simultaneously. Sequences (points) are colored according to the state of site i=53. For specific epistasis, inferred additive and epistatic coefficients, $h_{i}$, $J_{\mathrm{ij}}$, and $K_{ij\cdot }=\frac{1}{L-2}\sum_{k\neq i,j}\left|K_{\mathrm{ijk}}\right|$, are shown. Matrices of directed epistatic effects $\gamma_{i\to j}$ indicate how strongly mutation i alters the fitness effect of mutation j. (*D*) 2D force-directed network layout of the landscape. Each point represents one of $2^{L}$ sequences, colored according to its fitness F(s). Intracluster sequences have similar fitness, while intercluster gaps indicate fitness jumps. Pairs of mutational neighbors are connected by gray lines. Black dots mark local fitness optima in the specific epistasis model (*C*). Arrows point to the germline genotype and the global fitness maximum ($F_{\max}$). (*E*) Superposition of 9-site sublandscapes defined by holding the indicated site in its wild-type (blue) or mutated (orange) state. Shown are sublandscapes in the force-directed embedding with the epistatic hotspot (i=53) and a weakly epistatic site (i=35) being respectively held constant in the genetic background. Crosses mark fitness optima in either sublandscape.

To dissect the functional organization of biologically realistic fitness landscapes, we map the stability landscape of a SARS-CoV-2-specific antibody, COV107-23, that targets the receptor binding domain of the viral spike protein and is encoded by IGHV3-53/3-66 germline genes. To define the part of sequence space likely explored by antibody repertoires in the course of independent immune responses, we chose 12 most frequent somatic hypermutations (SHMs) retained in recovered COVID-19 patients (*SI Appendix*, section A1 and Fig. S1). These SHMs distribute over 10 sequence sites that span two variable loops (HCDR1 and HCDR2) of the antibody heavy chain (Fig. 1*A*) and play a variety of structural roles (*SI Appendix*, Fig. S2 and Table S1).

We built a combinatorially complete plasmid library containing all combinations of the 10 mutations (treating the three biochemically similar alternative amino acids at site 27 as the same mutated state, hence a total of $2^{10}=1,024$ variants). We transformed this plasmid library into a yeast strain and constructed a yeast display library in which each yeast cell expresses a single antibody variant corresponding to the plasmid in that cell. We then subjected this antibody variant library to selection for thermodynamic stability, using yeast surface expression as a proxy for stability. Since antibody variants that fold stably express on the cell surface with a fluorescent tag, we sorted cells by fluorescence. As a result of sorting, antibody-expressing yeast cells are enriched in frequency approximately in proportion to antibody stability. We sequenced both the unsorted and sorted yeast display libraries and quantified antibody fitness by logarithmic enrichment of sequence reads due to selection (see details in *SI Appendix*, sections A2–A5).

### Models of Epistatic Fitness Landscape.

Epistatic interactions can arise at any step in the mapping from genotype to phenotype and fitness, reflecting different origins and organization levels of functional coupling among constituents of a system. It is thus not a priori clear whether the genotype–fitness map of a particular system is better described by a model comprising specific interactions (e.g., physical contacts between protein residues) or would take the form of a global nonlinearity distorting an unobserved additive trait (e.g., protein stability as a function of folding free energy). We thus examine both (*SI Appendix*, section B1).

#### Specific epistasis model.

Considering that each sequence in the antibody variant library contains binary sites that are either wild-type (WT) or mutated, we express the map from genotype s to fitness F as a sum of combinations of biallelic loci at increasing orders:

[1] $$\begin{array}{c} F\left(s\right)=\sum_{i=1}^{L}h_{i}s_{i}+\sum_{i=1}^{L}\sum_{j=1}^{i-1}J_{\mathrm{ij}}s_{i}s_{j}+\sum_{i=1}^{L}\sum_{j=1}^{i-1}\sum_{k=1}^{j-1}K_{\mathrm{ijk}}s_{i}s_{j}s_{k}. \end{array}$$

We define that $s_{i}=1$ if the amino acid at site i is mutated and $s_{i}=0$ otherwise, with i=1,2,⋯,L running over the sequence length (L=10). The germline sequence has all sites being wild-type and thus has zero fitness. The local fields $\left\{h_{i}\right\}$ represent additive effects of individual mutations, and the higher-order terms with strengths $\left\{J_{\mathrm{ij}}\right\}$ and $\left\{K_{\mathrm{ijk}}\right\}$ correspond to epistatic interactions of concurring mutations in pairs and triplets, respectively. Combinatorial completeness of our sequence-to-function map allows us to go beyond pairwise couplings typical of maximum-entropy models of specific epistasis (e.g., ref. 8).

#### Global epistasis model.

A global epistasis model (11) assumes that an additive latent phenotype transforms nonlinearly to the observed phenotype, or fitness:

[2] $$\begin{array}{c} \begin{array}{c} F\left(s\right)=g\;\left(\sum_{i=1}^{L}h_{i}s_{i}\right). \end{array} \end{array}$$

Here, $\left\{h_{i}\right\}$ denote additive effects on the underlying phenotype and the transformation function g(·) specifies the shape of the global nonlinearity.

### Inference and Validation.

Measurement noise is primarily caused by finite sampling in the sorting step. Although the empirical fitness landscape based on log-enrichment of antibody expression shows strong correlations between duplicate experiments ($R^{2}=0.86$, *SI Appendix*, Fig. S3 *B*, *Upper Left*), the remaining variance could incur overfitting and introduce spurious ruggedness. To ensure a reliable fitness landscape model, we used two distinct methods to infer specific epistasis and paired each with a denoising procedure, namely, a maximum-likelihood method paired with cross-validation and Walsh–Hadamard transform followed by a band-pass filter (*SI Appendix*, section C). Two methods consistently show that specific epistasis up to the third order represents signal, while higher-order terms reflect noise (*SI Appendix*, Fig. S3*A*), supporting Eq. **1**. Indeed, after denoising, duplicate experiments yield virtually identical empirical landscapes ($R^{2}=0.98$, *SI Appendix*, Fig. S3 *B*, *Upper Right*). For global epistasis, we simultaneously inferred the additive effects and global nonlinearity using maximum likelihood (*SI Appendix*, section C).

### Interpretation of Inferred Models.

This network of antibody variants and their associated enrichment levels produce a rugged fitness landscape. Multiple genotypes constitute adaptive peaks (*SI Appendix*, Table S4), where no gain or loss of a mutation is able to increase fitness. Eight out of ten sites are mutated in the global fitness maximum, denoted by $F_{\max}$ hereafter. The inferred models of specific (Fig. 1*C*) and global (Fig. 1*B*) epistasis show consistent patterns of additive coefficients $\left\{h_{i}\right\}$ (vertical color strips): the sign of all terms matches, with single mutations on site 53 and site 50 being most beneficial and deleterious, respectively, on the germline background. While mutations G26E and Y58F—two other mutations required for reaching $F_{\max}$—have strong fitness effects based on the specific model, their contributions are small in the global model.

Notably, the specific model exhibits prevalent pairwise interactions with either sign, as well as widespread third-order interactions, especially significant for site 53 (the dark blue row in the $K_{ij\cdot }$ matrix, Fig. 1*C*). On the other hand, data trace the inferred global nonlinearity very closely (Fig. 1*B*). The fact that both specific and global epistasis models fit data well suggests a strong compression of parameter space and a potentially simple global shape of the evolutionary landscape. When data points are colored by the mutational state of each site, only site 53 clearly divides the variance across the nonlinear shape (Fig. 1*B*, segregation of the blue points with S53, lying along the slope, from the orange points with P53 that span the plateau), consistent with its strong positive effect on fitness (large $h_{i}$) and pervasive influence on epistatic interactions (*SI Appendix*, Figs. S9 *A* and *B*).

### Identification of an Epistatic Hotspot.

To quantify the epistatic importance of sequence sites, we use γ statistics (12) to compute the (directional) correlation of fitness effects of mutations: the matrix of $\gamma_{i\to j}$ quantifies to what degree the fitness effect of mutation j is altered, on average, by the presence of mutation i-pagination (*Materials and Methods*, *SI Appendix*, section E); mutating an epistatic hotspot would impact many other sites’ fitness effects. A lower γ value indicates a stronger local ruggedness.

Computing $\gamma_{i\to j}$ from both specific and global epistasis models consistently identifies site 53 as an epistatic hotspot—the S53P mutation influences nearly all other studied sites (Fig. 1 *B* and *C*). Inter-loop correlations are highly asymmetric (comparing off-diagonal blocks of the $\gamma_{i\to j}$ matrix): mutations in HCDR2 affect those in HCDR1, but not the reverse. Moreover, interloop and intraloop effects are similar in magnitude.

#### Structure and stability.

**Fig. 2.**
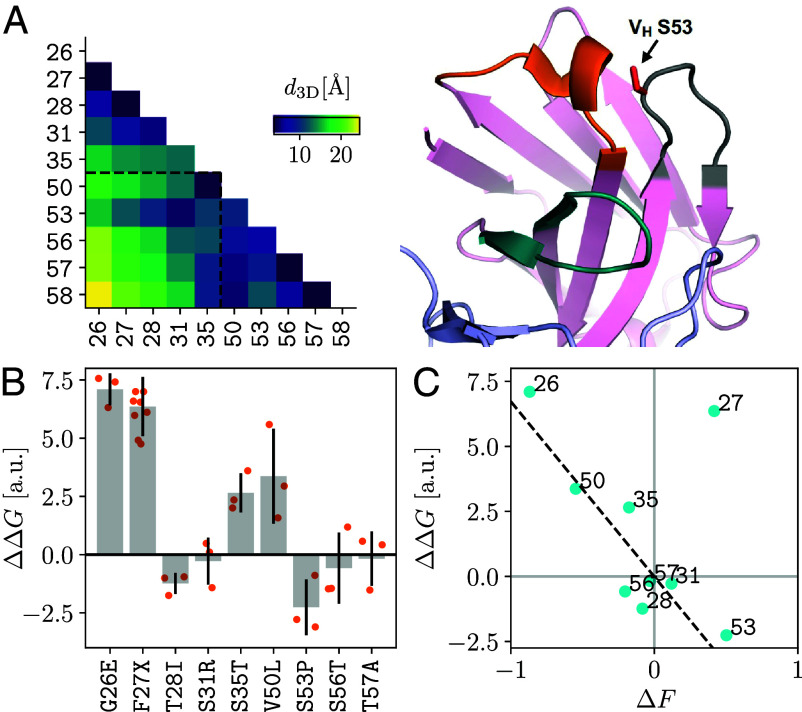
Structural interpretation of the epistatic hotspot. (*A*) *Left*: 3D distances (in Å) between pairs of $C_{\alpha }$ positions in the antibody fold. *Right*: Apo crystal structure of COV107-23 (PDB: 7LKA). Residue S53 in the variable region of the heavy chain is colored in red. Pink: heavy chain, light blue: light chain, orange: HCDR1, gray: HCDR2, teal: HCDR3. (*B*) Predicted ΔΔG for each mutation from simulations in Rosetta. Data points from independent replicates (orange dots) are shown, together with the mean (gray bars) ± SD (black lines). Site 27 has three biochemically similar mutations. (*C*) Replicate-averaged ΔΔG versus inferred fitness effect ΔF for each mutation in the F58 background (blue dots). A linear fit to all data points excluding site 27 crosses the origin (dashed line).

The strong heterogeneity and asymmetry in correlation patterns revealed by γ statistics motivate a closer look at the structural role of the hotspot. Structural analysis of the antibody shows that site 53 is physically closer to HCDR1 compared to any other HCDR2 residue studied here (Fig. 2*A*, 3D distances and crystal structure).

To evaluate the impact of a mutation on protein stability, we performed simulations using Rosetta (*SI Appendix*, section A6) to predict the difference in folding free energies, ΔΔG, between the wild-type COV107-23 and the indicated single mutant (Fig. 2*B*). Of note, COV107-23 has F58 instead of the germline version Y58. Clearly, mutation S53P causes the greatest decrease in folding free energy (most negative ΔΔG), suggesting that, among the point mutations studied here, mutating the hotspot most significantly stabilizes the antibody. This stabilizing effect, in line with the large fitness gain this mutation confers (Fig. 1 *B* and *C*), may facilitate acquisition of new functionalities such as binding to molecular targets (13).

To confirm that inferred fitness indeed reflects protein stability, we plot the predicted ΔΔG against the inferred ΔF for each mutation and found an approximately linear relationship (Fig. 2*C*), if site 27 were excluded. Overall, a destabilizing mutation (ΔΔG>0) tends to reduce fitness (ΔF<0) whereas stabilizing mutations (ΔΔG<0) increase fitness (ΔF>0). The outlier site 27 has a positive ΔΔG despite having a positive ΔF. This discrepancy could stem from epistatic coupling with residues outside the studied set. Another source of deviation could be the limited correlation between experimentally determined and computationally predicted ΔΔG values (14).

#### Low-dimensional map.

To visualize the impact of hotspot mutation on landscape topography, we embed a high-dimensional fitness surface in two dimensions using the force-directed layout (15) (Fig. 1 *D* and *E*, *Materials and Methods*, and *SI Appendix*, section D). In this representation, individual clusters collect genotypes of similar fitness, whereas wide divides between clusters indicate fitness gaps (Fig. 1*D*). Since clusters may coalesce as additional constraints are introduced, the fact that the state of the hotspot robustly sets clusters apart suggests its pivotal role in shaping landscape topography and inducing heterogeneity (*SI Appendix*, Fig. S4). To see this, we superpose the 2D maps of 9-site sublandscapes, with the background site being pinned in WT or mutated state (Fig. 1*E*, blue vs. orange dots). While mutating a weakly epistatic site only slightly modifies the embedding (*Right* panel), hotspot mutation not only reorganizes the entire map but also reduces fitness gaps (*Left* panel). These effects might enable efficient navigation.

## Results

### Mutation of an Epistatic Hotspot Increases Ruggedness Yet Enhances *F*_max_ Accessibility.

While in theory, a highly rugged landscape is hard to navigate, an increasing number of empirical studies using combinatorial mutagenesis found that ruggedness may not keep adapting populations from accessing high fitness peaks in high dimensions (5, 7). To probe the relation between ruggedness and accessibility in the antibody stability landscape, we evaluate the ruggedness of each 9-site sublandscape when the 10th site is held in WT or mutated state, and determine the accessibility of the global fitness optimum $F_{\max}$ in each pair of sublandscapes (*SI Appendix*, sections F and G).

**Fig. 3.**
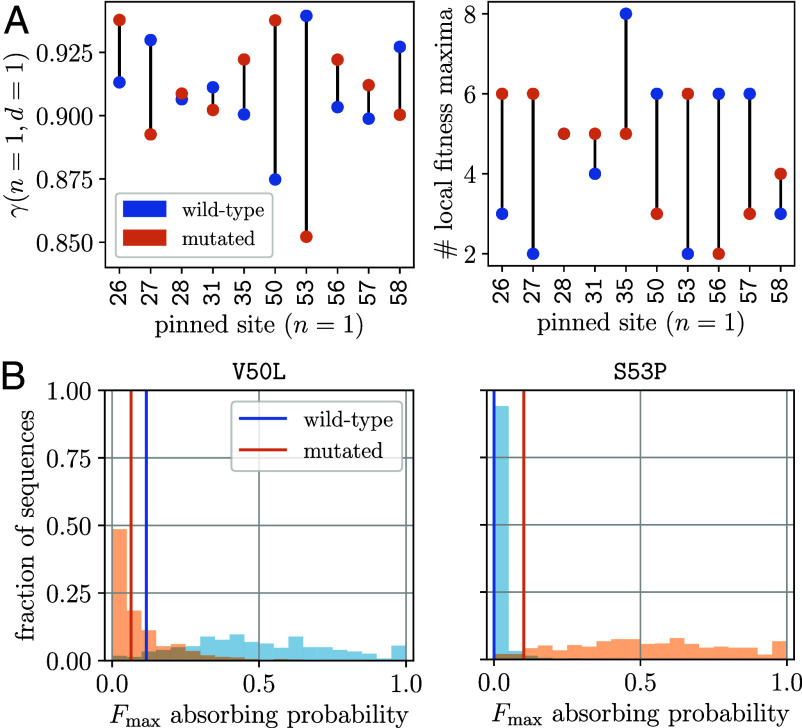
Hotspot mutation increases ruggedness of antibody landscape yet enhances $F_{\max}$ accessibility. (*A*) Ruggedness of 9-site sublandscapes with the remaining site being pinned (i.e., held constant), measured by the correlation of fitness effects of mutations γ(n,d) between genotypes d mutations apart with n pinned sites (*Left*) and the number of local fitness optima (*Right*). For each choice of the pinned site i, a comparison is made between the wild-type state ($s_{i}=0$, blue) and the mutated state ($s_{i}=1$, orange). Also see *SI Appendix*, Fig. S5 *A* and *B*. (*B*) Accessibility of the global fitness optimum $F_{\max}$ in 9-site sublandscapes when site 50 or site 53 is pinned, measured by the distribution of absorbing probabilities under Monte-Carlo evolutionary dynamics. The histograms show the fraction of starting genotypes that lead to a certain absorbing probability at the global optimum; the vertical lines indicate starting from the germline genotype. Results for other choices of the pinned site are shown in *SI Appendix*, Fig. S5*C*.

We find wide-ranging changes in sublandscape ruggedness upon single mutations (Fig. 3*A*, blue to orange). Site 53 and site 50 appear most impactful, consistent with their strong additive effect and broad epistatic involvement (Fig. 1*C*). Across all pairs, a rise/fall in ruggedness is associated with an increase/decrease in the number of fitness peaks (Fig. 3*A*, *Left* vs. *Right* panel), except when site 26 is pinned.

Interestingly, all mutations that individually create additional fitness peaks are retained in $F_{\max}$. In contrast, ruggedness-reducing mutations either are absent from $F_{\max}$ (sites 50 and 57) or occur late (sites 35 and 56) along successful trajectories connecting the germline to $F_{\max}$ (Fig. 5). This observation suggests that ruggedness may allow preferential access to the fittest genotype.

Accessibility of the global peak is determined as the probability that an adaptive walk with fitness-increasing steps discovers $F_{\max}$ before encountering any local peak (*Materials and Methods*; *SI Appendix*, section G). Necessary for a multipeaked fitness landscape, nonreciprocal sign epistasis (by which two deleterious mutations produce a fitness gain) is known to create fitness valleys and block direct paths (along which mutations add one by one). Yet, high-order epistasis can open indirect paths (via adaptive loss or conversion of mutations) that bypass fitness valleys (5, 16). Surprisingly, within the antibody stability landscape presented here, an increase in ruggedness—due to mutation of an epistatic hotspot—can lead to a greater accessibility of the most stable fold via direct paths.

As shown in Fig. 3*B*, *Right* panel, increased accessibility of $F_{\max}$ due to hotspot mutation, S53P, manifests in two ways: first, the likelihood of reaching $F_{\max}$ from the germline via an adaptive walk increases (vertical lines, blue to orange). Second, without this mutation, very few starting genotypes can find an accessible pathway (blue histogram, strongly peaked near zero absorbing probability). As the hotspot mutates, nearly all starting genotypes get access to $F_{\max}$, and around half of them do so more often than not (orange histogram). In contrast, a ruggedness-decreasing mutation, V50L, closes off pathways accessible under stronger ruggedness (Fig. 3*B*, *Left* panel). Combined with a large deleterious effect, this could explain the absence of V50L from $F_{\max}$.

Such positive correlations between ruggedness and accessibility are not expected from fitness landscapes with homogeneous statistical properties. They suggest, instead, that the presence of sparse loci of high epistatic importance can strongly shape landscape topography such that local behaviors differ substantially from global patterns. In other words, a realistic epistatic landscape may well be heterogeneously rugged.

### Epistatic Hotspot Induces Heterogeneous Ruggedness.

Widely used models of rugged fitness landscapes (e.g. the NK model and the rough Mt. Fuji model) seldom account for variation in epistatic importance among sequence loci. If present, however, such variation may strongly influence the speed and predictability of evolution. Does an uneven degree of epistatic involvement translate to heterogeneous ruggedness across the fitness landscape? To probe heterogeneity, we determine whether, and how, ruggedness varies with the size and location of sublandscapes; these sublandscapes result from projecting the full landscape into (L−n)-dimensional sequence subspaces, that is, pinning n out of L sites in WT or mutated state (*SI Appendix*, section F). We compare our antibody landscape to NK landscapes, because the latter exhibit statistically homogeneous ruggedness at a tunable level (*SI Appendix*, section B2).

**Fig. 4.**
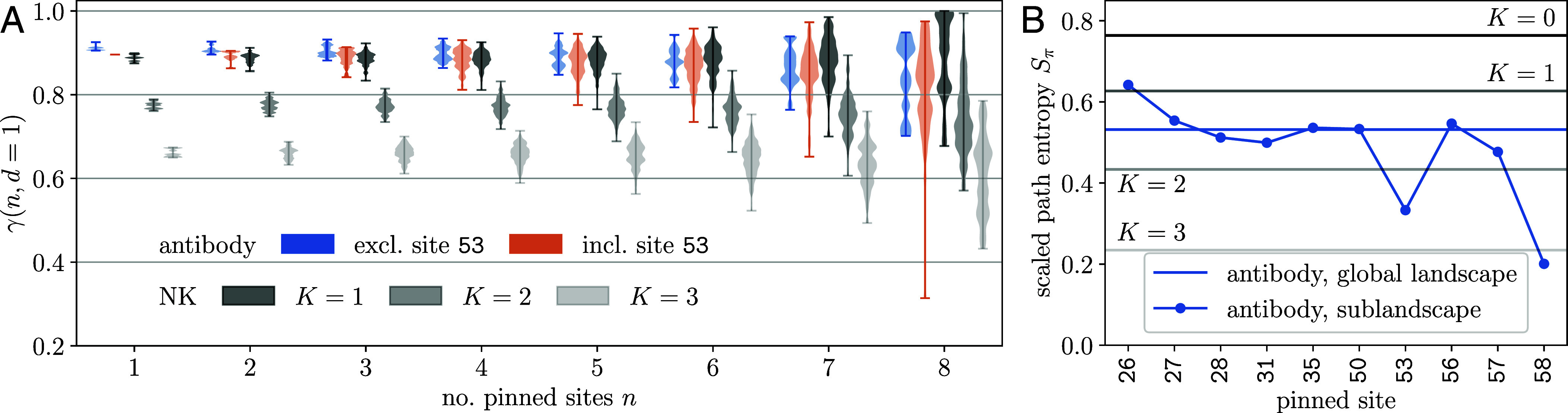
Epistatic hotspot induces heterogeneous ruggedness and restricts path diversity. (*A*) Strong variation of ruggedness with respect to the size and location of the antibody sublandscape. To quantify heterogeneity, we vary the number (n) and identity (in total Ln choices) of the pinned sites and compute the ruggedness of the resulting (L−n)-dimensional sublandscapes. Colored violins at each choice of n represents the range of ruggedness values due to varying locations, when the hotspot (site 53) is included in the pinned background (orange) or excluded from it (blue). The antibody landscape appears more rugged (low-lying γ values) when the hotspot is held in its mutated state. The corresponding distributions in the NK landscape are shown for comparison (gray violins). Note that the range of γ values is independent of the degree of ruggedness controlled by K, indicating statistical homogeneity. (*B*) Scaled path entropy $S_{\pi }$ measures the diversity of adaptive paths from the germline to the global optimum under Markov-chain Monte-Carlo evolutionary dynamics. Results are shown for the full antibody landscape (blue horizontal line) and 9-site sublandscapes (symbols). Hotspot mutation is strongly path-constraining. Gray lines show the expectations from NK landscapes with moderate ruggedness.

Fig. 4*A* presents the distribution of sublandscape ruggedness due to a varying location for both the antibody and the NK model. First, when the epistatic hotspot is pinned among the n sites in the background (orange violin), a stronger variation in ruggedness is observed for antibody sublandscapes compared to that in NK models (gray violins, lighter shade for larger K hence stronger ruggedness). By contrast, when the hotspot is excluded from the pinned background (blue violin), no such distinction is seen. Moreover, unlike the NK model that showed symmetric distributions, antibody sublandscapes exhibit skewed distributions—low-lying γ values correspond to pinning the hotspot in the mutated state. Both contrasting behaviors stem from epistatic inequivalence among loci that is present in the antibody landscape but not in homogeneous models. This divergence between data and model grows more pronounced in smaller sublandscapes (larger n) because it becomes more likely that the hotspot enters the background and shapes the sublandscapes, making them more rugged.

We next evaluate how the presence of an epistatic hotspot impacts path repeatability—a weak form of evolutionary predictability. We simulated an ensemble of adaptive paths connecting the germline to $F_{\max}$ using Monte Carlo dynamics and computed path entropy (Gibbs-Shannon entropy) from path weights (*Materials and Methods* and *SI Appendix*, section G).

Fig. 4*B* shows that path entropy varies considerably across 9-site sublandscapes (with the 10th site pinned to its state in $F_{\max}$, blue symbols). These variations correspond to a range of ruggedness spanning a multitude of K values in the NK model (gray lines). In particular, mutating site 53 or site 58 strongly restricts the *realized* path diversity, concentrating most weight to few direct paths and thus increasing the *retrospective* predictability. Yet, as a whole, the antibody landscape appears rather smooth (blue horizontal line); its path entropy is close to that of modestly rugged NK landscapes (with K=1–2).

Therefore, both landscape topography and path diversity suggest that this protein stability landscape behaves as if it were comprising NK sublandscapes at different K. That is, an adaptive landscape, shaped by a handful of epistatically impactful sequence loci, may exhibit a varying degree of ruggedness when observed at different locations and scales.

While natural proteins must be globally stable to fold, they often need to be locally unstable to function. Such localized instability typically arises from conflicting interactions, i.e., frustration (17–19), that allow specific movements around the native fold. We find it striking that the ability to fold promotes an efficient search of sequence space rather than hinder it (Fig. 3), and that it does so through concentrating mutational constraints to a small number of organizing centers (hotspots) from which epistatic interactions emanate. It is therefore intriguing to see whether the productive role of heterogeneous ruggedness links to localized frustration (20). Frustration analysis on wild-type and mutated versions of three sites (S53P, S35T, and F27X) in the heavy chain of COV107-23 reveals considerable variation in frustration levels among sites of different epistatic importance (*SI Appendix*, section A7). Specifically, hotspot mutation S53P increases local frustration (*SI Appendix*, Figs. S11 and S12) while rigidifying the HCDR2 loop and facilitating antibody binding to viral spike (*SI Appendix*, Fig. S13). On the contrary, mutating site 27 reduces frustration (*SI Appendix*, Figs. S11 and S12) while weakening antibody–spike interaction (*SI Appendix*, Fig. S13). This potential connection between heterogeneous ruggedness and localized frustration warrants future investigation.

### Spatial Structure Relaxes Mutational Constraints and Promotes Path Diversity.

**Fig. 5.**
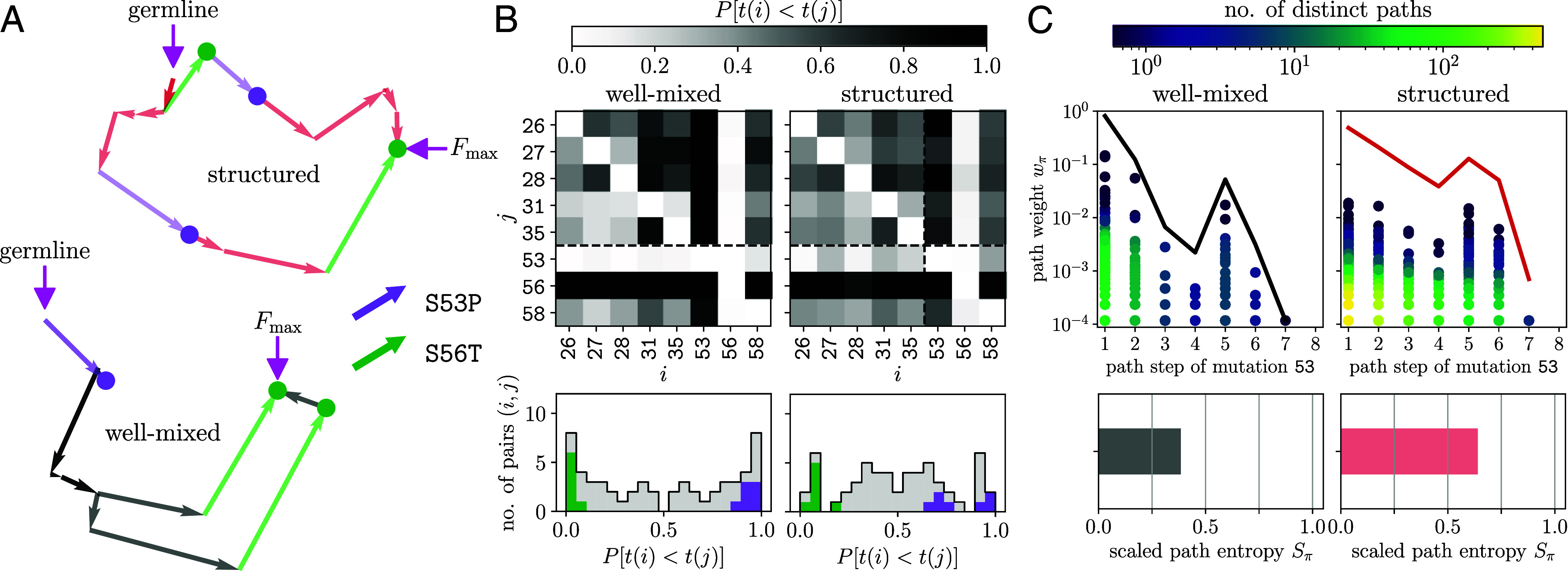
Spatial structure relaxes mutational constraints and diversifies direct paths taken. (*A*) Representative paths taken by an adapting population in the antibody landscape, evolving from the germline to the global fitness maximum $F_{\max}$ under Wright–Fisher dynamics. Consecutive arrows indicate mutational steps in the t-SNE layout of sequence space (with darker shade reflecting path overlap). In the well-mixed condition (black), mutation S53P (purple arrow) occurs ahead of any other mutation, while S56T (green arrow) occurs toward the end. In spatially structured populations (red), S53P may occur at later steps (lower path) and occasionally, S53P and S56T swap their order (upper path as an example). (*B*) Order constraints between pairs of mutations required to reach $F_{\max}$, shown by the matrix of probabilities P[t(i)<t(j)] that mutation i precedes mutation j (upper row) and the corresponding histogram of matrix entries (lower row), under well-mixed (*Left*) and structured (*Right*) conditions. Purple (green) bars highlight the entries corresponding to mutation at site i=53 (i=56). (*C*) Diversity and abundances of successful paths (reaching $F_{\max}$) depend on the path step at which hotspot mutation occurs. Successful paths are sorted by the path step of mutation 53. In each category, a realized path weight $w_{\pi }$ (dot) is colored by the number of unique paths taken by a fraction of $w_{\pi }$ evolving populations; the solid lines trace the cumulative path weight across the categories. In comparison with the well-mixed condition (*Left*), spatial structure diversifies viable paths (*Right*), yielding a larger overall path entropy (*Lower* panels).

Long-term adaptation is determined by the distribution of fitness effect of possible mutations and the stochastic processes that lead to fixation. As mutations arise and fix one by one along a direct path, an adapting population explores sublandscapes with varying ruggedness. From the results above, it seems paradoxical that hotspot mutation S53P increases the accessibility of $F_{\max}$ (Fig. 3*B*) while reducing the diversity of viable paths connecting the germline to $F_{\max}$ (Fig. 4*B*). With this paradox comes a hypothesis: a successful path might start in a relatively rugged landscape, where epistatic constraints incurred by hotspot mutation play a beneficial role in orienting the path toward productive directions. This intuition is backed by the observation that a posteriori approaches are unlikely to find much sign epistasis, especially at the later stage of adaptation, because realized mutations have collectively been tested by evolution. Successful paths found by in silico evolution following Wright–Fisher dynamics appear to support our proposal (Fig. 5 *A* and *B*): ruggedness-increasing mutations (esp. S53P) tend to precede ruggedness-reducing ones (e.g., S56T).

Fig. 5*A* displays examples of successful trajectories in a two-dimensional embedding (Materials & Methods; *SI Appendix*, section D); arrows along the path indicate mutational steps. We highlight the first occurrence of two mutations that are required to reach $F_{\max}$: S53P (purple arrow) increases ruggedness for subsequent adaptive steps, whereas S56T (green arrow) reduces sublandscape ruggedness. In most of the realized trajectories, S53P occurs within the first few steps, whereas S56T appears near the end (e.g., trajectories in the lower panel).

Fig. 5*B* presents the pairwise order of occurrence among the 8 required mutations along successful paths. We compute the probability that mutation i occurs ahead of mutation j, P[t(i)<t(j)], from a large ensemble of simulated populations obeying Wright–Fisher dynamics (*SI Appendix*, section G). In a well-mixed population, immediately beneficial mutations can rapidly sweep through. As a result, certain mutations occur in a strict order. As shown in the left column, S53P arises and fixes earlier than any other required mutation in essentially all cases (dark column) whereas S56T occurs later than any other mutation (light column). When collecting the matrix entries into a histogram of ordering probability (Fig. 5*B*, *Lower Left*), we observed a bimodal distribution that peaks at 1 and 0, where entries of site 53 (purple bars) and site 56 (green bars) reside, respectively.

Spatial structure in general slows the spreading of beneficial mutations, temporarily shielding genetic variation. A greater variation, in turn, allows for a broader search of the genotype space, giving time for beneficial combinations to arise and overtake the population. Guided by this intuition, we study how spatial structure affects the mutational order along successful paths and alters path diversity. We account for spatial structure in a minimal setting where migration only occurs between nodes within a neighborhood of size r on a ring lattice (*Materials and Methods* and *SI Appendix*, section G). Thus, a mutation can at most spread by r nodes in one generation.

In our antibody landscape, spatial structure is found to relax epistatic constraints: The distribution of ordering probability has a larger weight near 1/2, indicating similar chances of observing either order for most mutations (Fig. 5*B*, *Lower Right*). For the two extreme cases (S53P and S56T), the timing of occurrence now shows a wider spread and, occasionally, their order swaps (top path in Fig. 5*A*). Intuitively, one would expect that relaxation of mutational-order constraints implies an increase in path diversity. This is indeed the case in terms of the overall path entropy (Fig. 5*C*, *Lower* panels).

To see whether the timing of hotspot mutation would matter for success, we sort successful paths by the step at which hotspot mutation occurs and, in each class, we present the number of unique paths (color coded) associated with each realized path weight (dot); see Fig. 5*C*, *Upper* panels. In well-mixed populations, few high-weight paths where site 53 mutates in the first step dominate, indicating the importance of early hotspot mutation for reaching $F_{\max}$ within a given period of time. With spatial structure, different path classes contribute more evenly (red vs black outline). Moreover, a large variety of low-weight paths now open (yellow dots) in which S53P occurs within the first few steps, suggesting that the beneficial role of hotspot mutation in steering landscape navigation remains in effect.

### Heterogeneous Ruggedness Can Boost Evolvability.

Evolvability of proteins or cells refers to the adaptive potential to meet future challenges. For proteins, the ability to evolve a stable fold potentiates evolution of new functions; in the case of antibodies, this includes binding to a new variety of target antigens and improved binding affinity to previously encountered antigens. Thus, one proxy for antibody evolvability is the success rate at which the most stable fold (i.e., $F_{\max}$) is enriched within a limited amount of time. To illuminate how heterogeneous ruggedness influences evolvability, we study dynamics of landscape navigation by finite populations (following Wright–Fisher dynamics), where success rate is defined as the fraction of replicate populations in which $F_{\max}$, as opposed to any local optimum, first reaches an occupancy threshold (*Materials and Methods* and *SI Appendix*, section G). We expect that, by varying this threshold, the trend of change in success rate will distinguish the antibody landscape from homogeneous model landscapes.

**Fig. 6.**
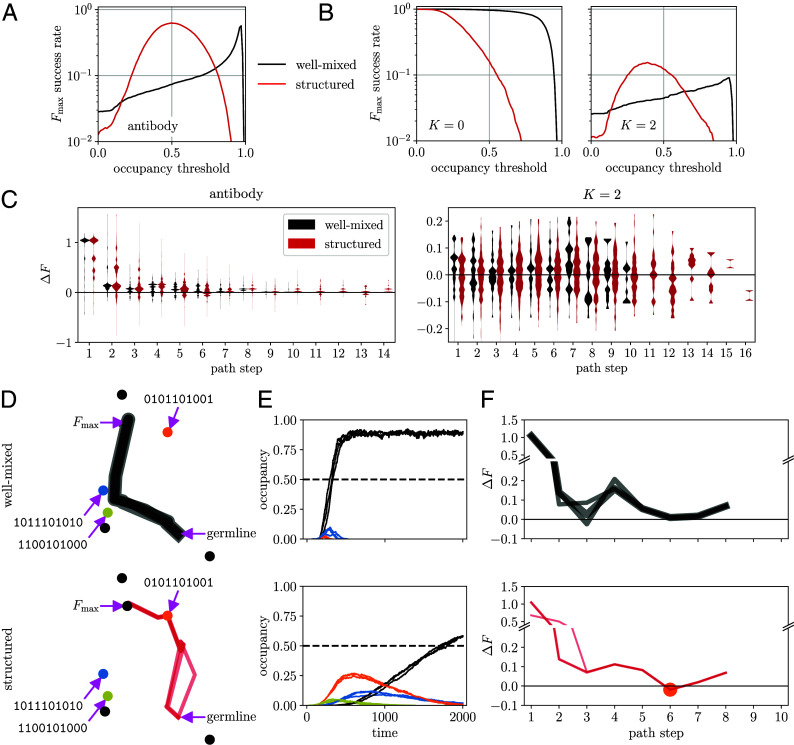
Heterogeneously rugged antibody landscape supports smooth adaptive paths and efficient navigation. (*A*) Success rate at which replicate populations evolving in the antibody landscape under Wright–Fisher dynamics first enrich the global optimum, as opposed to any local optimum, to a certain occupancy threshold (criterion of success). Spatial structure (red) enhances success at intermediate values of the occupancy threshold over the well-mixed condition (black). (*B*) Success rate as a function of occupancy threshold in smooth (K=0) and rugged (K=2) NK landscapes, without (black) and with (red) spatial structure. The curves for K=2 are an ensemble average over 50 independent realizations of the landscape, constrained to have the same number of fitness optima and the same mutational distance from the germline to $F_{\max}$ as the antibody landscape. (*C*) Distribution of fitness effects of mutations, ΔF, along successful paths collected from replicate populations evolving in the antibody (*Left*) and constrained NK (*Right*) landscapes, without (black) and with (red) spatial structure. (*D*–*F*) Characteristics of top-3 successful paths at occupancy threshold 0.5, without (upper row) and with (lower row) spatial structure. (*D*) Mutational paths in t-SNE representation. Line width is proportional to the path weight. Dots indicate 7 fitness optima. The orange dot represents the local optimum that is a road block bypassed by successful well-mixed populations but may serve as a stepping stone to structured populations taking a direct path. (*E*) Temporal occupancy of the fitness optima along the paths shown in (*D*). Each curve is an average over successful populations taking that path, with the same color codes as the fitness optima in (*D*). (*F*) Fitness effect of mutations out of each genotype along the paths shown in (*D*). The stepping-stone genotype is marked with an orange dot. Line width is proportional to the path weight.

Fig. 6 *A* and *B* present the success rate as a function of occupancy threshold in the absence and presence of spatial structure (black vs. red). We compare the antibody landscape to NK landscapes in which each sequence site has an identical epistatic involvement (with exactly K coupled neighbors). In a smooth landscape (K=0, Fig. 6*B*, *Left*), success rate remains high until reaching a threshold close to 1; the steep drop reflects mutational load around the single fitness peak. Spatial structure results in monotonically decreasing success, as it slows spreading of beneficial mutations across the population. Antibody landscape exhibits a contrasting trend (Fig. 6*A*). First, success rate increases with occupancy threshold over a wide range, signaling transient occupancy of intermediate peaks. Second, spatial structure strongly boosts success at intermediate thresholds, suggesting a beneficial interplay of mutational and migratory constraints.

Attempting to generate antibody-like topography from rugged NK landscapes, we construct an NK ensemble (K=2) constrained to have the same number of fitness peaks and the same germline-to-$F_{\max}$ distance as the antibody landscape, such that only the location and height of local optima may differ (Fig. 6*B*, *Right*). While the ensemble-averaged success rate shows a qualitatively similar trend as the antibody landscape, both the level of success and the degree of enhancement due to spatial structure are much lower (note the log scale). We find that, among 50 independent realizations of constrained NK landscapes, only half support any success and very few show comparable success to the antibody landscape (*SI Appendix*, Fig. S6*A*). In addition, the threshold dependence varies considerably from one realization to another (*SI Appendix*, Fig. S6*B*). Hence, only rare instances of heterogeneous ruggedness enable efficient navigation.

To identify distinctive features of antibody landscape relevant to evolvability, we plot the stepwise distribution of fitness effect (DFE) of mutations, ΔF, along successful paths (Fig. 6*C*). Adaptation in constrained NK landscapes shows a similar DFE at every path step, reflecting homogeneous ruggedness across the landscape (DFE being independent of the genetic background). Interestingly, successful trajectories in the antibody landscape exhibit a strong fitness gain at the first step, followed by a rapid fall to small fitness increases, suggesting that heterogeneous ruggedness both supports, and steers toward, smooth adaptive paths to the global optimum—via diminishing-return epistasis. Spatial structure permits longer indirect paths.

To determine how heterogeneous ruggedness gives rise to smooth adaptive paths, we visualize the top-3 successful paths (with largest path weights), follow the occupancy of fitness peaks over time, and extract the stepwise ΔF along these paths (Fig. 6*D*–*F*). An unexpected picture emerges: without spatial structure (Fig. 6 *D* and *E*, *Upper* panels), successful populations bypass the local peaks (colored dots) and only enrich $F_{\max}$ to a considerable level (black curves in panel *E*). Migration constraints, however, speed population escape from local optima, making it possible to turn a road block into a stepping stone; 2D trajectories and stepwise fitness changes clearly show that all top paths first occupy then escape the stepping-stone fitness peak (orange dot) en route to $F_{\max}$ (Fig. 6*D*–*F*, *Lower* panels). In contrast, constrained NK landscapes lack smooth paths; populations are repeatedly trapped at local optima, resulting in a delayed arrival at $F_{\max}$ and a low occupancy (*SI Appendix*, Fig. S6*C*).

The strong boost of antibody evolvability due to spatial structure is attributable to an asymmetry between dominant paths under structured and well-mixed conditions (*SI Appendix*, Fig. S7): stepping-stone crossing paths successful in structured populations are highly prone to failure without structure. Conversely, road-block bypassing paths favorable in well-mixed conditions often remain viable to structured populations. From the viewpoint of competing attractors along the path, this boost can be understood from the maximum occupancy of local peaks vs. $F_{\max}$ (*SI Appendix*, Fig. S8). When migration constraints are applied, a high density of viable paths open at intermediate occupancy, where the enrichment of $F_{\max}$ exceeds the threshold whereas the maximum occupancy of local optima falls below (*SI Appendix*, Fig. S8 *B*, orange region and *D*, *Middle* panel). This explains why intermediate occupancy thresholds yield maximum success rates when populations are spatially structured.

Taken together, this epistatic landscape of antibody stability represents an instance of heterogeneous ruggedness shaped by an epistatic hotspot. An early mutation of the hotspot confers a large fitness gain which drives the population into effectively smooth paths of adaptation. Along these paths, suboptimal fitness peaks may serve as stepping stones that steer evolution toward the global optimum—the most stable antibody fold potentiated to evolve new functions.

## Discussion

The physics and evolution of proteins are inseparable. The synergistic changes in conformation by which proteins find the folded structure are a result of natural selection and atypical of random heteropolymers. Taking the converse view, the requirement of remaining foldable necessarily influences molecular evolution. Here, we attempt to quantify such influence by generating an empirical protein stability landscape and exploring the role of epistasis in shaping local and global trends of sequence evolution on this landscape.

When describing fitness landscapes in terms of peaks and valleys, we invoke an intuitive low-dimensional analogue of the high-dimensional genotype space. A major prediction originally formulated by Sewall Wright (21) is that, with an increasing genotypic dimensionality, the proliferation of local fitness maxima presents obstacles to adaptation and reduces accessibility of the global optimum. Yet, much recent progress made by considering empirical fitness landscapes, especially for biomolecules and microbial cells, highlights the role of high dimensionality in promoting accessibility (22–24), depicting biologically realistic fitness landscapes as being both rugged and navigable (6, 7). Despite increasing evidence and intriguing proposals, a theoretical understanding consistent with data is not yet gelling.

Here, we make a first step toward a mechanistic explanation for the concurring high ruggedness and high navigability in real genotype–fitness maps. Through mapping a combinatorially complete SARS-CoV-2 antibody folding-stability landscape, we draw a connection between global epistasis and evolvability via epistatic inequality among sequence sites. We find that sparse epistatic hotspots (whose state captures the global nonlinearity of an additive latent phenotype) give rise to heterogeneous ruggedness that, in turn, creates effectively smooth adaptive paths and enables efficient navigation toward the global optimum.

Our 10-site epistatic landscape reveals strong heterogeneity in topography, that is, local features are not representative of global patterns. Strikingly, such heterogeneity in ruggedness arises from inequality of epistatic importance among sequence sites. The data show that, in spite of pervasive epistatic interactions between mutations, correlations between fitness effects are both directed and remarkably sparse; an epistatic hotspot stands out as exerting influences on all other mutations without being reversely affected. Importantly, mutation of the hotspot simultaneously increases landscape ruggedness and enhances $F_{\max}$ accessibility.

To seek an explanation, we examine whether the hotspot mutation makes the adaptive paths taken more or less repeatable. Interestingly, a majority of successful paths that reach $F_{\max}$ within a limited time have the hotspot mutated at the first step and this ruggedness-increasing mutation reduces path diversity. Furthermore, path-focusing ruggedness steers populations to smooth directions, signaled by a rapid reduction in fitness gain in approach to the maximum fitness. This diminishing-return epistasis observed in the antibody landscape is absent from homogeneous model landscapes. These results suggest an organizing role of the epistatic hotspot; it shapes the high-dimensional landscape in such a way that exploits local constraints for global navigability. Indeed, since the hotspot dominates both the specific and global epistasis, its mutation may act like a switch for path-orienting topography.

**Fig. 7.**
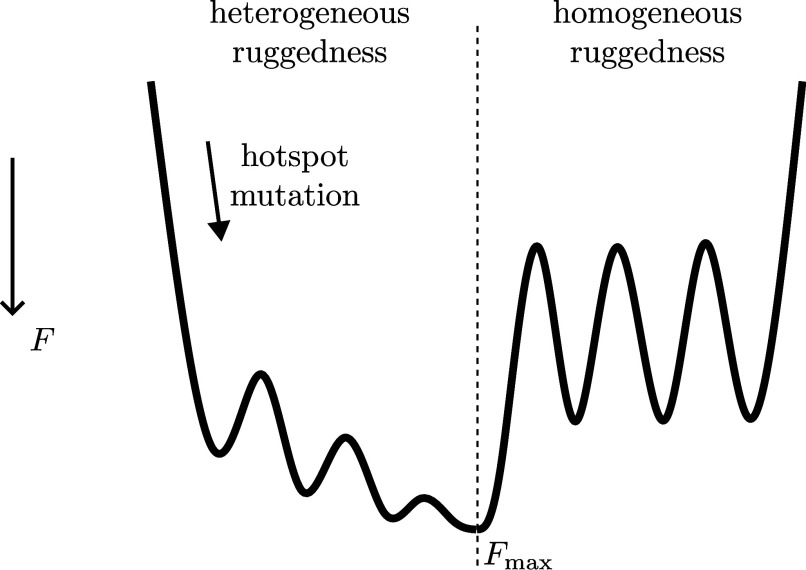
Summary sketch: Epistatic hotspot induces heterogeneous ruggedness that confers a funnel-like shape to fitness landscape. Left: An early mutation of the hotspot grants a large fitness gain and turns on path-orienting heterogeneous ruggedness. The dominant adaptive paths to the global optimum $F_{\max}$ are effectively smooth and characterized by diminishing return in fitness gain. Right: Homogeneous ruggedness, in contrast, would cause repeated trapping at local optima, hindering navigation toward $F_{\max}$. Fitness increases top–down.

A funneled energy landscape of protein folding provides a resolution to the Levinthal paradox (25, 26), allowing an efficient search of the vast configuration space to locate the native conformation, sometimes in microseconds. Here, we show that a heterogeneously rugged fitness landscape may enable productive navigation of sequence space to reach the global optimum. It is interesting to ask whether constructive epistasis resulting from a hotspot can organize fitness landscape into an inverted funnel-like shape. The global degree of funneling of a folding landscape has been quantified by the ratio of the folding temperature $T_{f}$ to the glass transition temperature $T_{g}$ (27–29), which compares the energy gap between the native state and compact misfolded states to the energy variance or ruggedness. A large $T_{f}/T_{g}$ indicates a strong funnel. An analogous metric characterizing epistatic landscapes is the slope-to-roughness ratio, s/r (*Materials and Methods*). A large s/r indicates a smooth, Mt. Fuji-like global shape, i.e., an inverted funnel. We find that antibody landscape has a larger s/r than any constrained NK landscape (*SI Appendix*, Fig. S10*A*), implying that heterogeneous ruggedness induced by the hotspot confers a funneling gradient. Further, hotspot mutation causes a large drop in s/r (*SI Appendix*, Fig. S10*B*), indicating the commencement of diminishing return in fitness gain. We sketch the physical picture in Fig. 7, contrasting heterogeneous with homogeneous ruggedness.

Our data suggest an alternative explanation for why global epistasis promotes evolvability. On one hand, statistical models often interpret the simple trend of dependence of fitness gain on the background fitness as a result of widespread modest interactions (30). Our results show that, global epistasis can also arise from sparse idiosyncratic interactions, if they were properly organized. On the other hand, more stable proteins are more evolvable because they are better able to tolerate destabilizing mutations that confer functional benefits (31). In this sense, as the hotspot mutation makes the most stable fold ($F_{\max}$) easier to access, it renders the antibody more evolvable. One way to test this connection is to study the folding-stability and binding-affinity landscapes of the same antibody, and see whether the hotspot, which governs the global shape of the stability landscape, promotes evolution of improved affinity for one or more target antigens. An epistatic landscape of antibody–antigen binding free energy was found to exhibit positive epistasis that enlarges the set of viable paths leading to high affinity antibodies (32). Finally, our analysis predicts that spatial structure that limits the range of competition may boost evolvability even further. This may rationalize why B lymphocytes form spatially segregated modest populations for antibody evolution (33)—as a means to balancing potency, diversity, and evolvability.

Prior research has suggested that pervasive epistatic interactions constrain protein evolution. A recent analysis of a DMS dataset on a transcription factor provided a counter example (34). Metzger and coworkers showed that pairwise epistasis facilitates the evolution of a new function (DNA specificity) by bringing variants with different functions close together in sequence space. Our work likewise identifies a productive role of epistasis in shaping the sequence–function map, with the following essential differences. First, changes in DNA specificity appeared to be largely captured by pairwise rather than higher-order interactions. In contrast, constructive organization of the antibody landscape relies on higher-order epistasis; without triplet interactions, the concomitant increase in ruggedness and navigability is lost (*SI Appendix*, Fig. S9 and section C5). Because it is the higher-order interactions that simplify the global shape through the creation of path-orienting ruggedness. Further, sparse hotspots induce heterogeneous ruggedness—key to the diminishing-return epistasis underlying the global smoothness. A reduced genetic distance between fitness peaks, however, can be achieved by an increase in homogeneous ruggedness (e.g., a larger K in NK landscapes) (35). More fundamentally, different physics underlie how epistasis is structured. Specific binding is relatively local in space. Correspondingly, a cluster of residues encoded by a large alphabet pairwise interact to form a dense net and produce a highly degenerate sequence–function map. Folding, however, is highly nonlocal. A hierarchy of hotspots concentrating the majority of mutational constraints may enable global coordination. As we saw, mutating a single hotspot residue stabilizes the antibody fold as a whole. Altogether, we propose an alternative physical underpinning of protein evolvability: sparse hierarchical epistatic hotspots organize easy-to-navigate protein stability landscapes; an enhanced ability to fold, in turn, facilitates the evolutionary search for new functional states. Antibodies may offer ample examples.

The scale-dependent ruggedness observed in the antibody landscape is reminiscent of a recent theoretical study of metabolic networks (36). When studying how epistasis propagates across scales, Kryazhimskiy found that, upon coarsening of subpathways, negative epistasis at smaller scales remains negative on larger scales, whereas positive epistasis may change sign. An immediate future direction is to construct a new family of epistatic models that exhibits high ruggedness and high accessibility. This will allow to address from bottom–up the minimal ingredients necessary for recapitulating the observed connection between heterogeneous ruggedness, global epistasis, and evolvability. While a variety of means exist for implementing hierarchical epistatic importance, contact with natural or laboratory systems will help direct modeling efforts.

Practically, the identification of epistatic hotspots (or more generally, highly epistatic regions) may guide the choice of targets in mutagenesis approaches: focusing mutations to strongly epistatic regions may yield adaptive paths otherwise too rare to be observed with a limited number of experiments. If present, the sparsity of epistatically important sites will allow a rapid assessment of a system’s complexity by nonexhaustive combinatorial mutagenesis, as suggested by Poelwijk et al. (37). This, in turn, provides an estimate for the number of phenotypic measurements necessary for a sufficiently accurate parameterization of the mapping.

Conceptually, an epistatic hotspot amid weakly epistatic sites can be viewed as a special case of a hierarchy with two levels. Given the ubiquity of hierarchical organization in biological systems, one can seek analogous impacts of the hotspot in broader contexts. Phillips et al. found that influenza antibodies can evolve breadth by acquiring hierarchical sets of epistatic interactions (38). This observation lends support for vaccination with sequential doses of sufficiently distinct but related antigen variants, a proposal made earlier from computational modeling of affinity maturation to elicit broadly neutralizing antibodies (39–41) and experimentally shown using SARS-CoV-2 spike antigens as an example (42). For exploratory adaptation of cells via phenotypic plasticity, a numerical study of gene regulatory networks showed that successful convergence of exploratory dynamics in high dimensions requires outgoing network hubs (43), in very much the same way that an outgoing epistatic hotspot organizes a complex yet navigable fitness landscape. Signaling networks of colonic stem cells provide evidence for the global impact of such hubs on cell-fate plasticity. Through mutational (cell-intrinsic) or microenvironmental (cell-extrinsic) perturbations, (dys)regulation of core signaling hubs may reshape the differentiation landscape and drive alternative cell fates (44). Future work will seek to establish a broader role of hierarchical epistatic hotspots in organizing high-dimensional solution spaces that permit efficient exploration.

## Materials and Methods

### Metrics of Landscape Ruggedness.

Ruggedness in a fitness landscape is a consequence of epistatic interaction between mutations. In a perfectly smooth landscape, individual mutations affect fitness in an additive manner. Thus, an intuitive metric of ruggedness would quantify the deviation from additivity through the loss in correlation of fitness effects of mutations; γ statistics (12) provide one way to do so (see details in *SI Appendix*, section E).

In its local form, $\gamma_{i\to j}=\mathrm{Corr}\left[\Delta_{j}\left(s\right),\Delta_{j}\left(s_{\left[i\right]}\right)\right]$, which measures to what degree the fitness effect of mutation j, $\Delta_{j}\left(s\right)=F\left(s_{\left[j\right]}\right)-F\left(s\right)$, is altered due to the presence of mutation i, $\Delta_{j}\left(s_{\left[i\right]}\right)=F\left(s_{\left[ij\right]}\right)-F\left(s_{\left[i\right]}\right)$, averaged over all genetic backgrounds. Here, $s_{\left[\cdots \right]}$ denotes the genotype with mutations at sites given in the brackets relative to s. $\gamma_{i\to j}\to 1$ corresponds to additivity hence smoothness, whereas $\gamma_{i\to j}\leq 0$ signals sign flip of fitness effects and, in turn, ruggedness. Thus, the matrix of $\gamma_{i\to j}$ constitutes a directed map of pairwise correlations. Since the γ metric averages over genetic backgrounds for each pair, the $\gamma_{i\to j}$ matrix can be relatively sparse despite a dense $J_{\mathrm{ij}}$ matrix. Compared to $\left\{J_{\mathrm{ij}}\right\}$ that depict genetic architecture (symmetric connectivity), $\left\{\gamma_{i\to j}\right\}$ reveal patterns of directional phenotypic impact and are thus well suited for identifying epistatic hotspots. To probe heterogeneity in ruggedness, we define a generalized measure, γ(n,d), to calculate the correlation in fitness effects of mutations between genotypes d mutations apart with n pinned sites, i.e., in (L−n)-dimensional sequence subspaces.

### Evolutionary Dynamics.

We simulated two types of dynamics, Markov chain Monte Carlo (MCMC) and Wright–Fisher (WF) dynamics, to emphasize the importance of landscape topography and paths taken by finite populations, respectively. See details in *SI Appendix*, section G1.

#### MCMC dynamics.

In the strong-selection weak-mutation (SSWM) limit, a population remains largely monomorphic and adaptation follows a series of fitness-increasing mutations until reaching a fitness peak. In this regime, adaptive dynamics can be characterized by an absorbing Markov chain with absorbing states at fitness optima. This simplification due to separation of timescales between mutation and selection allows an efficient evaluation of $F_{\max}$ accessibility and path entropy (see below): one can simulate evolutionary trajectories by sampling the mutational steps according to a $2^{L}\times 2^{L}$ transition matrix P, whose entries specify the probabilities of evolving from any given genotype to any single-residue mutant, determined solely by changes in fitness. One can represent P as a block matrix:

[3] $$\begin{array}{c} \begin{array}{c} P=\left(\begin{array}{cc} Q & R\\ 0 & I \end{array}\right), \end{array} \end{array}$$

where Q connects the transient states, R links any transient state to any absorbing state, and the zero and identity matrices define the absorbing states.

#### WF dynamics.

This is a more realistic scheme that permits fitness-valley crossing and clonal interference. Moreover, it allows to conveniently study how spatial structure affects evolutionary paths and outcomes. Inspired by Bergman et al. (45), we implement a minimal spatial structure by placing a population of size $N_{\mathrm{pop}}=500$ on a ring lattice of $N_{\mathrm{pop}}$ nodes. We advance an initially monomorphic population by iterating between i) selection and migration and ii) mutation. i) Denote by $s_{i}^{t}$ the genotype of the individual occupying node i at time t. At time t+1, it is replaced by a genotype $s_{j}^{t}$ in its r-neighborhood (j∈[i−r,i+r]) with the probability

[4] $$\begin{array}{c} \begin{array}{c} P\left(s_{i}^{t+1}=s_{j}^{t}\right)=\frac{\exp(F\left(s_{j}^{t}\right))}{\sum_{k=i-r}^{i+r}\exp\left(F\left(s_{k}^{t}\right)\right)}, \end{array} \end{array}$$

that is, a fitter individual is more likely to produce offspring which then migrate into a neighborhood of size r. Thus, r controls the spatial range of competition and dispersal in one generation. We set r=2 for a structured population and $r=N_{\mathrm{pop}}$ for a well-mixed population. ii) A lattice sweep of mutation in which each sequence locus of each individual is mutated (bit-flipped) with a probability $\mu =10^{-3}$. Hence, a mutation can at most spread r nodes in one generation.

### Evolutionary Paths.

We define the path taken by an adapting population according to the evolutionary dynamics it follows. For MCMC dynamics, a path is clearly defined by the order in which the set of mutations connecting the germline to $F_{\max}$ reach fixation. For WF dynamics, we adapted the definition of “lines of descent” (46) which represent the lineages that first arrive at the target genotype. This definition associates a unique path to each successful population that discovers $F_{\max}$ within a given time starting from the germline. In practice, we keep track of all mutation events in the forward simulations. Once $F_{\max}$ is reached, we trace the reverse sequence of mutations back to the germline and retrieve the path. Under both dynamics, we define the path weight $w_{\pi }$ as the frequency of path π in a simulated ensemble of replicate populations on the antibody landscape or the NK landscapes. To quantify path diversity, we calculate the Gibbs-Shannon entropy $S_{\pi }=-\sum_{\pi }w_{\pi }\ln w_{\pi }$, where $\sum_{\pi }w_{\pi }=1$. We scale $S_{\pi }$ by the maximum entropy that is attained when all distinct paths have an equal weight. See details in *SI Appendix*, section G2.

### Accessibility of the Global Fitness Optimum *F*_max_.

Evolutionary accessibility of a target genotype depends both on the availability of viable paths and on the chance by which each viable path is realized by an evolving population. While the former is primarily set by landscape topography as probed by MCMC dynamics, the latter is further influenced by the dynamics of landscape navigation as captured by WF dynamics. Accordingly, we devised two measures to quantify $F_{\max}$ accessibility (see details in *SI Appendix*, section G3).

#### Static accessibility.

Under MCMC dynamics, the absorbing probability of $F_{\max}$ can be calculated from Markov theory using the transition matrix (Eq. **3**); it measures to what extent the fitness landscape supports an adaptive walk from the germline to $F_{\max}$ without encountering any of the local optima. This metric is used to generate the histograms in Fig. 3*B* and *SI Appendix*, Figs. S5*C* and S9*D*.

#### Dynamic accessibility.

Under WF dynamics of finite populations, genetic drift becomes possible. We now characterize the navigation performance by the success rate at which a population first enriches $F_{\max}$ to an occupancy threshold as opposed to any local optimum within a given time $t_{\max}=2,000$. We estimate the success rate from a large number of replicate simulations starting from an isogenic population carrying the germline genotype. This metric is used in Fig. 6 *A* and *B* and *SI Appendix*, Fig. S6*B*.

### 2D Visualization of Landscapes and Paths.

Methods of dimensionality reduction allow to visualize a high-dimensional surface or trajectory in a low-dimensional embedding. To visualize the landscape topography such that genotypes of similar fitness are kept in proximity in embedding space, we use the force-directed graph layout. Specifically, we construct a network in which nodes represent genotypes and edges are drawn between mutational neighbors with a weight being inversely related to their fitness difference, $w_{ss^{\prime }}=(0.001+|F\left(s\right)-F\left(s^{\prime }\right){\left|\right)}^{-1}$, and then apply the force-directed layout (using function layout_drl from Python’s igraph package). To ensure that the embeddings for a pair of landscapes are comparable, we fix the inherent randomness by setting an identical random seed, and then perform a translation-rotation operation to maximize the overlap between embeddings, such that the remaining mismatch would reflect the actual difference in landscape topography (see examples in Fig. 1*E*). We use t-SNE to visualize the mutational paths realized in simulations, where Hamming distance serves as a natural metric of genotypic proximity. We use Python’s sklearn package (function sklearn.manifold.TSNE) to perform sequence-space embedding in two dimensions.

### Global Smoothness.

The roughness-to-slope ratio r/s has been introduced (47) to measure global ruggedness of an epistatic fitness landscape. We take its inverse, s/r, to draw an analogy to the ratio $T_{f}/T_{g}$ that characterizes the global degree of funneling in an energy landscape of protein folding (28). The idea is to fit the fitness landscape of interest F(s) by the closest smooth landscape described by an additive model $F^{\left(a\right)}\left(s\right)=\sum_{i=1}^{L}h_{i}^{\left(a\right)}s_{i}$. Then the mean “slope” $s=L^{-1}\sum_{i=1}^{L}\left|h_{i}^{\left(a\right)}\right|$ from the linear model is compared to the “roughness” $r=2^{-L/2}\sqrt{\sum_{s}{\left(F\left(s\right)-F^{\left(a\right)}\left(s\right)\right)}^{2}}$ due to residual fitness effects (i.e., the overall nonadditivity) to quantify the global smoothness of F(s). A landscape with a greater s/r would be more navigable by evolution. We obtain the linear coefficients $\left\{h_{i}^{\left(a\right)}\right\}$ through a least-square fit that minimizes the square error between the actual landscape F(s) and the smooth landscape $F^{\left(a\right)}\left(s\right)$. For the antibody landscape, we use the inferred specific model (Eq. **1**). For each realization of the constrained NK landscapes, we ensure that the wild-type genotype has zero fitness before performing the fit.

## Supplementary Material

Appendix 01 (PDF)

## Data Availability

Raw deep sequencing data and custom code have been deposited in NIH Sequence Read Archive and GitHub PRJNA755438 (48), https://github.com/nicwulab/COV107-23_fitness_landscape (49), and https://github.com/st-sch/landscape_inference (50)).
