# Supplementary material for: Epistatic hotspots organize antibody fitness landscape and boost evolvability
Source: Proc Natl Acad Sci U S A. 2025 Jan 8;122(2):e2413884122. doi: 10.1073/pnas.2413884122 (PMC11745389; doi:10.1073/pnas.2413884122)
Supplement: Supplementary file 1 — Appendix 01 (PDF) [file pnas.2413884122.sapp.pdf]

Supplementary Information: Epistatic hotspots organize antibody fitness landscape and boost evolvability

Steven Schulz,[1,*] Timothy J.C. Tan,[2,*] Nicholas C. Wu,[2,3,4] and Shenshen Wang[1]

[1] *Department of Physics and Astronomy, University of California, Los Angeles, Los Angeles, CA 90095, USA*
[2] *Center for Biophysics and Quantitative Biology, University of Illinois at Urbana-Champaign, Urbana, IL 61801, USA*
[3] *Department of Biochemistry, University of Illinois at Urbana-Champaign, Urbana, IL 61801, USA*
[4] *Carl R. Woese Institute for Genomic Biology, University of Illinois at Urbana-Champaign, Urbana, IL 61801, USA*

A. EXPERIMENTAL METHODS

1. COV107-23 antibody library design

COV107-23 is a IGHV3-53/3-66-encoded germline antibody with strong affinity to SARS-CoV-2 receptor binding domain (RBD). As part of the adaptive immune response to a pathogen, germline antibodies are further improved via affinity maturation, a fast-paced evolutionary process cycling somatic hypermutation (SHM) and selection for fitter variants, to increase stability and/or affinity for the antigen. To define the sequence diversity likely targeted by affinity maturation on IGHV3-53/3-66-encoded antibodies, we mined and ranked frequently retained SHMs in IGHV3-53/3-66-encoded antibodies isolated from across a multitude of independent immune responses against SARS-CoV-2 reported in the literature [1–18], leaving aside antibodies with distinctly elongated HCDR3 ($\geq$ 15 amino acids) as they resort to alternative binding modes [19]. The 12 most frequent IGHV3-53/3-66-encoded, SARS-CoV-2 specific SHMs from across 10 distinct sites (5 sites in HCDR1, 5 sites in HCDR2) display a wide range of occurrence frequencies (Fig. S1). These SHMs also have diverse structural roles (Fig. S2 and Table S1). To probe their epistatic non-equivalence and heterogeneous contributions to antibody fitness (Fig. 1), we constructed a combinatorial mutant library of COV107-23, which consisted of all possible combinations of these 12 SHMs. Of note, in Fig. S2 and Table S1, the structure of CC12.1 with spike RBD was used (PDB: `6XC2`) because we do not have a structure of COV107-23 in complex with spike RBD. Nonetheless, both belong to the same IGHV3-53/3-66 clonotype [20]. We define and build a COV107-23 antibody library around this set of recurrent somatic mutations.

2. Construction of pCTcon2-COV107-23

The sequence of the heavy and light chains of COV107-23 was obtained from Robbiani *et al.* [8] (named COV107_Plate2_HC_23-P1369, Table S2). The heavy chain of COV107-23 was PCR-amplified using COV107_HC, COV107_HC_F and COV107_HC_R as template, forward primer and reverse primer, respectively. The ERBV site was PCR-amplified using pCTcon2_B382, ERBV_F and ERBV_R as template, forward primer and reverse primer, respectively. The light chain of COV107-23 was PCR-amplified using COV107_KC, COV107_KC_F and COV107_KC_2R as template, forward primer and reverse primer, respectively. All three PCR products were gel-purified using a Monarch DNA gel extraction kit (New England BioLabs). Primer sequences are shown in Table S3.

To generate the insert, 10 ng each of the gel-purified PCR products described above were mixed with COV107_KC_F and COV107_HC_R as forward primer and reverse primer, respectively, for overlap PCR. The PCR product was gel-purified. The vector was PCR-amplified using pCTcon2_B38 [20], COV107_SHMlib_VF and COV107_SHMlib_VR as template, forward primer and reverse primer, respectively. The PCR product was column-purified using a PureLink PCR purification kit (Invitrogen). NEBuilder Hifi DNA assembly kit (New England BioLabs) was used to generate pCTcon2-COV107-23, which encodes the COV107-23 antibody to be displayed on yeast cell surface, with 100 ng of vector DNA and a 2 : 1 insert:vector molar ratio, following the manufacturer's protocol. The final pCTcon2-COV107-23 plasmid has the following in-frame: COV107-23 variable kappa chain, COV107-23 constant kappa chain, V5 tag, ERBV site, Aga2p-SS, COV107-23 variable heavy chain, COV107-23 CH1 heavy chain, HA-tag, 218 linker, Aga2p.

---

* equal contribution

3. Construction of COV107-23 antibody yeast display library

Heavy chain variant oligos were generated via successive rounds of PCR. In the first round, pCTcon2-COV107-23, COV107_SHMlib_F1 and COV107_SHMlib_R1 were used as the template, forward primer and reverse primer, respectively. The PCR product was gel-purified. Subsequently, the gel-purified product from the first round of PCR was used as template for the second round of PCR with COV107_SHMlib_F2 and COV107_SHMlib_R2 as the forward primer and reverse primer, respectively. The PCR product was then column-purified. Finally, the column-purified product from the second round of PCR was used as template for the last round of PCR with COV107_SHMlib_F3 and COV107_SHMlib_R3 as the forward primer and reverse primer, respectively. The final PCR product, which contains the 2048 heavy chain variants, was column-purified. Of note, COV107-23 antibodies express an HA-tag.

The antibody yeast display library was generated as previously described [21]. Briefly, *Saccharomyces cerevisiae* EBY100 (American Type Culture Collection) cells were grown in 10 ml YPD medium (1 % w/v yeast extract, 2 % w/v peptone, 2 % w/v dextrose, all dissolved in deionized water and autoclaved) by incubating at 30 °C with shaking at 225 rpm. Once OD_{600} has reached 3, an aliquot of the culture was incubated in 100 ml of YPD medium such that the initial OD_{600} is 0.3, with shaking at 225 rpm at 30 °C. When OD_{600} has reached between 1.3 and 1.6, cells were centrifuged at 1,700×g for 3 min at room temperature. Supernatant was discarded and cells were washed twice with 50 ml ice-cold water and then once with 50 ml ice-cold electroporation buffer (1 M sorbitol, 1 mM calcium chloride). After the final wash, cells were resuspended in 20 ml of conditioning media (0.1 M lithium acetate, 10 mM dithiothreitol) with shaking at 225 rpm at 30 °C for 30 min. Cells were collected via centrifugation at 1,700×g for 3 min at room temperature and washed once with ice-cold electroporation buffer. The pellet was resuspended in ice-cold electroporation buffer to reach a final volume of 1 ml and then kept on ice. 5 µg of purified pCTcon2-COV107-23 vector and 4 µg of the amplified heavy chain variant DNA oligos were added into 400 µl of conditioned yeast. The mixture was transferred into a pre-chilled BioRad Gene Pulser cuvette with a 2 mm electrode gap and kept on ice for 5 min. Cells were electroporated using an ECM 630 electroporator (BTX) at 2.5 kV and 25 µF, with time constants ranging from 3.8 µs to 4.1 µs. Electroporated cells were added to 4 ml of YPD with 4 ml of sorbitol, and incubated at 30 °C for 1 h with shaking at 225 rpm. Cells were then centrifuged at 1,700×g for 3 min at room temperature. The pellet was resuspended in 600 µl of SD-CAA medium (2 % w/v dextrose, 0.67 % w/v yeast nitrogen base with ammonium sulfate, 0.5 % w/v casamino acids, 0.54 % w/v Na_2HPO_4, 0.86 % w/v NaH_2PO_4, all dissolved in deionized water and autoclaved), plated onto SD-CAA plates (2 % w/v dextrose, 0.67 % w/v yeast nitrogen base with ammonium sulfate, 0.5 % w/v casamino acids, 0.54 % w/v Na_2HPO_4, 0.86 % w/v NaH_2PO_4, 18.2 % w/v sorbitol, 1.5 % w/v agar, all dissolved in deionized water) and incubated at 30 °C for 40 h. Colonies were subsequently collected in SD-CAA medium and centrifuged at 1,700×g for 5 min at room temperature. The pellet was resuspended in SD-CAA medium with 15 % v/v glycerol such that OD_{600} is 50. Glycerol stocks were stored at −80 °C.

4. Fluorescence-activated cell sorting of COV107-23 antibody variants

Sorting of the yeast antibody display library was performed as previously described [20]. Briefly, 100 µl of COV107-23 antibody yeast display library glycerol stock was recovered in 50 ml of SD-CAA medium by incubating yeast at 27 °C with shaking at 250 rpm. Once OD_{600} has reached between 1.5 and 2.0, 15 ml of the culture was harvested via centrifugation at 4,000×g at 4 °C for 5 min. The supernatant was discarded and the pellet was resuspended in 50 ml of SGR-CAA medium (0.1 % w/v dextrose, 2 % w/v galactose, 2 % w/v raffinose, 0.67 % w/v yeast nitrogen base with ammonium sulfate, 0.5 % w/v casamino acids, 0.54 % w/v Na_2HPO_4, 0.86 % w/v NaH_2PO_4, all dissolved in deionized water and autoclaved). Yeast culture was transferred to a baffled flask and incubated at 18 °C with shaking at 250 rpm until OD_{600} has reached between 1.3 and 1.6. 5 ml of culture was harvested and the pellet obtained via centrifugation at 4,000×g at 4 °C for 5 min. The pellet was washed with 5 ml of 1 x PBS twice and then resuspended in 1 ml of 1 x PBS.

Then, 1 µg/ml of PE anti-HA.11 (epitope 16B12, BioLegend, Cat. No. 901517) buffer-exchanged into 1 x PBS was added to washed yeast. A negative control was set up with an equal volume of 1 x PBS added into washed yeast. Cells were incubated at 4 °C overnight with rotation. Cells were subsequently washed twice with 5 ml of 1 x PBS, resuspended in 3 ml of 1 x PBS and transferred to FACS tubes. All PE-positive cells were collected in 1 ml of SD-CAA with 1 x penicillin/streptomycin using a BD FACS Aria II cell sorter (BD Biosciences). Cells were centrifuged at 1,700×g at 20 °C for 15 min. The supernatant was discarded. Then, cells were resuspended in 500 µl SD-CAA medium and incubated at 30 °C with shaking at 200 rpm overnight. Cells were centrifuged at 1,700×g at 20 °C for 5 min. The supernatant was discarded, and the pellet resuspended in 15 % v/v glycerol in SD-CAA to generate frozen stocks with OD_{600} of 20.

5. Next generation sequencing of COV107-23 antibody variants

Plasmids from the unsorted yeast display library (input) and two replicates of the sorted yeast display library were extracted using a Zymoprep Yeast Plasmid Miniprep II kit (Zymo Research) following the manufacturer's instructions. The heavy chain region of interest was PCR-amplified using the extracted plasmids, COV107_SHMlib_recoverF and COV107_SHMlib_recoverR as template, forward and reverse primers, respectively. Adapters containing sequencing barcodes were added to the genes encoding the heavy chain region via PCR. 100 ng of each sample was used for paired-end sequencing using MiSeq PE150 or PE250 (Illumina). Raw reads can be accessed at BioProject accession number PRJNA755438.

Using custom Python code, regions corresponding to the region of interest in the heavy chain was extracted from each paired read. The number of reads of each variant was counted. Reads that had mutations in addition to mutations in this study were discarded because they are caused by errors during amplification or sequencing. A pseudocount of 1 was added to the final count to prevent division by zero when calculating enrichment values. Enrichment was calculated by dividing the number of reads in expression, and the number of reads in input. Code to analyze deep sequencing data can be accessed at https://github.com/nicwulab/COV107-23_fitness_landscape.

Information reported from the combinatorial mutagenesis experiment includes sequencing reads of the yeast population before and after the sorting step for each of $4 \times 2^9 = 2048$ variants. Two sets of counts are obtained from two independent replicates of the experiment (selection and sequencing).

6. Prediction of $\Delta\Delta G$ of COV107-23 and its single mutants

The $\Delta\Delta G$ values of COV107-23 and its mutants were predicted using the "ddG_monomer" application [22] in Rosetta (RosettaCommons). The monomer of COV107-23, which includes its heavy chain and light chain, was renumbered using the "pdb_reres.py" application in PDB Tools. The structure was pre-minimized using the high-resolution protocol of the "ddG_monomer" application in Rosetta and was subsequently used to predict $\Delta\Delta G$ values, with 30 poses each for wild-type and mutant COV107-23. Files including mutation files and flags to run the application can be accessed at https://github.com/nicwulab/COV107-23_fitness_landscape.

7. Frustration analysis

Frustration analysis was performed on wild-type and mutated versions of three sites in the heavy chain of COV107-23 – sites 53, 35, and 27. Point mutagenesis to generate the `F27I`, `F27L`, `F27V`, `S35T`, or `S53P` mutation was performed using the fixed backbone application (PMID: 14631033) in Rosetta (RosettaCommons). One-hundred poses in total were generated, and the lowest scoring pose for each mutation was used for frustration analysis. Wild-type or mutant COV107-23 was uploaded to the Frustratometer server (PMID: 27131359). Heavy and light chains of COV107-23 were selected; no electrostatics was introduced for frustration analysis. Results from configurational single-residue frustration indices and contact analyses were plotted in R. Associated structural analyses were visualized in Pymol.

Site 53, which is a hotspot, when mutated from serine to proline, has a lower frustration index (i.e. more frustrated) than that of wild-type (Fig. S11A, pink arrow). P53 has a highly frustrated contact (red line), whereas this is absent in S53 (Fig. S12A). Despite the lower frustration index of P53 compared to that of S53, P53 can rigidify the HCDR2 loop, which is mainly composed of serines and glycines. Even though this can result in the loss of a hydrogen bond between S53 of the antibody and Y473 of the viral spike (Fig. S13A), the rigidification of the HCDR2 loop can stabilize the β strands comprising the framework regions of the antibody, and permit the formation of two hydrogen bonds between S56 of the antibody and N460 of the spike (Fig. S13B).

Site 35, which is a not a hotspot, does not have any significant difference in the frustration index between wild-type and mutant (Fig. S11B, grey arrow). Both S35 and T35 have one highly frustrated contact and one minimally frustrated contact (Fig. S12B, red and green lines, respectively).

Site 27, which has positive ΔF and $\Delta\Delta G$ values, has a higher frustration index (i.e. lower frustration) for its mutant compared to the wild-type (Fig. S11C, green arrow). Wild-type and mutant site 27 have two minimally frustrated contacts (Fig. S12C, green lines). In wild-type COV107-23, F27 of the heavy chain can form a cation-pi interaction with N487 of the spike (Fig. S13A). Mutating F27 to isoleucine, leucine, or valine, results in the loss of the cation-pi interaction with the spike, but can form relatively weaker Van der Waals interactions with N487 of the spike (Figs. S13C-E). Nonetheless, these mutations decrease the frustration level compared to the wild-type.

B. FITNESS LANDSCAPE MODELS

1. Models of specific and global epistasis

One can infer genotype-fitness maps from combinatorial mutagenesis data by assuming some underlying structure of a statistical model. We begin with a non-epistatic model: Given a sequence of binary sites s_i with $i = 1$ to L and the associated fitness $F(\mathbf{s})$, the non-epistatic model is

$$F(\mathbf{s}) = \sum_{i=1}^{L} h_i s_i. \tag{1}$$

Here $s_i = 1$ if the amino acid at site i is mutated and $s_i = 0$ otherwise. Thus, the wild-type or germline genotype has zero fitness. While additive models often explain much of the variance in the genotype-fitness maps, there is usually a significant portion of unexplained variance [23]. We consider two ways to incorporate epistasis into this additive model: specific and global epistasis.

I. Specific epistasis model

Models of specific epistasis include a hierarchy of interactions of biallelic loci at increasing orders:

$$F(\mathbf{s}) = \sum_{i=1}^{L} h_i s_i + \sum_{i=1}^{L}\sum_{j=1}^{i-1} J_{ij} s_i s_j + \sum_{i=1}^{L}\sum_{j=1}^{i-1}\sum_{k=1}^{j-1} K_{ijk} s_i s_j s_k + \ldots \tag{2}$$

where the coupling strengths J_{ij} and K_{ijk} quantify epistatic interactions of concurring mutations in pairs and triplets, respectively. More generally, an nth order epistatic term is the degree to which an $(n-1)$th order term depends on the context of yet another mutation. We later describe how to truncate the hierarchy through cross-validation and to infer the epistatic coupling strengths through maximum likelihood estimation (Section C).

II. Global epistasis model

A distinct approach introduces a different form of epistasis, that is, a global coupling of all sites that determines the observed phenotype. Specifically, an additive latent phenotype maps nonlinearly to fitness

$$F(\mathbf{s}) = g(\phi(\mathbf{s}))\,, \qquad \phi(\mathbf{s}) = \sum_{i=1}^{L} h_i s_i. \tag{3}$$

Here ϕ is an unobserved additive trait to be inferred from data, and $g(\phi)$ is a nonlinear function that represents the shape of global epistasis. This form of mapping is motivated by a smooth nonlinearity often observed between empirical fitness data and their best-fit non-epistatic models. The underlying assumption of this model is that mutations affect the observed phenotype only through altering a single latent phenotype. Candidates for $g(\phi)$ are parametric families of monotonic functions, such as splines (polynomials) [24] and sigmoids [25]. We describe below how to simultaneously infer the additive effects $\{h_i\}$ and the global nonlinearity $g(\phi)$ using standard maximum likelihood (Section C).

2. The NK model of tunably rugged landscapes

We choose to represent tunably rugged fitness landscapes using the NK model [26], a broad family of model landscapes for studying protein evolution in a static [26] or dynamic [27] environment. One key feature of the NK model is the statistical equivalence of sequence loci (to be detailed below), making it a useful null model against which we identify distinctive features of the antibody stability landscape. These features would signify varying epistatic importance across sequence loci.

In an NK landscape, the fitness $F(\mathbf{s})$ of a genotype represented by a bit string $\mathbf{s}$ of length L is defined as the average over the fitness contribution of each bit:

$$F(\mathbf{s}) = \frac{1}{L}\sum_{i=1}^{L} f_i(s_i, s_{i_1}, \ldots, s_{i_K})\,. \tag{4}$$

Here the fitness contribution f_i of site i depends on $\{s_i, s_{i_1}, \ldots, s_{i_K}\}$, the state of $K+1$ coupled sites that act like a cooperative unit. Each of the $2^{K+1}L$ fitness values is drawn independently from $\mathcal{U}[0,1)$ and remains fixed. Note that statistical properties of landscape topography are insensitive to the distribution of single-site fitness. By construction, the NK model represents sparse epistasis in the sense that, while all sequence loci contribute to fitness, their mutations are involved in fewer epistatic interactions than theoretically possible.

Upon a point mutation, on average $(K+1)$ out of L terms in the sum of Eq. (4) change their value. Hence, K controls the degree of fitness correlation between neighboring genotypes. Since K is identical across loci, it tunes the global level of ruggedness. An additive landscape ($K=0$) has a single global optimum reachable from any starting genotype, whereas in a completely random landscape ($K=L-1$), uncorrelated fitness values of nearby genotypes yield an extremely rough surface in which on average $2^L/(L+1)$ local maxima may be surrounded by fitness valleys. Realistic landscapes are bounded between these extremes.

Extensions of the classical NK model have been proposed to incorporate real-world features, such as neutrality [28, 29] and modularity [30]. A comparison of antibody landscape to tunably rugged NK landscapes with homogeneous statistical properties allows us to characterize hotspot-induced heterogeneity and its impact on protein evolvability.

C. PARAMETER INFERENCE AND MODEL VALIDATION

Our goal is to infer and validate epistatic models of the antibody fitness landscape using the sequencing reads before and after selection (FACS) as the sole input.

1. Empirical fitness landscape: log-enrichment of sequencing reads

One can obtain an empirical fitness landscape from the combinatorial mutagenesis data by taking log-enrichment in protein expression levels due to selection as the fitness value. This reflects that the FACS assay retains individual yeast cells according to the presence of a fluorescence tag indicating the expression of a properly folded protein. In the regime of unsaturated folding stability and deterministic selection, fitness is directly related to the logarithm of the probability of folding and hence enrichment. Thus, the fitness of a sequence $\mathbf{s}$ with respect to that of a reference sequence $\mathbf{s}_0$ can be defined as

$$F_{\text{emp}}(\mathbf{s}) = \ln\left(\frac{n_1(\mathbf{s})+1}{n_0(\mathbf{s})+1}\right) - \ln\left(\frac{n_1(\mathbf{s}_0)+1}{n_0(\mathbf{s}_0)+1}\right). \tag{5}$$

Here $\{n_0(\mathbf{s})\}$ and $\{n_1(\mathbf{s})\}$ represent sequence abundances (sequencing reads) before and after selection, respectively. Note that a pseudo-count $+1$ is included in both the pre- and post-selection reads to avoid divergence at zero count. The second term ensures zero fitness at the germline genotype. However, this approach propagates experimental noise due to a finite sequencing depth and may exaggerate the level of ruggedness present in the fitness landscape. We use two approaches to filter spurious ruggedness from actual epistasis in the specific epistasis model (Eq. (2)): (i) unsupervised learning based on maximum-likelihood estimation and cross-validation and (ii) Walsh-Hadamard transform. Effectiveness of denoising manifests as a stronger correlation between inferred landscape models ($R^2 = 0.98$) than between empirical landscapes ($R^2 = 0.86$) from two replicate experiments (Fig. S3B, top row).

2. Unsupervised learning of specific epistasis

I. *Maximum-likelihood estimation*

We aim to estimate the parameters of the specific epistasis model given by Eq. (2) through unsupervised learning based on maximum likelihood using only the sequencing reads before and after selection. Since the experiment consists of selection, amplification and sequencing stages, our inference method constitutes a probabilistic description of this procedure.

Following the steps in [31], we express the post-selection sequence frequencies $f_1(\mathbf{s})$ in terms of the pre-selection frequencies $f_0(\mathbf{s})$ and fitness values $F(\mathbf{s})$ as follows

$$f_1(\mathbf{s}) = \frac{f_0(\mathbf{s})\mathrm{e}^{F(\mathbf{s})}}{\sum_{\mathbf{s}'} f_0(\mathbf{s}')\mathrm{e}^{F(\mathbf{s}')}} = \frac{n_0(\mathbf{s})\mathrm{e}^{F(\mathbf{s})}}{\sum_{\mathbf{s}'} n_0(\mathbf{s}')\mathrm{e}^{F(\mathbf{s}')}}, \tag{6}$$

where the denominator ensures normalization of frequencies, i.e. $\sum_{\mathbf{s}} f_1(\mathbf{s}) = 1$, and the second equality uses $f_0(\mathbf{s}) = n_0(\mathbf{s})/\sum_{\mathbf{s}'} n_0(\mathbf{s}')$. Note that $f_1(\mathbf{s}) = n_1(\mathbf{s})/\sum_{\mathbf{s}'} n_1(\mathbf{s}')$. Here we assume deterministic selection since a large quantity of yeast cells is present initially. We then consider stochastic amplification of the selected pool, since a relatively small number of yeasts remain after selection. We thus draw the sequencing reads after amplification from a multinomial distribution, with epistatic coefficients contained in the expression of $F(\mathbf{s})$ as parameters:

$$P\left[\{n_1(\mathbf{s})\}|\{n_0(\mathbf{s})\},\{F(\mathbf{s})\}\right] = \frac{(\sum_{\mathbf{s}} n_0(\mathbf{s}))!}{\prod_{\mathbf{s}} n_1(\mathbf{s})!}\prod_{\mathbf{s}} f_1(\mathbf{s})^{n_1(\mathbf{s})} = \frac{(\sum_{\mathbf{s}} n_0(\mathbf{s}))!}{\prod_{\mathbf{s}} n_1(\mathbf{s})!}\prod_{\mathbf{s}}\left(\frac{n_0(\mathbf{s})\mathrm{e}^{F(\mathbf{s})}}{\sum_{\mathbf{s}'} n_0(\mathbf{s}')\mathrm{e}^{F(\mathbf{s}')}}\right)^{n_1(\mathbf{s})}. \tag{7}$$

Parameter inference is carried out by maximizing the likelihood of data (yeast abundance for each antibody sequence). That is, we obtain the parameter values $\{\hat{h}_i\}, \{\hat{J}_{ij}\}, \dots$ that maximize the following log-likelihood function

$$\mathcal{L}(\{h_i\},\{J_{ij}\},\dots|\{n_0(\mathbf{s})\},\{n_1(\mathbf{s})\}) = \sum_{\mathbf{s}} n_1(\mathbf{s})F(\mathbf{s}) - \left(\sum_{\mathbf{s}} n_1(\mathbf{s})\right)\ln\left(\sum_{\mathbf{s}} n_0(\mathbf{s})\mathrm{e}^{F(\mathbf{s})}\right) \tag{8}$$

which derives immediately from Eq. (7) and where all additive terms independent of model parameters are ignored. Note that Eq. (8) is invariant under the transformation $F(\mathbf{s}) \leftarrow F(\mathbf{s}) + \epsilon$, yet Eq. (2) fixes the gauge ϵ by enforcing that the germline genotype has zero fitness. We perform numerical minimization of $(-\mathcal{L})$ using the `minimize` function from Python's `scipy.optimize` package with the BFGS method.

II. Cross-validation

From the full dataset of 2^L measured phenotypes (fitness values), one can compute the full hierarchy of 2^L epistatic interactions between L mutated loci. This reflects the fact that, mathematically, epistasis is a transform in which phenotypes of individual variants are represented as context-dependent effects of the underlying mutations. Yet, the amount of information linking genotype to phenotype may be low compared to the theoretical limit, necessitating only a subset of epistatic terms for reconstructing the phenotype measurements. Moreover, stochasticity in the sampling step prior to sequencing as well as potential overfitting is not yet fully accounted for. We address these considerations via cross-validation of the inferred model. Specifically, we perform 10-fold cross-validation for various choices of P, the order at which to truncate the epistatic hierarchy in Eq. (2), and choose the value of P that yields the best performance in test-set prediction. We find that the specific epistasis model with the maximum predictive power contains up to third-order interactions (i.e. $P = 3$); see Fig. S3A top panel.

Fitting the specific epistasis model truncated at the third order to the full dataset ($2^{10} = 1024$ phenotypes) achieves a good quality of fit against $F_{\mathrm{emp}}(\mathbf{s})$ ($R^2 = 0.85$, Fig. S3B lower right), which is on par with the reproducibility of the experiment itself ($R^2 = 0.86$, Fig. S3B upper left). In addition, the inferred fitness values are highly reproducible across experimental replicates ($R^2 = 0.98$, Fig. S3B upper right). This suggests that the model is effective at capturing the signal while filtering the noise.

3. Walsh-Hadamard transform

The Walsh-Hadamard transform [23] provides an alternative approach to filter experimental noise from true epistasis. It is a generalized class of Fourier analysis by which one can decompose a fitness landscape into epistatic interaction of different orders. In the case of an empirical fitness landscape obtained from log-enrichment of antibody expression levels, the inferred parameters of a specific epistasis model can be interpreted as the Fourier coefficients.

Eq. (2) can be rewritten as

$$F(\mathbf{s}) = \sum_{p=1}^{P}\left(\sum_{i_1=1}^{L}\sum_{i_2=1}^{i_1-1}\cdots\sum_{i_p=1}^{i_{p-1}-1}\hat{f}_{i_1 i_2\dots i_p} s_{i_1}s_{i_2}\dots s_{i_p}\right), \tag{9}$$

where p labels the interaction order that runs up to order P at which we seek to truncate the model; the coefficients $\hat{f}_{i_1 i_2\dots i_p}$ replace h_i, J_{ij}, etc, at each corresponding order. Applying the transform, the fitness function $F(\mathbf{s})$ defined on the L-dimensional hypercube can be expressed as

$$F(\mathbf{s}) = \sum_{\mathbf{k}} \hat{f}_{\mathbf{k}}(-1)^{\mathbf{s}\cdot\mathbf{k}}, \tag{10}$$

where the sum runs over $\sum_{p=0}^{L}\binom{L}{p}=2^L$ possible "wave vectors" $\mathbf{k}\in\{0,1\}^L$ and $\mathbf{s}\cdot\mathbf{k}=\sum_{i=1}^{L}s_ik_i$. The coefficients $\hat{f}_\mathbf{k}$ are the Walsh-Hadamard transform of $F(\mathbf{s})$ with respect to the basis function $(-1)^{\mathbf{s}\cdot\mathbf{k}}$. They are given by

$$\hat{f}_\mathbf{k}=\frac{1}{2^L}\sum_{\mathbf{s}}F(\mathbf{s})(-1)^{\mathbf{s}\cdot\mathbf{k}}. \tag{11}$$

Terms involving wave vectors $\mathbf{k}$ with a large $\sum_{i=1}^{L}k_i$ correspond to high-frequency modes (i.e. experimental noise), whereas those with a small $\sum_{i=1}^{L}k_i$ represent the actual signal.

For the empirical fitness landscape $F_{\mathrm{emp}}(\mathbf{s})$ defined in Eq. (5) we compute the Hadamard spectrum $\hat{f}_\mathbf{k}$ given by Eq. (11). To quantify the relative contribution of epistatic interaction of order p, we calculate the rescaled amplitude spectrum [32]

$$\beta_p=\frac{1}{\binom{L}{p}}\sum_{\substack{\mathbf{k}\\ \sum_{i=1}^{L}k_i=p}}\hat{f}_\mathbf{k}^2. \tag{12}$$

As shown in Fig. S3A lower panel, this amplitude saturates at a low but non-zero value for $p>3$, indicating the dominance of noise above the third order. This is consistent with what we found from unsupervised learning and cross-validation ($P=3$). We thus apply a band-pass filter to the inverse Hadamard transform defined in Eq. (10), by setting $\hat{f}_\mathbf{k}=0$ for all wave vectors $\mathbf{k}$ that satisfy $\sum_{i=1}^{L}k_i>3$, to obtain $F(\mathbf{s})$.

The epistasis model inferred from Walsh-Hadamard transform correlates well with that obtained by unsupervised learning ($R^2=0.85$), confirming that truncation at the third order of epistatic interaction yields a best fit to the empirical fitness landscape. Yet unsupervised learning is superior to Walsh-Hadamard transform in the overall performance in fitting $F_{\mathrm{emp}}(\mathbf{s})$ (after third-order truncation, $R^2=0.85$ vs 0.75; see Fig. S3B lower right).

4. Unsupervised learning of global epistasis

We use the `mavenn` package [25] to infer a model of global epistasis. It represents the global nonlinearity as a sum of tanh sigmoids

$$g(\phi)=\alpha+\sum_{m=1}^{M}\zeta_m\tanh(\delta_m\phi+\epsilon_m), \tag{13}$$

where α, $\zeta_m\geq 0$, $\delta_m\geq 0$ and ϵ_m for $m=1,...,M$ are parameters to be determined along with the local fields $\{h_i\}$ through maximum-likelihood estimation using a Gaussian noise model,

$$P[\{F_{\mathrm{emp}}(\mathbf{s})\}|\{h_i\},\alpha,\{\zeta_m,\delta_m,\epsilon_m\}]=\frac{1}{\sqrt{2\pi}\sigma}\exp\left(-\frac{(F_{\mathrm{emp}}(\mathbf{s})-F(\mathbf{s}))^2}{2\sigma^2}\right), \tag{14}$$

where $F(\mathbf{s})$ is given by Eqs. (3) and (13). We find that including nonlinear channels beyond $M=1$ has negligible effect on the performance of the inferred model. We thus choose $g(\phi)=\alpha+\zeta\tanh(\delta\phi+\epsilon)$ to obtain Fig. 1B.

5. Importance of higher-order epistasis

Our specific-epistasis model and inference procedure belong to the broad class of statistical inference methods on the basis of the maximum-entropy principle [33, 34]. This type of inference identifies the least biased model consistent with the given information. Due to insufficient data to study higher-order epistasis, epistatic landscape models are often fitted up to pairwise interactions (e.g. [35]) and shown to be able to capture a substantial fraction of the observed variance. In particular, the direct-coupling analysis (DCA) [36, 37] uses homologous protein sequences as the only input to deduce single-residue counts and covariance information, inferring an effective Hamiltonian (a binding energy landscape) characterized by local biases (fields) and pairwise couplings. This coevolutionary structural analysis performs remarkably well for predicting direct residue contacts in proteins. By contrast, the combinatorial completeness of phenotypic measurements in our experiment allows us to examine to what extent higher-order interactions contribute to folding stability (a highly non-local property compared to inter-residue binding). It turns out, as shown below, that going beyond pairwise epistasis is essential for revealing the counterintuitive role of the epistatic hotspot in structuring evolutionary constraints, such that path-orienting ruggedness facilitates landscape navigation.

To first determine the statistical significance of higher order epistasis, we plot in Fig. S9A the distribution of the triplet coefficients K_{ijk} and that of the pairwise coefficients J_{ij}, both inferred using the full (third-order) specific epistasis model (Eq. 2) validated by distinct methods. Out of the total contribution, we delineate the portion that includes the hotspot (site 53) among the triplet or pair of sites (shaded area). We noticed two trends. First, hotspot-dependent coefficients span the full range of values for both K_{ijk} and J_{ij}. Second, strong positive K_{ijk} values are exclusively associated with the hotspot, indicative of large fitness gain upon hotspot mutation. To make the effect more apparent, we made a scatter plot (Fig. S9B) showing the inferred fitness values $F(\mathbf{s})$ of individual genotypes $\mathbf{s}$ with the third-order terms being included (y-axis) vs. excluded (x-axis). When the hotspot is in the wild-type state ($s_{i=53} = 0$), fitness values of the second-order and third-order models are well correlated (blue points lying around the diagonal). However, such correlation is lost when the hotspot is mutated ($s_{i=53} = 1$) and the deviation from the diagonal reflects the strong positive fitness effect from hotspot-dependent triplet interactions (orange points).

To evaluate the role of higher-order epistasis in the paradoxical effect that hotspot mutation exerts on ruggedness and accessibility, we made the corresponding plots using the second-order model (Figs. S9C-D) to be compared with the behavior of the full model shown in Figs. 3A-B in the main text. The contrast is clear: without triplet interactions, hotspot mutation only modestly increases the level of ruggedness in the 9-site sublandscape (a smaller γ) without changing the number of fitness optima (Fig. S9C). Moreover, the accessibility to $F_{\max}$ starting from the germline, measured by the absorbing probability, is now reduced, rather than enhanced, as the hotspot is mutated (blue to orange line, Fig. S9D right). Therefore, hotspot-associated higher-order epistasis is responsible for creating the constructive ruggedness that orients a population to the productive direction of smooth fitness increase leading toward $F_{\max}$.

D. COMPARISON OF LANDSCAPE TOPOGRAPHY VIA LOW-DIMENSIONAL VISUALIZATION

Methods of dimensionality reduction allow to visualize a high-dimensional fitness landscape in a low-dimensional embedding. Different methods place emphasis on different aspects of the genotype-fitness map.

1. Force-directed graph embedding

Topography of a high-dimensional fitness surface can be visualized using force-directed graph layout [38], a method for low-dimensional network embedding where nodes connected by high-weight edges tend to be placed nearby. Aiming for a representation in which genotypes with similar fitness values are in proximity whereas genotypes with large fitness disparity are far apart, we interpret the nodes of the network as genotypes and connect pairs of genotypes ($\mathbf{s}$, $\mathbf{s}'$) according to the following weight:

$$w(\mathbf{s}, \mathbf{s}') = \frac{1}{0.001 + |F(\mathbf{s}) - F(\mathbf{s}')|}, \tag{15}$$

such that similar fitness entails strong connection. The regularization term in the denominator removes singularity due to effective neutrality when $|F(\mathbf{s}) - F(\mathbf{s}')|$ is smaller than the inverse of the population size.

Roughly speaking, the low-dimensional embedding can be viewed as the ground-state node configuration that minimizes the total potential energy distributed across the network edges, which act like springs with force constants $w(\mathbf{s}, \mathbf{s}')$. Note that constraining to a low-dimensional embedding space implies that not all springs can be simultaneously relaxed.

The remaining choice is in regard to which pairs of nodes (or genotypes) should be connected. When we only connect one-mutant neighbors ($d_{\mathrm{H}}(\mathbf{s}, \mathbf{s}') = 1$) to individual nodes, like in previous work [38], the embedding exhibits multiple genotype clusters, where intra-cluster genotypes are close in fitness values while inter-cluster gaps reflect fitness jumps (Fig. 1D and Fig. S4B). A large fitness jump is often associated with mutation of an epistatically important site; see the network embedding colored by the mutational state of site 53 in Fig. S4B.

As we extend the connectivity to next-nearest neighbors ($d_{\mathrm{H}}(\mathbf{s}, \mathbf{s}') \leq 2$), the additional constraints introduced cause coalescence of genotype clusters. Notably, two distinct large clusters remain and they are distinguished by the mutational state of the epistatic hotspot (Fig. S4A, blue vs orange). This observation stresses the heterogeneity of landscape ruggedness by revealing a hierarchy of epistatic importance among sequence sites.

We use Python's `igraph` package (function `layout_drl` for distributed recursive layout with default parameters) to represent in two dimensions the topography of the full antibody landscape and its sublandscapes.

2. t-distributed stochastic neighbor embedding (t-SNE)

To visualize the mutational paths determined from simulations of Wright-Fisher dynamics in well-mixed and structured populations, we use t-SNE, a standard method of dimensionality reduction, where Hamming distance serves as a natural metric of genotypic proximity.

We use Python's `sklearn` package (function `sklearn.manifold.TSNE`) to perform sequence-space embedding in two dimensions.

3. Comparison of landscape embedding

Both force-directed graph embedding and t-SNE are inherently stochastic: for a given landscape, the 2D coordinates of genotypes vary from one execution to another despite identical parameters. In addition, both methods are invariant under translation, rotation, and reflection in the embedding space.

In order to compare landscapes of equal size, we use an identical seed for the random number generator via the `seed` argument of `layout_drl` or the `random_state` argument of `TSNE`. Subsequently, we infer and apply a rotation-translation operation to one of the two embedding attempts to maximize their overlap. As a result, any remaining mismatch would reflect actual differences in landscape topography (see contrasting examples in Fig. 1E between mutating a weakly epistatic site and mutating an epistatic hotspot).

Specifically, given the 2D coordinates $\mathbf{x}$ and $\mathbf{y}$ of genotype $\mathbf{s}$ in two landscapes, we transform $\mathbf{x}$ according to $\mathbf{x} \leftarrow \mathbf{R}\mathbf{x}+\mathbf{t}$, while leaving $\mathbf{y}$ unchanged. Here, $\mathbf{R}$ and $\mathbf{t}$ denote respectively a 2×2 rotation matrix and a 2×1 translation vector. They are obtained through a singular value decomposition (SVD) of the 2×2 matrix $\mathbf{H} = (\mathbf{x}-\langle\mathbf{x}\rangle)(\mathbf{y}-\langle\mathbf{y}\rangle)^{\mathrm{T}}$, where $\langle\mathbf{x}\rangle$ and $\langle\mathbf{y}\rangle$ are the mean coordinates: Writing $\mathbf{H} = \mathbf{U}\mathbf{S}\mathbf{V}^T$, where $\mathbf{U}$ and $\mathbf{V}$ are orthogonal matrices and $\mathbf{S}$ is diagonal, we take $\mathbf{R} = \mathbf{V}\mathbf{U}^T$ and $\mathbf{t} = \langle\mathbf{y}\rangle - \mathbf{R}\langle\mathbf{x}\rangle$. To account for possible reflection, we take $\mathbf{R} \leftarrow \mathbf{M}\mathbf{R}$ with $\mathbf{M} = \begin{pmatrix} 1 & 0 \\ 0 & -1 \end{pmatrix}$ if $\det(\mathbf{R}) < 0$.

E. GLOBAL AND LOCAL MEASURES OF LANDSCAPE RUGGEDNESS

1. Fitness correlation

One intuitive metric of landscape ruggedness considers how fitness correlation between genotypes depends on their genetic distance. That is, it measures how quickly fitness values decorrelate as mutations accumulate; a faster decay indicates stronger ruggedness. As a global measure of landscape ruggedness, it is defined as the average correlation of fitness values between all pairs of genotypes d mutations apart:

$$\rho(d) = \frac{\langle F(\mathbf{s})F(\mathbf{s}_{[i_1,\ldots,i_d]})\rangle}{\langle F(\mathbf{s})^2\rangle} = \frac{\sum_{\mathbf{s}}\sum_{i_1=d}^{L}\sum_{i_2=1}^{i_1-1}\cdots\sum_{i_d=1}^{i_{d-1}-1} F(\mathbf{s})F(\mathbf{s}_{[i_1\ldots i_d]})}{\binom{L}{d}\sum_{\mathbf{s}} F(\mathbf{s})^2}, \tag{16}$$

where $\mathbf{s}_{[\ldots]}$ denotes the genotype that is mutated at all sites listed in the bracket relative to the reference genotype $\mathbf{s}$, and the average indicated with $\langle\cdot\rangle$ runs through 2^L choices of the reference genotype $\mathbf{s}$ and $\binom{L}{d}$ choices of d out of L sites.

Since the fitness correlation function $\rho(d)$ measures correlations on the genotype level, it masks effects of epistatic inequality among sequence loci that we seek to detect. Thus, we need a ruggedness metric that permits locality.

2. Correlation of fitness effects of mutations: γ statistics

The γ measure [39] allows a bottom-up characterization of landscape ruggedness at the level of sequence loci. It considers correlations in fitness effects of mutations $\Delta_i(\mathbf{s}) = F(\mathbf{s}_{[i]}) - F(\mathbf{s})$ (rather than in $F(\mathbf{s})$ itself) across sequence contexts in which they may arise.

In its most local form, $\gamma_{i\to j}$ quantifies how fitness effect of a mutation j is affected by the presence of mutation i. More precisely, it is defined as the average correlation of the fitness effects of mutation j in the absence ($\Delta_j(\mathbf{s})$) and presence ($\Delta_j(\mathbf{s}_{[i]})$) of mutation i across all possible choices of the reference sequence $\mathbf{s}$

$$\gamma_{i\to j} = \frac{\langle\Delta_j(\mathbf{s})\Delta_j(\mathbf{s}_{[i]})\rangle}{\langle\Delta_j(\mathbf{s})^2\rangle} = \frac{\sum_{\mathbf{s}}\Delta_j(\mathbf{s})\Delta_j(\mathbf{s}_{[i]})}{\sum_{\mathbf{s}}\Delta_j(\mathbf{s})^2}. \tag{17}$$

Note that $\langle \Delta_j(\mathbf{s}) \rangle = 0$. The generally asymmetric matrix $\gamma_{i\to j}$ represents a directed map of epistatic coupling between sequence loci, indicating the sign, magnitude and directionality of such effects.

The summary statistics $\gamma_{i\to\cdot}$ ($\gamma_{\cdot\to j}$) measure the overall effect of "outgoing" ("incoming") epistasis induced by a mutation i on any other possible mutation (by any possible background mutation on mutation j):

$$\gamma_{i\to\cdot} = \frac{\langle \Delta_\cdot(\mathbf{s})\Delta_\cdot(\mathbf{s}_{[i]})\rangle}{\langle \Delta_\cdot(\mathbf{s})^2\rangle} = \frac{\sum_{\mathbf{s}} \sum_{\substack{j=1\\ j\neq i}}^{L} \Delta_j(\mathbf{s})\Delta_j(\mathbf{s}_{[i]})}{\sum_{\mathbf{s}} \sum_{\substack{j=1\\ j\neq i}}^{L} \Delta_j(\mathbf{s})^2}, \tag{18}$$

$$\gamma_{\cdot\to j} = \frac{\langle \Delta_j(\mathbf{s})\Delta_j(\mathbf{s}_{[\cdot]})\rangle}{\langle \Delta_j(\mathbf{s})^2\rangle} = \frac{\sum_{\mathbf{s}} \sum_{\substack{i=1\\ i\neq j}}^{L} \Delta_j(\mathbf{s})\Delta_j(\mathbf{s}_{[i]})}{(L-1)\sum_{\mathbf{s}} \Delta_j(\mathbf{s})^2}, \tag{19}$$

where the average $\langle\cdot\rangle$ runs over 2^L reference sequences $\mathbf{s}$ and $(L-1)$ background mutations.

One can further generalize it to a global form $\gamma(d)$, which is related to $\rho(d)$ through $\gamma(d) = \frac{\rho(d+1)-\rho(d)}{1-\rho(d)}$ [39]. Accordingly, we have

$$\gamma(1) \equiv \gamma_{\cdot\to\cdot} = \frac{\langle \Delta_\cdot(\mathbf{s})\Delta_\cdot(\mathbf{s}_{[\cdot]})\rangle}{\langle \Delta_\cdot(\mathbf{s})^2\rangle} = \frac{\sum_{\mathbf{s}} \sum_{i=1}^{L} \sum_{\substack{j=1\\ j\neq i}}^{L} \Delta_j(\mathbf{s})\Delta_j(\mathbf{s}_{[i]})}{(L-1)\sum_{\mathbf{s}}\sum_{j=1}^{L} \Delta_j(\mathbf{s})^2}. \tag{20}$$

Then, how fitness effects decorrelate as background mutations accumulate on d distinct sites $i_1,\ldots,i_d$ other than site j is given by

$$\gamma(d) = \frac{\langle \Delta_\cdot(\mathbf{s})\Delta_\cdot(\mathbf{s}_{[\cdot,\ldots,\cdot]})\rangle}{\langle \Delta_\cdot(\mathbf{s})^2\rangle} = \frac{\sum_{\mathbf{s}} \sum_{i_1=d}^{L} \sum_{i_2=1}^{i_1-1}\cdots\sum_{i_d=1}^{i_{d-1}-1} \sum_{\substack{j=1\\ j\neq i_1,\ldots,i_d}}^{L} \Delta_j(\mathbf{s})\Delta_j(\mathbf{s}_{[i_1\ldots i_d]})}{\binom{L-1}{d}\sum_{\mathbf{s}}\sum_{j=1}^{L} \Delta_j(\mathbf{s})^2}. \tag{21}$$

As a correlation measure, γ is bounded between -1 and $+1$. $\gamma \sim 1$ indicates high correlations of fitness effects of mutations regardless of the sequence context (i.e. an additive fitness landscape). $\gamma \sim 0$ indicates a lack of correlation when averaged over the genetic background, reflecting the presence of sign epistasis. Whereas $\gamma \sim -1$ indicates anticorrelation of fitness effects and signals reciprocal sign epistasis.

F. SUBLANDSCAPE

To probe heterogeneity of landscape ruggedness, we construct sublandscapes of varying size and location by pinning a subset of sequence loci to their wild-type or mutated state.

1. Definition

We obtain a sublandscape of $(L-n)$ dimensions from the L-dimensional global landscape by pinning n sequence sites (with $n \le L-2$); it thus hosts 2^{L-n} genotypes. We denote by $\mathbb{B}_{i_1,\ldots,i_n}^{s_{i_1},\ldots,s_{i_n}}$ an $(L-n)$-site sublandscape obtained by pinning sites $i_1,\ldots,i_n \in \{1,2,\ldots,L\}$ to states $s_{i_1},\ldots,s_{i_n} \in \{0,1\}$, respectively. For instance, $\mathbb{B}$ denotes the L-site global landscape and $\mathbb{B}_{53}^{1}$ represents an $(L-1)$-site sublandscape in which site 53 is pinned to its mutated state. For a given n, there are in total $2^n\binom{L}{n}$ sublandscapes of $(L-n)$ dimensions, a product of the $\binom{L}{n}$ possible positions of the n pinned sites and their 2^n possible configurations.

2. Heterogeneity of ruggedness

Homogeneity of landscape ruggedness stems from equal epistatic involvement of all sequence loci, an assumption implicitly made in most theoretical models. As a consequence, ruggedness would be statistically equivalent across sublandscapes of different sizes and at different locations.

Mathematically, we quantify heterogeneity of ruggedness across the sequence space using a generalized γ measure. For a particular sublandscape with n pinned sites, $\mathbb{B}_{i_1,\ldots,i_n}^{s_{i_1},\ldots,s_{i_n}}$, we can define the correlation of fitness effects of mutations

for pairs of genotypes d mutations apart (with $0 \le d \le L-n-1$):

$$\gamma^{s_{i_1},\ldots,s_{i_n}}_{i_1,\ldots,i_n}(d) = \frac{\langle \Delta_\cdot(\mathbf{s})\Delta_\cdot(\mathbf{s}_{[\cdot,\ldots,\cdot]})\rangle}{\langle \Delta_\cdot(\mathbf{s})^2\rangle} = \frac{\sum_{\mathbf{s}\in\mathbb{B}^{s_{i_1},\ldots,s_{i_n}}_{i_1,\ldots,i_n}} \sum^{L}_{\substack{i_{n+1}=1\\ i\neq i_1,\ldots,i_n}} \cdots \sum^{L}_{\substack{i_{n+d}=1\\ i\neq i_1,\ldots,i_{n+d-1}}} \sum^{L}_{\substack{j=1\\ j\neq i_1,\ldots,i_{n+d}}} \Delta_j(\mathbf{s})\Delta_j(\mathbf{s}_{[i_{n+1},\ldots,i_{n+d}]})}{\binom{L-n-1}{d}\sum_{\mathbf{s}\in\mathbb{B}^{s_{i_1},\ldots,s_{i_n}}_{i_1,\ldots,i_n}} \sum^{L}_{\substack{j=1\\ j\neq i_1,\ldots,i_n}} \Delta_j(\mathbf{s})^2} \tag{22}$$

where $\langle\cdot\rangle$ denotes an average over $\mathbf{s}\in\mathbb{B}^{s_{i_1},\ldots,s_{i_n}}_{i_1,\ldots,i_n}$. In particular, we have

$$\gamma^{s_{i_1},\ldots,s_{i_n}}_{i_1,\ldots,i_n}(1) = \frac{\langle \Delta_\cdot(\mathbf{s})\Delta_\cdot(\mathbf{s}_{[\cdot]})\rangle}{\langle \Delta_\cdot(\mathbf{s})^2\rangle} = \frac{\sum_{\mathbf{s}\in\mathbb{B}^{s_{i_1},\ldots,s_{i_n}}_{i_1,\ldots,i_n}} \sum^{L}_{\substack{i_{n+1}=1\\ i\neq i_1,\ldots,i_n}} \sum^{L}_{\substack{j=1\\ j\neq i_1,\ldots,i_{n+1}}} \Delta_j(\mathbf{s})\Delta_j(\mathbf{s}_{[i_{n+1}]})}{(L-n-1)\sum_{\mathbf{s}\in\mathbb{B}^{s_{i_1},\ldots,s_{i_n}}_{i_1,\ldots,i_n}} \sum^{L}_{\substack{j=1\\ j\neq i_1,\ldots,i_n}} \Delta_j(\mathbf{s})^2}. \tag{23}$$

To study the distribution of ruggedness values over $\binom{L}{n}$ subspace locations for a given n, we average over the 2^n configurations of the pinned sites to obtain

$$\gamma(n,d) \equiv \gamma_{i_1,\ldots,i_n}(d) = 2^{-n} \sum_{s_{i_1},\ldots,s_{i_n}=0,1} \gamma^{s_{i_1},\ldots,s_{i_n}}_{i_1,\ldots,i_n}(d). \tag{24}$$

To reveal heterogeneity of landscape ruggedness, we compare the distribution of $\gamma(n,1)$ of the antibody landscape to those of the NK model with modest K values, with the latter serving as a null model with homogeneous ruggedness.

G. SIMULATING EVOLUTION IN EMPIRICAL AND MODEL LANDSCAPES

We use different dynamics, namely Markov Chain Monte Carlo dynamics and Wright-Fisher dynamics, to simulate evolutionary trajectories leading from the germline genotype $\mathbf{s}_0$ to the global fitness maximum $\mathbf{s}_{F_{\max}}$, both in the antibody landscape and in NK landscapes with similar levels of ruggedness. A simple spatial structure is employed to reveal how real-space constraints affect sequence-space exploration by finite populations.

1. Evolutionary dynamics

I. Markov Chain Monte Carlo (MCMC) dynamics (strong-selection weak-mutation limit)

In the strong-selection weak-mutation (SSWM) limit, mutation occurs on much slower timescales than selection. As a consequence, a population occupies a single genotype at any given time and mutations are acquired in a series of fitness-increasing steps, which constitute an adaptive walk. Evolutionary dynamics thus simplifies to a Markov chain along which a population transitions from its current genotype to a one-mutant neighbor at each step, with a probability that only depends on the fitness effect of the associated point mutation. The walk ends upon arrival at a fitness optimum (an absorbing state). This simplification due to a separation of timescales greatly reduces the computational complexity of evolutionary simulations, allowing an efficient quantification of $F_{\max}$ accessibility and path entropy (see below): one can simulate evolutionary dynamics by sampling the mutational steps according to a constant $2^L \times 2^L$ transition matrix $\mathbf{P} = (P_{\mathbf{ss}'})$, whose entries specify the probability of evolving from any given genotype $\mathbf{s}$ to any mutant genotype $\mathbf{s}'$ [38, 40].

Specifically, the matrix elements are given by

$$P_{\mathbf{ss}'} = \begin{cases} \dfrac{p(\mathbf{s}'|\mathbf{s})}{\sum_{\sigma \text{ s.t. } d_{\rm H}(\mathbf{s},\sigma)=1} p(\sigma|\mathbf{s})}, & \text{if } \mathbf{s}\notin \mathbf{S}_{\max} \text{ and } d_{\rm H}(\mathbf{s},\mathbf{s}')=1,\\ 0, & \text{if } \mathbf{s}\notin \mathbf{S}_{\max} \text{ and } d_{\rm H}(\mathbf{s},\mathbf{s}')\neq 1,\\ \delta_{\mathbf{ss}'}, & \text{if } \mathbf{s}\in \mathbf{S}_{\max}, \end{cases} \tag{25}$$

which satisfy the normalization conditions $\sum_{\mathbf{s}'} P_{\mathbf{ss}'} = 1$ for a stochastic matrix. Here $\mathbf{S}_{\max}$ denotes the set of fitness optima, which act as in total $|\mathbf{S}_{\max}|$ absorbing states. The remaining $2^L - |\mathbf{S}_{\max}|$ genotypes represent transient states on the L-dimensional hypercube. The condition that only single-mutants can arise from a transient (non-absorbing) genotype $\mathbf{s}$ is enforced through $d_{\rm H}(\mathbf{s},\mathbf{s}')=1$, where $d_{\rm H}(\mathbf{s},\mathbf{s}')$ denotes the Hamming distance (the total number of pairwise allelic differences across L loci) between genotypes $\mathbf{s}$ and $\mathbf{s}'$.

The first line of Eq. (25) describes transitions from a transient state $\mathbf{s}$ to any of its 1-mutant neighbors $\mathbf{s}'$. The transition probability is given by the fixation probability of a given point mutation normalized by the total fixation probability of all single mutants σ of genotype $\mathbf{s}$. The fixation probability of a mutation with fitness effect $\Delta_{\mathbf{ss}'} = F(\mathbf{s}') - F(\mathbf{s})$ is given by $p(\mathbf{s}'|\mathbf{s}) = \frac{1-\exp(-2\Delta_{\mathbf{ss}'})}{1-\exp(-2N_{\text{pop}}\Delta_{\mathbf{ss}'})}$, where N_{pop} denotes the (effective) population size. Under strong selection ($N_{\text{pop}}\Delta_{\mathbf{ss}'} \gg 1$), fitness-decreasing mutations are forbidden, and the fixation probability becomes $p(\mathbf{s}'|\mathbf{s}) = \max(0, 1-\exp(-2\Delta_{\mathbf{ss}'}))$. In the weak-mutation limit, occurrence of multiple mutations before any reaches fixation is negligible, hence the second line of Eq. (25). The third line defines the absorbing states.

One can represent the transition matrix $\mathbf{P}$ as a block matrix by permutating rows and columns:

$$\mathbf{P} = \begin{pmatrix} \mathbf{Q} & \mathbf{R} \\ \mathbf{0} & \mathbb{1} \end{pmatrix} \tag{26}$$

where $\mathbf{Q}$ is a $(2^L - |\mathbf{S}_{\max}|) \times (2^L - |\mathbf{S}_{\max}|)$ matrix connecting transient states and $\mathbf{R}$ is a $(2^L - |\mathbf{S}_{\max}|) \times |\mathbf{S}_{\max}|$ matrix providing the transition probability from any transient state to any absorbing state. The $|\mathbf{S}_{\max}| \times (2^L - |\mathbf{S}_{\max}|)$ zero matrix forbids any transition from an absorbing state to a transient state and the $|\mathbf{S}_{\max}| \times |\mathbf{S}_{\max}|$ identity matrix ensures trapping at absorbing states.

II. Wright-Fisher dynamics and spatial population structure

Wright-Fisher dynamics represents a more realistic evolutionary scheme: mutation and selection can occur on similar timescales, which in turn permits clonal interference between coexisting lineages (provided a considerable mutation rate in a large population) [41]. It also allows one to conveniently study the interplay of constraints on real-space exploration (e.g. spatial scale of selection) and those on evolutionary navigation (e.g. epistasis).

Inspired by [42], we implement a simple spatial structure by placing a population of size N_{pop} on a one-dimensional lattice with N_{pop} nodes and applying periodic boundary conditions (i.e. forming a ring of N_{pop} nodes with one individual per node). Starting with a monomorphic population with all individuals carrying the same initial genotype $\mathbf{s}^0$ at time $t = 0$, the population state advances by iterating between two steps: (i) selection and migration and (ii) mutation. (i) Denote by $\mathbf{s}_i^{t+1}$ the genotype of the individual occupying node i at time $(t+1)$. This individual inherits a parental genotype $\mathbf{s}_j^t$ among $(2r+1)$ candidates present in its r-neighborhood ($j \in [i-r, i+r]$) at time t with the following probability

$$P(\mathbf{s}_i^{t+1} = \mathbf{s}_j^t) = \frac{\exp\big(F(\mathbf{s}_j^t)\big)}{\sum_{k=i-r}^{i+r} \exp(F(\mathbf{s}_k^t))}. \tag{27}$$

This probability depicts selection and migration under spatial constraint: an individual bearing a fitter genotype is more likely to produce offspring which then disperse into a finite neighborhood. The size r of the neighborhood thus controls the spatial range of competition and dispersal in one generation. Moreover, it sets the maximum distance a mutation can spread in one generation. Spatial constraint is stronger for smaller r and can be removed by setting $r = N_{\text{pop}}$ (i.e. well-mixed). (ii) A lattice sweep of mutation follows: each sequence locus of each individual is mutated (bit-flipped) with a probability μ. In general, spatial structure may slow fixation and help maintain genetic diversity which are likely beneficial to evolution in rugged fitness landscapes [43].

In each simulation, two steps are iterated for $t_{\max} = 2000$ time steps. We performed $M = 10^4$ simulations in the antibody landscape and 2500 simulations across 50 NK landscapes (50 simulations per landscape) for $K = 0$ and $K = 2$, with and without spatial structure. Parameters used in the simulations are: $N_{\text{pop}} = 500$, $\mu = 0.001$, $r = 2$ for a structured population and $r = N_{\text{pop}}$ for a well-mixed population.

2. Evolutionary paths

I. Path identity and path weight

There are different ways to define evolutionary paths taken by an adapting population in a genotypic fitness landscape. Here, we follow the definition of "lines of descent" [41] which represent the lineages arriving at the target genotype at the earliest time. This definition associates a unique path π to each successful population that discovers $\mathbf{s}_{F_{\max}}$ within a given time starting from the germline $\mathbf{s}_0$.

A path of length l can be viewed, equivalently, as an ordered set of $l+1$ visited genotypes $\pi = (\mathbf{s}_0, \mathbf{s}_1, \mathbf{s}_2, \ldots, \mathbf{s}_\ell)$, or as a particular permutation of l mutations $\pi = (\pi_1, \pi_2, \ldots, \pi_\ell)$. These paths are obtained by tracing from the "leaf"

back to the "root" of the lineage tree that is recorded in the forward simulations (MCMC and Wright-Fisher alike). Within a fixed time, the target genotype is reached. The last step of the path is determined as the connection between the target and the genotype from which it arose by mutation. Subsequent steps are constructed in an analogous way, where we search for the genotype from which the latest ancestor arose before giving rise to the next genotype. This procedure is repeated until the root of the tree (i.e. starting point of the simulation) is reached.

The paths thus generated do not include all the paths explored by the population, nor all the paths contributing to the production of the final population. They, instead, correspond to "stepwise fastest" paths by which the target genotype may have been discovered. The assumption is that this path plays a dominant role in creating the target genotype. If multiple paths have considerable probabilities for being stepwise fastest, they should all show up among repeated simulations. This leads to the concept of path weight w_π, defined as the probability that a particular path π is taken by an adapting population. It can be estimated from replicate simulations through

$$w_\pi = \frac{M_\pi}{M} = \frac{1}{M}\sum_{m=1}^{M} \delta_{\pi^m,\pi}. \tag{28}$$

Here M is the total number of independent simulations and M_π the number of populations that take path π. π_m labels the path taken in the m-th simulation. Indeed, in the vast majority of populations, a single path dominates success. Only very rarely, F_{max} is discovered multiple times by distinct lineages within a population. When this occurs, we retain the "first arrival" path.

Path weight can also be defined separately for successful and failure paths (indicated by superscripts $^+$ and $^-$, respectively), which both reach F_{max} within the given time but the former succeed in enriching it to the occupancy threshold while the latter fail to do so:

	success only	failure only	overall
number of populations	M^+	M^-	$M = M^+ + M^-$
path weight	$w_\pi^+ = \frac{M_\pi^+}{M^+}$	$w_\pi^- = \frac{M_\pi^-}{M^-}$	$w_\pi = \frac{M_\pi}{M} = \frac{M_\pi^+ + M_\pi^-}{M^+ + M^-}$

We visualize the unique paths identified in t-SNE embedding (Figs. 5A and 6D, Fig. S7C), where edges (mutations) connect adjacent nodes (consecutively visited genotypes). The spectrum of path weight differs between well-mixed and structured populations (Fig. S7A, left panel).

II. Path entropy

To assess the impact of hotspot mutation on path repeatability, we calculate from a simulated path ensemble the Gibbs-Shannon entropy [41]

$$S_\pi = -\frac{1}{\ln(\ell!)}\sum_\pi w_\pi \ln(w_\pi). \tag{29}$$

Here the sum runs over $\ell!$ possible direct paths π of length ℓ. We scale the path entropy by its maximum value $\ln(\ell!)$ which is attained when all possible paths are equally likely. This scaled path entropy allows comparison between the full landscape and the sublandscapes (Fig. 4B). To enforce direct paths, we inhibit reverse mutations via the transition matrix.

With a finite number of independent simulations (M in total), paths with a frequency lower than $1/M$ typically will not be observed, leading to a systematic underestimation of the path entropy. We thus use the computationally efficient MCMC simulations to enhance path sampling. We run $M = 10^6$ simulations for the antibody landscape (full landscape and each sublandscape) and $M = 10^5$ simulations for each of the 50 realizations of the NK landscape at each K value. For the latter, the values of path entropy are computed for each landscape realization (at a given K) and then averaged across realizations.

III. Mutational order

Epistasis constrains the availability of viable paths, such that only a subset of all possible permutations of mutations is observed. To evaluate the strength of constraint on the order of occurrence among the 8 mutations required to

reach $F_{\max}$ from the germline, we compute the probability that mutation i occurs ahead of mutation j along successful paths, $P[t(i) < t(j)]$. It satisfies the condition $P[t(i) < t(j)] + P[t(j) < t(i)] = 1$ under the assumption that mutations are acquired one by one. We estimate this probability empirically from M successful populations:

$$P[t(i) < t(j)] = \frac{1}{M} \sum_{m=1}^{M} \mathbb{1}\left(t(i) < t(j) : \pi^m_{t(i)} = i \wedge \pi^m_{t(j)} = j\right), \tag{30}$$

where $\mathbb{1}(\cdot)$ denotes the indicator function set to 1 if the argument is true and to 0 otherwise. A value close to 0 or 1 indicates strong epistatic constraint on pairwise ordering, whereas a value close to 1/2 implies vanishing constraint. In the extreme case of maximum path entropy, $P[t(i) < t(j)] \sim 1/2$ for all pairs of mutations.

3. Effectiveness of landscape navigation

Evolutionary accessibility of a target genotype depends both on the availability of viable paths and on the chance by which each path can be realized by an evolving population. While the former is primarily set by the genotype-fitness map, the latter is furthermore influenced by the dynamics of landscape navigation driven by evolutionary forces (genetic drift and migration in addition to mutation and selection). We thus use two measures to quantify the effectiveness of landscape navigation: (1) absorbing probability of the global fitness optimum in the SSWM limit and (2) success rate of enriching the fittest genotype under Wright-Fisher dynamics. The first measure emphasizes the importance of landscape topography whereas the second focuses on the paths taken to access the target.

I. Absorbing probability of the global fitness optimum

The absorbing probability of $F_{\max}$ (starting from one of $2^L - |\mathbf{S}_{\max}|$ non-absorbing states) in the SSWM limit quantifies the extent to which the fitness landscape supports an adaptive walk to $F_{\max}$ with no encounter of any local optimum. Mathematically, the absorbing probability $A_{\mathbf{s}_0\mathbf{s}_{F_{\max}}}$ of the global optimum $\mathbf{s}_{F_{\max}}$ starting from $\mathbf{s}_0$ is given by the probability that an MCMC trajectory reaches $\mathbf{s}_{F_{\max}}$ before encountering any other absorbing state. Using the block representation of the transition matrix $\mathbf{P}$ (Eq. (26)), Markov chain theory [40] yields

$$A_{\mathbf{s}_0\mathbf{s}_{F_{\max}}} = (\mathbf{NR})_{\mathbf{s}_0\mathbf{s}_{F_{\max}}} = \sum_{\mathbf{s}} N_{\mathbf{s}_0\mathbf{s}} R_{\mathbf{s}\mathbf{s}_{F_{\max}}}. \tag{31}$$

Here $\mathbf{N}$ is the fundamental matrix of $\mathbf{Q}$, namely $\mathbf{N} = \sum_{t=0}^{\infty} \mathbf{Q}^t = (\mathbb{1} - \mathbf{Q})^{-1}$, where the last expression performs matrix inverse. Recall that the $\mathbf{Q}$ matrix connects transient (non-absorbing) states and the $\mathbf{R}$ matrix provides the transition probability from any transient state to any absorbing state. Eq. (31) has an intuitive interpretation as the likelihood that an adaptive walk does not reach the target $\mathbf{s}_{F_{\max}}$ for t iterations and finds it at iteration $t+1$. A sum over t is taken via matrix $\mathbf{N}$. This formulation is used to generate the histograms in Fig. 3B and Fig. S5C, revealing the impact of epistatic inequality on landscape topography hence navigation.

II. Success rate of enriching the fittest genotype

Under Wright-Fisher dynamics of finite populations, genetic drift and fitness valley crossing become possible. We now characterize the navigation performance through the success rate by which a population first enriches $F_{\max}$ to an occupancy threshold. We find that an intermediate value of the threshold (around 0.5) works best, because a low threshold is easily reached by local optima whereas a high threshold is rarely achieved by any genotype.

We estimate the success rate from M replicate simulations of Wright-Fisher dynamics with an identical initial condition (all N_{pop} individuals carrying the germline genotype):

$$A_{\mathbf{s}_0\mathbf{s}_{F_{\max}}} = \frac{1}{M} \sum_{m=1}^{M} \delta_{r_m,1}, \tag{32}$$

where r_m is the rank of the fitness optimum whose occupancy first exceeds the threshold in the m-th replicate population.

For the antibody landscape, we simulate $M = 10^4$ replicate populations for $t_{\max} = 2000$ generations, with and without spatial structure. For the NK landscape, we draw 50 independent realizations of the landscape for $K = 0$ and $K = 2$, respectively. We only retain realizations that exhibit the same number of fitness optima and the same mutational distance between $\mathbf{s}_0$ and $\mathbf{s}_{F_{\max}}$ as the antibody landscape. In this way, antibody landscape and constrained NK landscapes only differ in the location and height of fitness optima, while having the same level of ruggedness and the same path length. For each NK landscape, we simulate 50 replicate populations. Hence, $M = 2500$ trajectories are generated for each value of K, with or without spatial structure.

III. Net gain in success rate due to spatial structure

To understand the overall trend of difference in success rate between well-mixed and structured populations (black and red curves in Fig. 6A), we consider the contrast in the dominant path category and the failure mode in the absence and presence of spatial structure. In particular, we account for two opposing contributions to structure-induced excess accessibility:

$$\Delta A_{\mathbf{s}_0\mathbf{s}_{F_{\max}}} = \frac{1}{M}\left(\sum_{\substack{\pi \text{ crossing} \\ w^+_{\pi,r=2}>0}} w^-_{\pi,r=N_{\text{pop}}} - \sum_{\substack{\pi \text{ bypassing} \\ w^+_{\pi,r=N_{\text{pop}}}>0}} w^-_{\pi,r=2} \right). \tag{33}$$

The first sum represents the total weight of direct paths taken that are successful with structure ($w^+_{\pi,r=2} > 0$) but fail without structure ($w^-_{\pi,r=N_{\text{pop}}} > 0$). This gain in success due to structure is attributable to stepping-stone crossing paths. The second term represents the excess failure with structure due to road-block bypassing paths that succeed without structure ($w^+_{\pi,r=N_{\text{pop}}} > 0$) but fail with structure ($w^-_{\pi,r=2} > 0$). The difference between the two terms (solid red curve in Fig. S7B) indeed captures the overall dependence of net success on occupancy threshold (gray curve in Fig. S7B). At intermediate occupancy thresholds, spatial structure most strongly boosts success rate and thus enhance evolvability of $F_{\max}$.

We implement Eq. (33) based on the workflow outlined by the following pseudocode:

```
# assuming list of M populations for both without and with structure:
# pops[flag_structure]

# assuming list of M paths corresponding to the M populations:
# paths[flag_structure]

# assuming function to check if a population is a success or failure,
# given a frequency threshold for success (e.g.~f_thr = 0.5):
# function is_success(population, f_thr=0.5) -> failure, success

# assuming function to check if a path is crossing through a genotype of interest:
# function is_crossing(path) -> false, true

# iterate over without/with spatial structure
for flag_structure in [without, with]:
    # iterate over failure/success
    for flag_outcome in [failure, success]:
        # initialize all path weights with zero
        for path in union(set(paths[without]), set(paths[with])):
            path2weight[flag_structure,flag_outcome][path] = 0

    # iteration over all populations and their paths
    for pop, path in zip(pops[flag_structure], paths[flag_structure]):
        # determine if the population is a success or failure
        flag_outcome = is_success(pop)
        # increment the corresponding path weight
        path2weight[flag_structure,flag_outcome][path] += 1
```

```
# initialize counter for crossing and bypassing paths
cnt_c, cnt_b = 0, 0

# iterate over paths with success with spatial structure
for path in path2weight[with,success]:
    # check if the path is crossing
    if is_crossing(path):
        # increment the counter
        cnt_c += path2weight[without,failure][path]

# iterate over paths with success without spatial structure
for path in path2weight[without,success]:
    # check if the path is bypassing
    if not is_crossing(path):
        # increment the counter
        cnt_b += path2weight[with,failure][path]

# compute net gain in success rate stemming from shift to crossing paths under spatial structure
net_gain = (cnt_c - cnt_b) / M
```

---

[1] B. Ju, Q. Zhang, J. Ge, R. Wang, J. Sun, X. Ge, J. Yu, S. Shan, B. Zhou, S. Song, X. Tang, J. Yu, J. Lan, J. Yuan, H. Wang, J. Zhao, S. Zhang, Y. Wang, X. Shi, L. Liu, J. Zhao, X. Wang, Z. Zhang, and L. Zhang, Human neutralizing antibodies elicited by sars-cov-2 infection, Nature **584**, 115–119 (2020).

[2] Y. Cao, B. Su, X. Guo, W. Sun, Y. Deng, L. Bao, Q. Zhu, X. Zhang, Y. Zheng, C. Geng, X. Chai, R. He, X. Li, Q. Lv, H. Zhu, W. Deng, Y. Xu, Y. Wang, L. Qiao, Y. Tan, L. Song, G. Wang, X. Du, N. Gao, J. Liu, J. Xiao, X.-d. Su, Z. Du, Y. Feng, C. Qin, C. Qin, R. Jin, and X. S. Xie, Potent neutralizing antibodies against sars-cov-2 identified by high-throughput single-cell sequencing of convalescent patients' b cells, Cell **182**, 73 (2020).

[3] T. F. Rogers, F. Zhao, D. Huang, N. Beutler, A. Burns, W.-t. He, O. Limbo, C. Smith, G. Song, J. Woehl, L. Yang, R. K. Abbott, S. Callaghan, E. Garcia, J. Hurtado, M. Parren, L. Peng, S. Ramirez, J. Ricketts, M. J. Ricciardi, S. A. Rawlings, N. C. Wu, M. Yuan, D. M. Smith, D. Nemazee, J. R. Teijaro, J. E. Voss, I. A. Wilson, R. Andrabi, B. Briney, E. Landais, D. Sok, J. G. Jardine, and D. R. Burton, Isolation of potent sars-cov-2 neutralizing antibodies and protection from disease in a small animal model, Science **369**, 956–963 (2020).

[4] P. J. M. Brouwer, T. G. Caniels, K. van der Straten, J. L. Snitselaar, Y. Aldon, S. Bangaru, J. L. Torres, N. M. A. Okba, M. Claireaux, G. Kerster, A. E. H. Bentlage, M. M. van Haaren, D. Guerra, J. A. Burger, E. E. Schermer, K. D. Verheul, N. van der Velde, A. van der Kooi, J. van Schooten, M. J. van Breemen, T. P. L. Bijl, K. Sliepen, A. Aartse, R. Derking, I. Bontjer, N. A. Kootstra, W. J. Wiersinga, G. Vidarsson, B. L. Haagmans, A. B. Ward, G. J. de Bree, R. W. Sanders, and M. J. van Gils, Potent neutralizing antibodies from covid-19 patients define multiple targets of vulnerability, Science **369**, 643–650 (2020).

[5] Y. Wu, F. Wang, C. Shen, W. Peng, D. Li, C. Zhao, Z. Li, S. Li, Y. Bi, Y. Yang, Y. Gong, H. Xiao, Z. Fan, S. Tan, G. Wu, W. Tan, X. Lu, C. Fan, Q. Wang, Y. Liu, C. Zhang, J. Qi, G. F. Gao, F. Gao, and L. Liu, A noncompeting pair of human neutralizing antibodies block covid-19 virus binding to its receptor ace2, Science **368**, 1274–1278 (2020).

[6] E. Seydoux, L. J. Homad, A. J. MacCamy, K. R. Parks, N. K. Hurlburt, M. F. Jennewein, N. R. Akins, A. B. Stuart, Y.-H. Wan, J. Feng, R. E. Whaley, S. Singh, M. Boeckh, K. W. Cohen, M. J. McElrath, J. A. Englund, H. Y. Chu, M. Pancera, A. T. McGuire, and L. Stamatatos, Analysis of a sars-cov-2-infected individual reveals development of potent neutralizing antibodies with limited somatic mutation, Immunity **53**, 98 (2020).

[7] C. O. Barnes, A. P. West, K. E. Huey-Tubman, M. A. Hoffmann, N. G. Sharaf, P. R. Hoffman, N. Koranda, H. B. Gristick, C. Gaebler, F. Muecksch, J. C. C. Lorenzi, S. Finkin, T. Hägglöf, A. Hurley, K. G. Millard, Y. Weisblum, F. Schmidt, T. Hatziioannou, P. D. Bieniasz, M. Caskey, D. F. Robbiani, M. C. Nussenzweig, and P. J. Bjorkman, Structures of human antibodies bound to sars-cov-2 spike reveal common epitopes and recurrent features of antibodies, Cell **182**, 828 (2020).

[8] D. F. Robbiani, C. Gaebler, F. Muecksch, J. C. C. Lorenzi, Z. Wang, A. Cho, M. Agudelo, C. O. Barnes, A. Gazumyan, S. Finkin, T. Hägglöf, T. Y. Oliveira, C. Viant, A. Hurley, H.-H. Hoffmann, K. G. Millard, R. G. Kost, M. Cipolla, K. Gordon, F. Bianchini, S. T. Chen, V. Ramos, R. Patel, J. Dizon, I. Shimeliovich, P. Mendoza, H. Hartweger, L. Nogueira, M. Pack, J. Horowitz, F. Schmidt, Y. Weisblum, E. Michailidis, A. W. Ashbrook, E. Waltari, J. E. Pak, K. E. Huey-Tubman, N. Koranda, P. R. Hoffman, A. P. West, C. M. Rice, T. Hatziioannou, P. J. Bjorkman, P. D. Bieniasz, M. Caskey, and M. C. Nussenzweig, Convergent antibody responses to SARS-CoV-2 in convalescent individuals, Nature **584**, 437 (2020).

[9] X. Han, Y. Wang, S. Li, C. Hu, T. Li, C. Gu, K. Wang, M. Shen, J. Wang, J. Hu, R. Wu, S. Mu, F. Gong, Q. Chen, F. Gao, J. Huang, Y. Long, F. Luo, S. Song, S. Long, Y. Hao, L. Li, Y. Wu, W. Xu, X. Cai, Q. Gao, G. Zhang, C. He,

K. Deng, L. Du, Y. Nai, W. Wang, Y. Xie, D. Qu, A. Huang, N. Tang, and A. Jin, A rapid and efficient screening system for neutralizing antibodies and its application for sars-cov-2, Frontiers in Immunology **12**, 10.3389/fimmu.2021.653189 (2021).

[10] J. Kreye, S. M. Reincke, H.-C. Kornau, E. Sánchez-Sendin, V. M. Corman, H. Liu, M. Yuan, N. C. Wu, X. Zhu, C.-C. D. Lee, J. Trimpert, M. Höltje, K. Dietert, L. Stöffler, N. von Wardenburg, S. van Hoof, M. A. Homeyer, J. Hoffmann, A. Abdelgawad, A. D. Gruber, L. D. Bertzbach, D. Vladimirova, L. Y. Li, P. C. Barthel, K. Skriner, A. C. Hocke, S. Hippenstiel, M. Witzenrath, N. Suttorp, F. Kurth, C. Franke, M. Endres, D. Schmitz, L. M. Jeworowski, A. Richter, M. L. Schmidt, T. Schwarz, M. A. Müller, C. Drosten, D. Wendisch, L. E. Sander, N. Osterrieder, I. A. Wilson, and H. Prüss, A therapeutic non-self-reactive sars-cov-2 antibody protects from lung pathology in a covid-19 hamster model, Cell **183**, 1058 (2020).

[11] C. Kreer, M. Zehner, T. Weber, M. S. Ercanoglu, L. Gieselmann, C. Rohde, S. Halwe, M. Korenkov, P. Schommers, K. Vanshylla, V. Di Cristanziano, H. Janicki, R. Brinker, A. Ashurov, V. Krähling, A. Kupke, H. Cohen-Dvashi, M. Koch, J. M. Eckert, S. Lederer, N. Pfeifer, T. Wolf, M. J. Vehreschild, C. Wendtner, R. Diskin, H. Gruell, S. Becker, and F. Klein, Longitudinal isolation of potent near-germline sars-cov-2-neutralizing antibodies from covid-19 patients, Cell **182**, 843 (2020).

[12] S. J. Zost, P. Gilchuk, R. E. Chen, J. B. Case, J. X. Reidy, A. Trivette, R. S. Nargi, R. E. Sutton, N. Suryadevara, E. C. Chen, E. Binshtein, S. Shrihari, M. Ostrowski, H. Y. Chu, J. E. Didier, K. W. MacRenaris, T. Jones, S. Day, L. Myers, F. Eun-Hyung Lee, D. C. Nguyen, I. Sanz, D. R. Martinez, P. W. Rothlauf, L.-M. Bloyet, S. P. J. Whelan, R. S. Baric, L. B. Thackray, M. S. Diamond, R. H. Carnahan, and J. E. Crowe, Rapid isolation and profiling of a diverse panel of human monoclonal antibodies targeting the sars-cov-2 spike protein, Nature Medicine **26**, 1422–1427 (2020).

[13] L. Liu, P. Wang, M. S. Nair, J. Yu, M. Rapp, Q. Wang, Y. Luo, J. F.-W. Chan, V. Sahi, A. Figueroa, X. V. Guo, G. Cerutti, J. Bimela, J. Gorman, T. Zhou, Z. Chen, K.-Y. Yuen, P. D. Kwong, J. G. Sodroski, M. T. Yin, Z. Sheng, Y. Huang, L. Shapiro, and D. D. Ho, Potent neutralizing antibodies against multiple epitopes on sars-cov-2 spike, Nature **584**, 450–456 (2020).

[14] N. K. Hurlburt, E. Seydoux, Y.-H. Wan, V. V. Edara, A. B. Stuart, J. Feng, M. S. Suthar, A. T. McGuire, L. Stamatatos, and M. Pancera, Structural basis for potent neutralization of sars-cov-2 and role of antibody affinity maturation, Nature Communications **11**, 10.1038/s41467-020-19231-9 (2020).

[15] S. Du, Y. Cao, Q. Zhu, P. Yu, F. Qi, G. Wang, X. Du, L. Bao, W. Deng, H. Zhu, J. Liu, J. Nie, Y. Zheng, H. Liang, R. Liu, S. Gong, H. Xu, A. Yisimayi, Q. Lv, B. Wang, R. He, Y. Han, W. Zhao, Y. Bai, Y. Qu, X. Gao, C. Ji, Q. Wang, N. Gao, W. Huang, Y. Wang, X. S. Xie, X.-d. Su, J. Xiao, and C. Qin, Structurally resolved sars-cov-2 antibody shows high efficacy in severely infected hamsters and provides a potent cocktail pairing strategy, Cell **183**, 1013 (2020).

[16] X. Chi, R. Yan, J. Zhang, G. Zhang, Y. Zhang, M. Hao, Z. Zhang, P. Fan, Y. Dong, Y. Yang, Z. Chen, Y. Guo, J. Zhang, Y. Li, X. Song, Y. Chen, L. Xia, L. Fu, L. Hou, J. Xu, C. Yu, J. Li, Q. Zhou, and W. Chen, A neutralizing human antibody binds to the n-terminal domain of the spike protein of sars-cov-2, Science **369**, 650–655 (2020).

[17] R. Shi, C. Shan, X. Duan, Z. Chen, P. Liu, J. Song, T. Song, X. Bi, C. Han, L. Wu, G. Gao, X. Hu, Y. Zhang, Z. Tong, W. Huang, W. J. Liu, G. Wu, B. Zhang, L. Wang, J. Qi, H. Feng, F.-S. Wang, Q. Wang, G. F. Gao, Z. Yuan, and J. Yan, A human neutralizing antibody targets the receptor-binding site of sars-cov-2, Nature **584**, 120–124 (2020).

[18] J. Hansen, A. Baum, K. E. Pascal, V. Russo, S. Giordano, E. Wloga, B. O. Fulton, Y. Yan, K. Koon, K. Patel, K. M. Chung, A. Hermann, E. Ullman, J. Cruz, A. Rafique, T. Huang, J. Fairhurst, C. Libertiny, M. Malbec, W.-y. Lee, R. Welsh, G. Farr, S. Pennington, D. Deshpande, J. Cheng, A. Watty, P. Bouffard, R. Babb, N. Levenkova, C. Chen, B. Zhang, A. Romero Hernandez, K. Saotome, Y. Zhou, M. Franklin, S. Sivapalasingam, D. C. Lye, S. Weston, J. Logue, R. Haupt, M. Frieman, G. Chen, W. Olson, A. J. Murphy, N. Stahl, G. D. Yancopoulos, and C. A. Kyratsous, Studies in humanized mice and convalescent humans yield a sars-cov-2 antibody cocktail, Science **369**, 1010–1014 (2020).

[19] N. C. Wu, M. Yuan, H. Liu, C.-C. D. Lee, X. Zhu, S. Bangaru, J. L. Torres, T. G. Caniels, P. J. Brouwer, M. J. van Gils, R. W. Sanders, A. B. Ward, and I. A. Wilson, An alternative binding mode of IGHV3-53 antibodies to the SARS-CoV-2 receptor binding domain, Cell Reports **33**, 108274 (2020).

[20] T. J. C. Tan, M. Yuan, K. Kuzelka, G. C. Padron, J. R. Beal, X. Chen, Y. Wang, J. Rivera-Cardona, X. Zhu, B. M. Stadtmueller, C. B. Brooke, I. A. Wilson, and N. C. Wu, Sequence signatures of two public antibody clonotypes that bind SARS-CoV-2 receptor binding domain, Nature Communications **12**, 10.1038/s41467-021-24123-7 (2021).

[21] L. Benatuil, J. M. Perez, J. Belk, and C.-M. Hsieh, An improved yeast transformation method for the generation of very large human antibody libraries, Protein Engineering, Design and Selection **23**, 155 (2010).

[22] E. H. Kellogg, A. Leaver-Fay, and D. Baker, Role of conformational sampling in computing mutation-induced changes in protein structure and stability, Proteins: Structure, Function, and Bioinformatics **79**, 830–838 (2010).

[23] I. G. Szendro, M. F. Schenk, J. Franke, J. Krug, and J. A. G. M. de Visser, Quantitative analyses of empirical fitness landscapes, Journal of Statistical Mechanics: Theory and Experiment **2013**, P01005 (2013).

[24] J. Otwinowski, Biophysical inference of epistasis and the effects of mutations on protein stability and function, Molecular Biology and Evolution **35**, 2345 (2018).

[25] A. Tareen, M. Kooshkbaghi, A. Posfai, W. T. Ireland, D. M. McCandlish, and J. B. Kinney, MAVE-NN: learning genotype-phenotype maps from multiplex assays of variant effect, Genome Biology **23**, 10.1186/s13059-022-02661-7 (2022).

[26] S. A. Kauffman and E. D. Weinberger, The NK model of rugged fitness landscapes and its application to maturation of the immune response, Journal of Theoretical Biology **141**, 211 (1989).

[27] S. Wang and L. Dai, Evolving generalists in switching rugged landscapes, PLoS computational biology **15**, e1007320 (2019).

[28] L. Barnett, Ruggedness and neutrality-the nkp family of fitness landscapes, in Artificial Life VI: Proceedings of the sixth international conference on Artificial life (1998) pp. 18–27.

[29] M. E. J. Newman and R. Engelhardt, Effects of selective neutrality on the evolution of molecular species, Proceedings of

the Royal Society of London. Series B: Biological Sciences **265**, 1333 (1998).
[30] A. S. Perelson and C. A. Macken, Protein evolution on partially correlated landscapes., Proceedings of the National Academy of Sciences **92**, 9657 (1995).
[31] J. F. de Cossio-Diaz, G. Uguzzoni, and A. Pagnani, Unsupervised inference of protein fitness landscape from deep mutational scan, Molecular Biology and Evolution **38**, 318 (2020).
[32] A. Agarwala and D. S. Fisher, Adaptive walks on high-dimensional fitness landscapes and seascapes with distance-dependent statistics, Theoretical Population Biology **130**, 13 (2019).
[33] E. T. Jaynes, Information theory and statistical mechanics, Physical review **106**, 620 (1957).
[34] E. T. Jaynes, Information theory and statistical mechanics. ii, Physical review **108**, 171 (1957).
[35] T. Hinkley, J. Martins, C. Chappey, M. Haddad, E. Stawiski, J. M. Whitcomb, C. J. Petropoulos, and S. Bonhoeffer, A systems analysis of mutational effects in hiv-1 protease and reverse transcriptase, Nature genetics **43**, 487 (2011).
[36] M. Weigt, R. A. White, H. Szurmant, J. A. Hoch, and T. Hwa, Identification of direct residue contacts in protein–protein interaction by message passing, Proceedings of the National Academy of Sciences **106**, 67 (2009).
[37] F. Morcos, A. Pagnani, B. Lunt, A. Bertolino, D. S. Marks, C. Sander, R. Zecchina, J. N. Onuchic, T. Hwa, and M. Weigt, Direct-coupling analysis of residue coevolution captures native contacts across many protein families, Proceedings of the National Academy of Sciences **108**, E1293 (2011).
[38] A. M. Phillips, K. R. Lawrence, A. Moulana, T. Dupic, J. Chang, M. S. Johnson, I. Cvijovic, T. Mora, A. M. Walczak, and M. M. Desai, Binding affinity landscapes constrain the evolution of broadly neutralizing anti-influenza antibodies, eLife **10**, 10.7554/elife.71393 (2021).
[39] L. Ferretti, B. Schmiegelt, D. Weinreich, A. Yamauchi, Y. Kobayashi, F. Tajima, and G. Achaz, Measuring epistasis in fitness landscapes: The correlation of fitness effects of mutations, Journal of Theoretical Biology **396**, 132 (2016).
[40] C. Bank, S. Matuszewski, R. T. Hietpas, and J. D. Jensen, On the (un)predictability of a large intragenic fitness landscape, Proceedings of the National Academy of Sciences **113**, 14085 (2016).
[41] S.-C. Park and J. Krug, Clonal interference in large populations, Proceedings of the National Academy of Sciences **104**, 18135 (2007).
[42] A. Bergman, D. B. Goldstein, K. E. Holsinger, and M. W. Feldman, Population structure, fitness surfaces, and linkage in the shifting balance process, Genetical Research **66**, 85 (1995).
[43] J. R. Nahum, P. Godfrey-Smith, B. N. Harding, J. H. Marcus, J. Carlson-Stevermer, and B. Kerr, A tortoise–hare pattern seen in adapting structured and unstructured populations suggests a rugged fitness landscape in bacteria, Proceedings of the National Academy of Sciences **112**, 7530–7535 (2015).

sequence locus	structural role
G26	steric constraints
V27	steric constraints
T28	hydrogen bonding
S31	hydrogen bonding (considering rotamers of K458 and Q474)
S35	steric constraints
V50	steric constraints
S53	available hydroxyl group for hydrogen bonding with solvent; backbone constraints for hydrogen bonding between V_H Y52 and K417
S56	hydrogen bonding
T57	increase in solvent accessible surface area
F58	π stacking between the π atoms of the benzene ring and carbonyl backbone of T415

TABLE S1. Structural context of the 10 antibody sites of interest. See also Fig. S2.

name	sequence (5' to 3')
COV107-23 heavy chain	GAGGTGCAGCTGGTGGAGTCTGGAGGAGGCTTGATCCAGCCTGGGGGGTCCCTGAGACTCTCCTGTGCAGCCTCTGGGTTCAC
COV107_HC	CGTCAGTAGTAACTACATGAGCTGGGTCCGCCAGGCTCCAGGGAAGGGGCTGGAGTGGGTCTCAGTTATTTATAGCGGTGGTA
	GCACATTCTACGCAGACTCCGTGAAGGGCCGATTCACCATCTCCAGAGACAATTCGAAGAACACGCTGTATCTTCAAATGAAC
	AGCCTGAGAGCCGAGGACACGGCCGTGTATTACTGTGCGAGAGACCTCGGAACGGGGTTATTCGACTACTGGGGCCAGGGAAC
	CCTGGTCACCGTCTCCTCAGCCTCTACCAAGGGACCAAGCGTGTTTCCACTGGCCCCCTCCTCTAAGTCCACCTCTGGAGGCA
	CAGCCGCCCTGGGCTGTCTGGTGAAGGACTACTTCCCCGAGCCTGTGACCGTGTCTTGGAACAGCGGCGCCCTGACCTCCGGA
	GTGCACACATTTCCCGCCGTGCTCCAGAGCAGCGGACTGTACAGCCTGTCTAGCGTGGTGACCGTGCCTTCCTCTAGCCTGGG
	CACCCAGACATATATCTGCAACGTGAATCACAAGCCATCCAATACAAAGGTGGATAAGAAGGTGGAGCCCAAGTCTTGT
COV107-23 kappa chain	GACATCCAGTTGACCCAGTCTCCATCCTTCCTGTCTGCATCTGTAGGAGACAGAGTCACCATCACTTGCCGGGCCAGTCAGGG
COV107_KC	CATTAGCAGTTATTTAGCCTGGTATCAGCAAAAACCAGGGAAAGCCCCTAAGCTCCTGATCTATGCTGCATCCACTTTGCAAA
	GTGGGGTCCCATCAAGGTTCAGCGGCAGTGGATCTGGGACAGAATTCACTCTCACAATCAGCAGCCTGCAGCCTGAAGATTTT
	GCAACTTATTACTGTCAACAGCTTGATAGTTACCCTCCGGGCACTTTCGGCCCTGGGACCAAAGTGGATATCAAAAGGACCGT
	GGCAGCACCTTCCGTGTTCATCTTTCCCCCTTCTGACGAGCAGCTGAAGTCTGGCACAGCCAGCGTGGTGTGCCTGCTGAACA
	ACTTCTACCCAAGAGAGGCCAAGGTGCAGTGGAAGGTGGATAACGCCCTCCAGTCTGGCAATAGCCAGGAGTCCGTGACCGAG
	CAGGACTCTAAGGATAGCACATATTCCCTGTCTAGCACCCTGACACTGAGCAAGGCCGACTACGAGAAGCACAAGGTGTATGC
	ATGCGAGGTGACCCACCAGGGACTGTCCTCTCCCGTGACAAAGAGCTTCAACAGAGGCGAGTGTTCC

TABLE S2. Nucleotide sequences of COV107-23 heavy and kappa chains used as template to generate combinatorially complete antibody variants.

primer name	sequence (5' to 3')
COV107_HC_F	TGT ACT TGC CGC CGC TCA ACC AGC AGA GGT GCA GCT GGT GGA GTC TGG
COV107_HC_R	CAG GCC CCC GAG GCC ACA AGA CTT GGG CTC CAC CTT CT
ERBV_F	GGC GGA AAG CCA ATC CCC AAT CCA C
ERBV_R	TGC TGG TTG AGC GGC GGC AAG TAC A
COV107_KC_F	GCG GCC CAG CCG GCC GAC ATC CAG TTG ACC CAG TCT CC
COV107_KC_2R	GTA GTG GAT TGG GGA TTG GCT TTC CGC CGG AAC ACT CGC CTC TGT TGA AGC TCT TTG TCA
COV107_SHMlib_VF	AGA CTC CGT GAA GGG CCG ATT CAC C
COV107_SHMlib_VR	CTG CAC AGG AGA GTC TCA GGG ACC C
COV107_SHMlib_F1	GGG TTC ACC GTC AGT AGT AAC TAC ATG AGC TGG GTC CGC CAG GCT CCA GG
COV107_SHMlib_R1	GTA GWA TGY GST ACC ACC GGR ATA AAT AAS TGA GAC CCA CTC CAG CCC CT
COV107_SHMlib_F2	GGG TCC CTG AGA CTC TCC TGT GCA GCC TCT GRG NTC AYC GTC AGT AGW AAC TAC A
COV107_SHMlib_R2	GGT GAA TCG GCC CTT CAC GGA GTC TGC GTA GWA TGY GST ACC ACC GGR ATA AAT
COV107_SHMlib_F3	TGG AGG AGG CTT GAT CCA GCC TGG GGG GTC CCT GAG ACT CTC CTG TGC AG
COV107_SHMlib_R3	TGT TCT TCG AAT TGT CTC TGG AGA TGG TGA ATC GGC CCT TCA CGG AGT CT
COV107_SHMlib_recoverF	CAC TCT TTC CCT ACA CGA CGC TCT TCC GAT CTT CCC TGA GAC TCT CCT GTG CAG CC
COV107_SHMlib_recoverR	GAC TGG AGT TCA GAC GTG TGC TCT TCC GAT CTG AAT CGG CCC TTC ACG GAG TCT GC

TABLE S3. List of primers used in this study.

rank	sequence $\mathbf{s}$										fitness $F(\mathbf{s})$
	26	27	28	31	35	50	53	56	57	58	
1	1	1	1	1	1	0	1	1	0	1	1.516
2	1	0	1	1	1	0	1	0	1	0	1.462
3	0	1	0	1	1	0	1	0	0	1	1.447
4	1	1	1	0	1	0	1	1	0	0	1.379
5	1	1	0	0	1	0	1	0	0	0	1.378
6	1	1	1	1	0	1	1	0	1	1	1.305
7	1	1	1	1	0	0	0	0	0	1	1.286

TABLE S4. Binary sequence identity of local fitness maxima ranked by their fitness values $F(\mathbf{s})$. In this binary representation, 0 and 1 respectively indicate the absence and presence of a mutation at the corresponding site (labelled at the top). Fitness values are determined from the specific epistasis model (main Eq. 1) trained on combinatorial mutagenesis data through unsupervised learning and cross-validation.

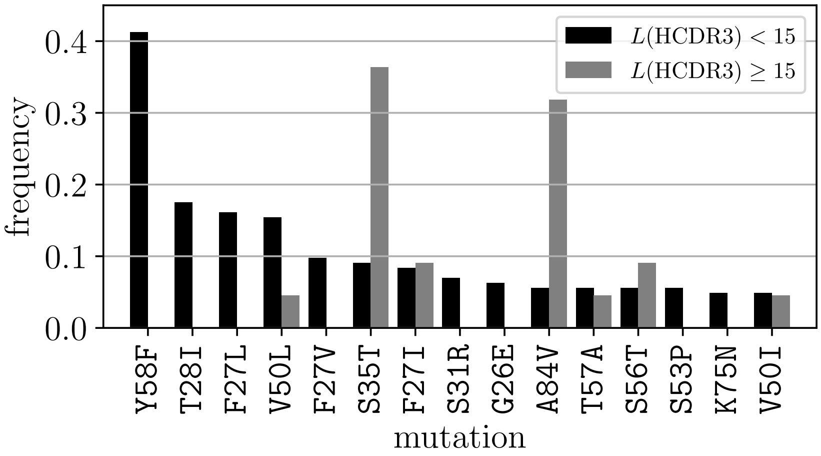

FIG. S1. **Frequencies of commonly found somatic hypermutations among IGHV3-53/3-66-encoded antibodies.** Common somatic hypermutations in IGHV 3-53/3-66-encoded antibodies against SARS-CoV-2 spike receptor binding domain (RBD). Antibody sequences are sorted by the length of their HCDR3 region, L(HCDR3), as defined through Kabat numbering. The listed mutations occur in the HCDR1 or HCDR2 region.

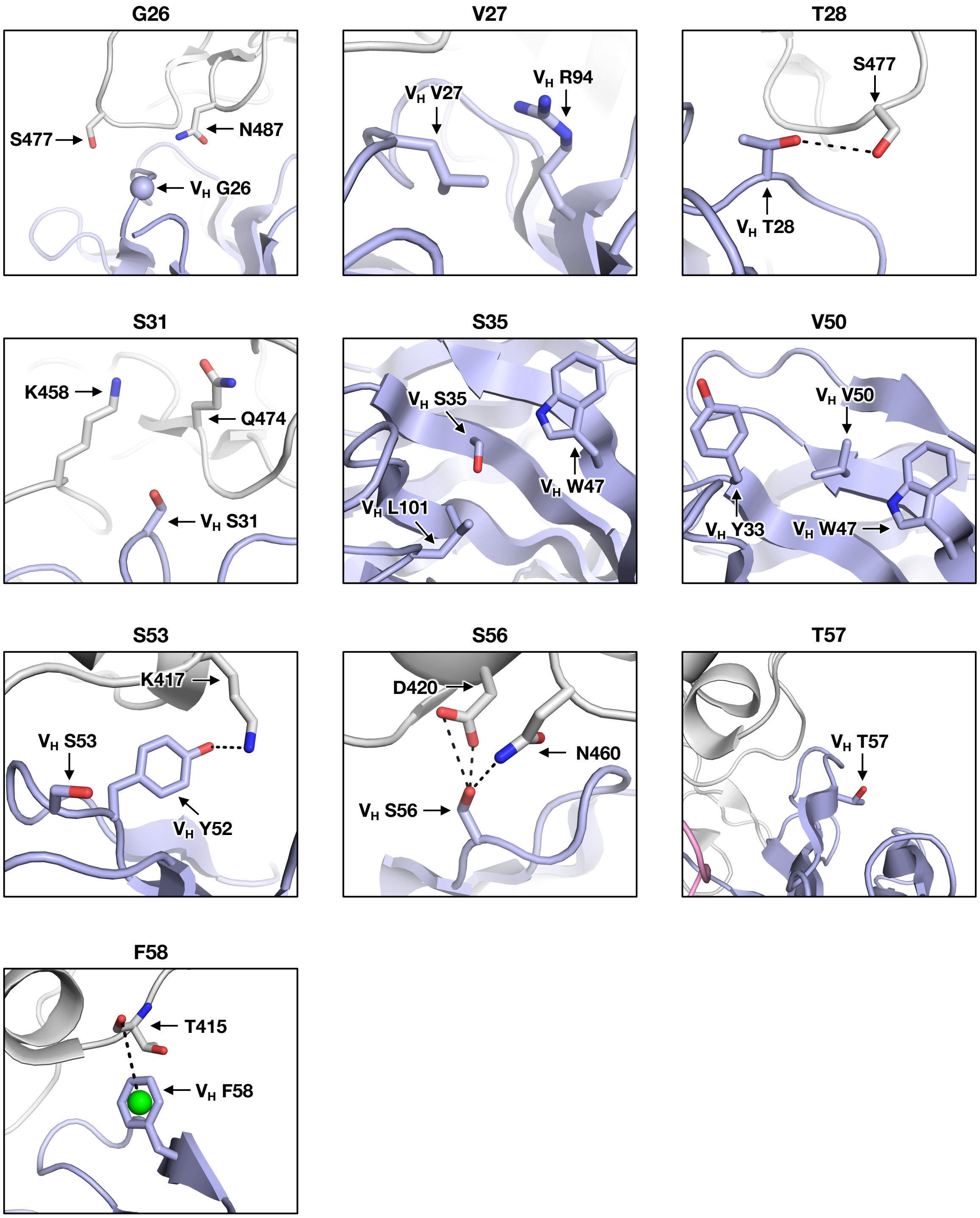

FIG. S2. **Structural context of the 10 antibody sites of interest.** Shown are the 10 sites and their interactions within the antibody (in blue), and with the spike receptor-binding domain (in grey). See also Table S1.

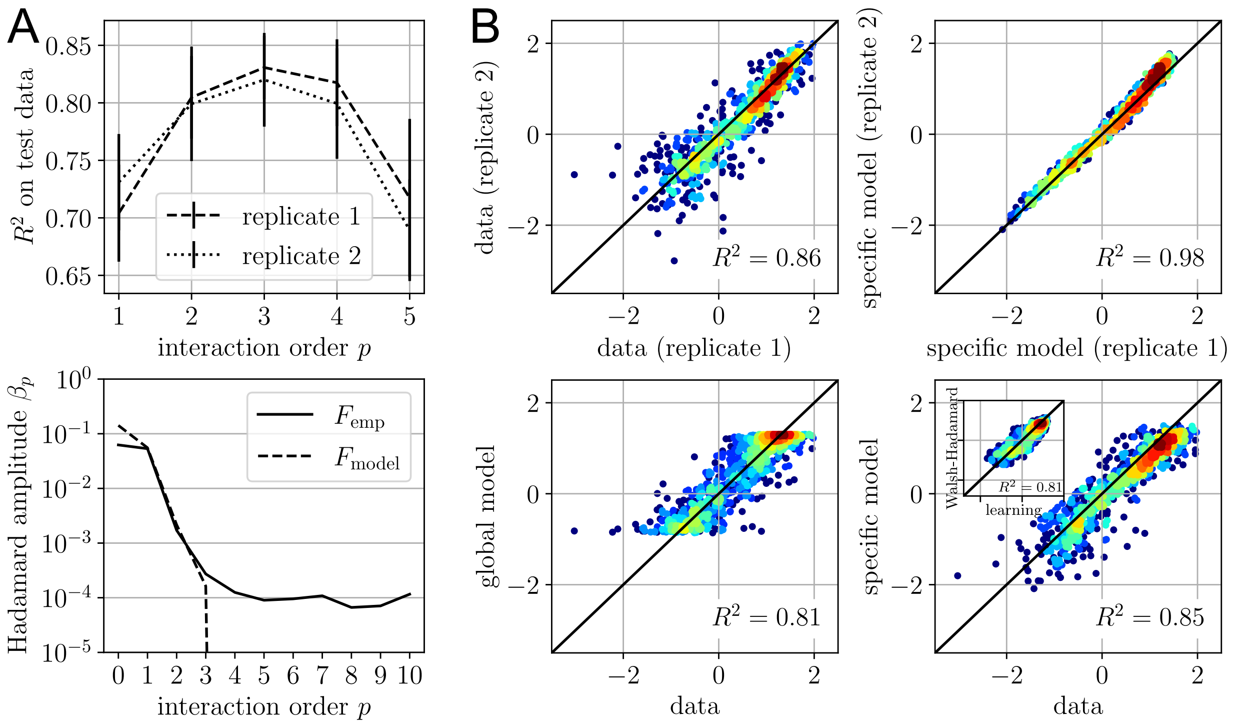

FIG. S3. **Inference of fitness landscape models.** **(A)** (Top) Cross-validation of the specific epistasis model: Coefficient of determination R^2 between predicted fitness values $F(\mathbf{s})$ and empirical values $F_{\text{emp}}(\mathbf{s})$ for genotypes excluded from model training using unsupervised learning. R^2 peaks at the third order ($p = 3$) for both experimental replicates. (Bottom) Amplitude β_p of Hadamard coefficients at all interaction orders p for empirical fitness values $F_{\text{emp}}(\mathbf{s})$ and up to the third order for specific epistasis model $F_{\text{model}}(\mathbf{s})$ (from Fig. 1C). **(B)** Correlation between empirical fitness values $F_{\text{emp}}(\mathbf{s})$ from two independent replicates of the combinatorial mutagenesis experiment (upper left), between inferred fitness values from the specific epistasis model using two replicate datasets (from Fig. 1C; upper right), between $F_{\text{emp}}(\mathbf{s})$ and the global epistasis model (from Fig. 1B; lower left) and between $F_{\text{emp}}(\mathbf{s})$ and the specific epistasis model inferred by unsupervised learning on 2^L sequences (from Fig. 1C; lower right); the latter two use data from replicate 1 of the combinatorial mutagenesis experiment. Inset of the lower right panel shows the correlation between fitness values from specific epistasis models obtained by unsupervised learning and by Walsh-Hadamard transform.

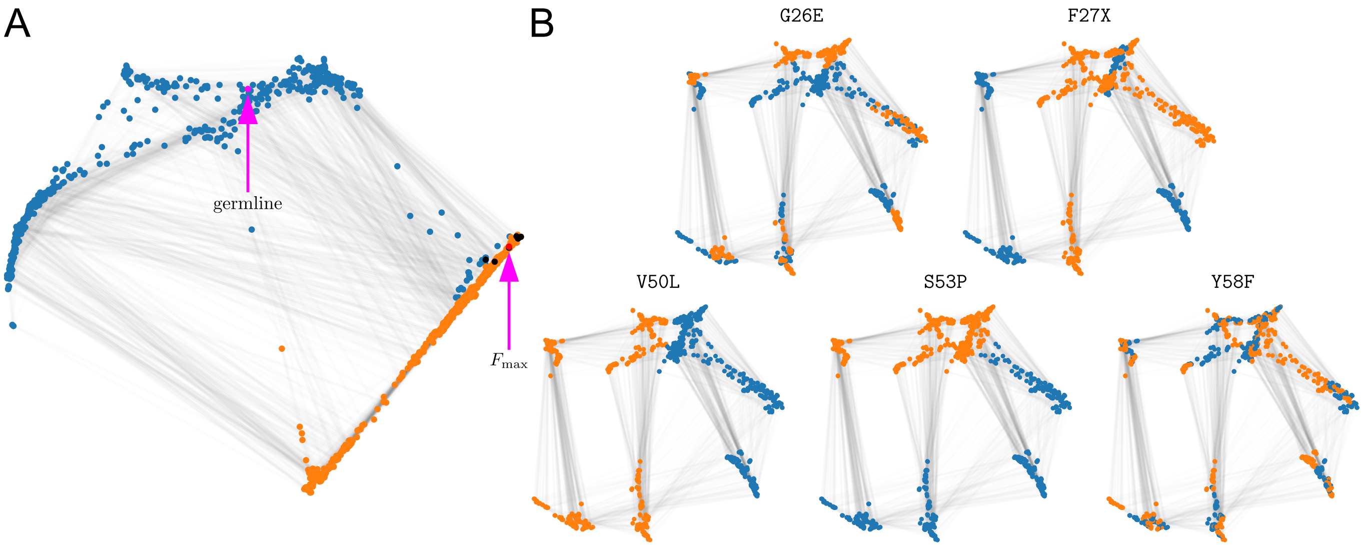

FIG. S4. **Two-dimensional embedding of high-dimensional antibody fitness landscape. (A)** Modified force-directed graph layout, which includes forces between next-nearest mutational neighbors ($d_H \leq 2$ instead of $d_H \leq 1$). Sequences are colored according to the state of the epistatic hotspot, blue for wild-type and orange for mutated. Additional constraints cause cluster coalescence (compared to Fig. 1D). **(B)** The same force-directed layout as in Fig. 1D, with sequences colored according to the state of the indicated site (blue for wild-type, orange for mutated). The mutational state of site 53 and site 50 most clearly captures the fitness variance, reflecting their strong additive effect that respectively increases and decreases fitness.

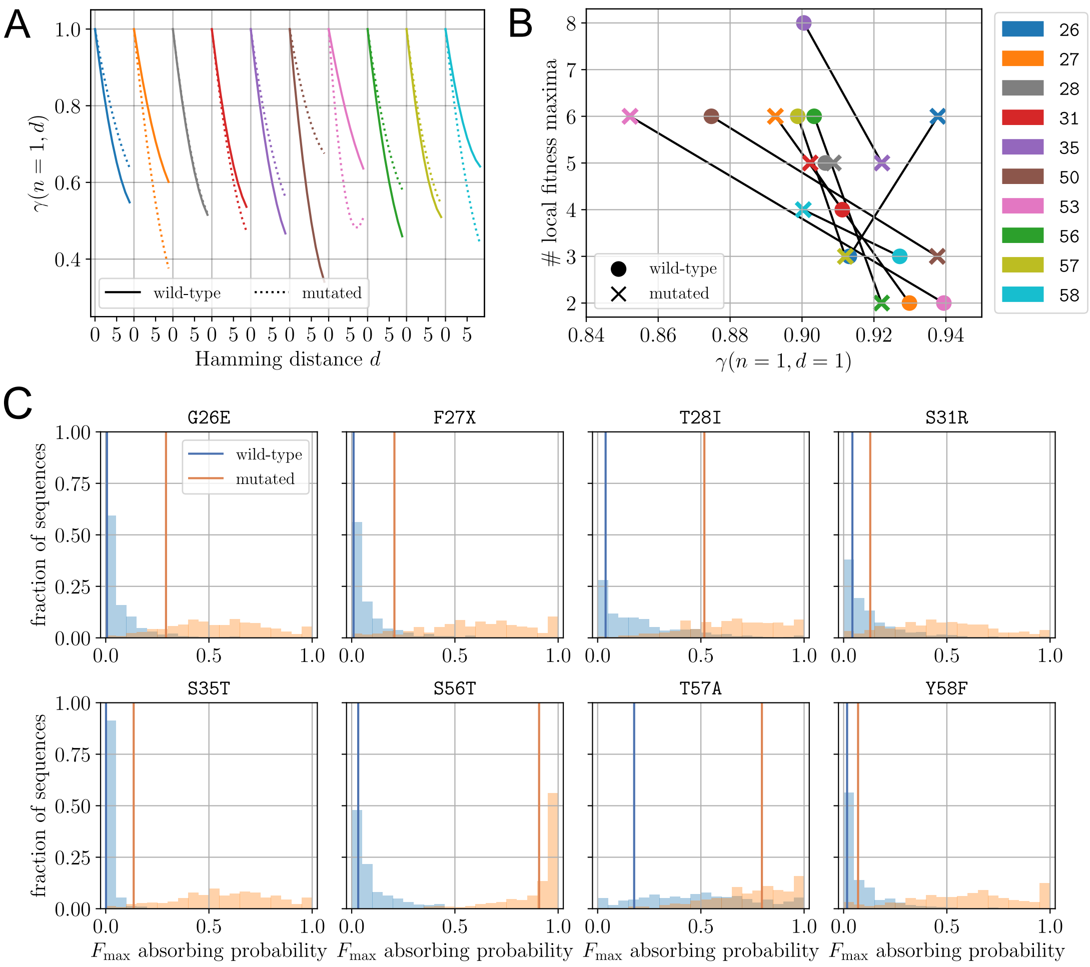

FIG. S5. **Landscape ruggedness and $F_{\max}$ accessibility. (A)** Ruggedness measure $\gamma(n=1,d)$ as a function of Hamming distance d in 9-dimensional sublandscapes when the pinned site is in its wild-type (solid line) or mutated (dashed line) state. Except for site 53 (magenta), ruggedness monotonously increases with d, as the correlation of fitness effects of mutations (γ value) decays with an increasing number of bit flips. **(B)** Change in ruggedness of 9-site sublandscapes due to mutation at the color-coded pinned site. A larger number of local fitness maxima correlates with a lower value of $\gamma(n=1,d=1)$, both indicating an increased ruggedness. Site 26 is an exception, whose mutation yields a higher γ value despite a larger number of fitness optima (i.e. a positive slope). **(C)** Accessibility (absorbing probability) of the global fitness optimum $F_{\max}$ in 9-site sublandscapes, the same as Fig. 3B, for the remaining 8 choices of the pinned site. In all 8 cases, mutation of the pinned site increases accessibility of $F_{\max}$; mutating sites 27, 31, and 58 yields more rugged sublandscapes, resulting in simultaneously stronger ruggedness and higher accessibility, like mutating the hotspot (site 53).

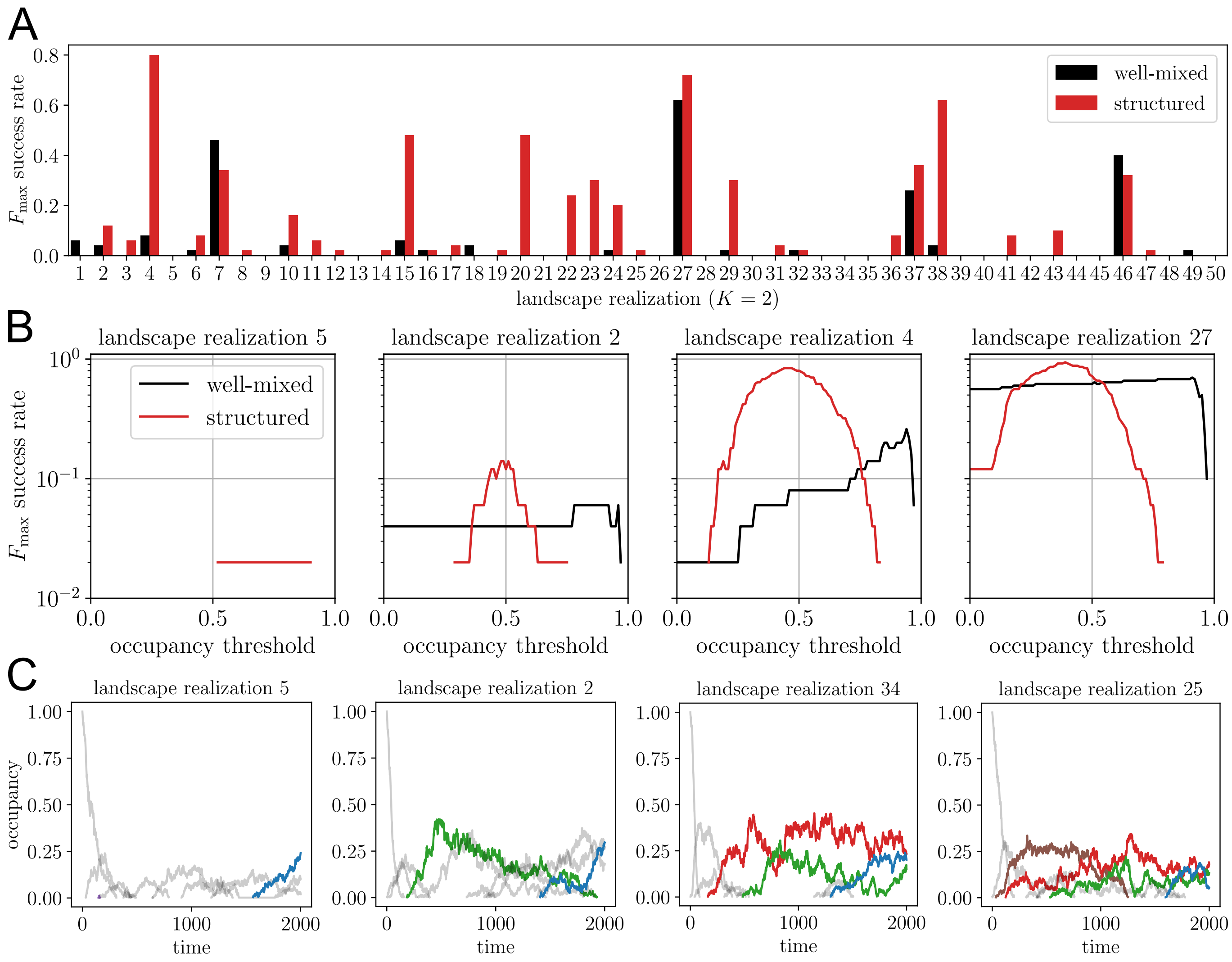

FIG. S6. **Lower success in NK landscapes of comparable ruggedness to the antibody landscape. (A)** Success rate of enriching $F_{\max}$ (above an occupancy threshold at 0.5) across 50 independent realizations of NK landscape with $K = 2$ in well-mixed and structured populations. Landscape realizations are constrained to exhibit the same number of fitness optima and the same mutational distance between the germline and $F_{\max}$ as the specific epistasis model. Overall, spatial structure (a limited spatial scale of competition) promotes success. **(B)** Success rate versus occupancy threshold for four example NK landscape realizations from (A) with vastly different levels of success. **(C)** Failure modes in constrained NK landscapes. Representative occupancy trajectories of the global fitness maximum (blue), local optima (non-blue colors) and non-peak genotypes (grey) along paths that fail to enrich $F_{\max}$ to the threshold at 0.5. From left to right, an increasing number of local optima are occupied to a considerable level before the global optimum receives a population influx.

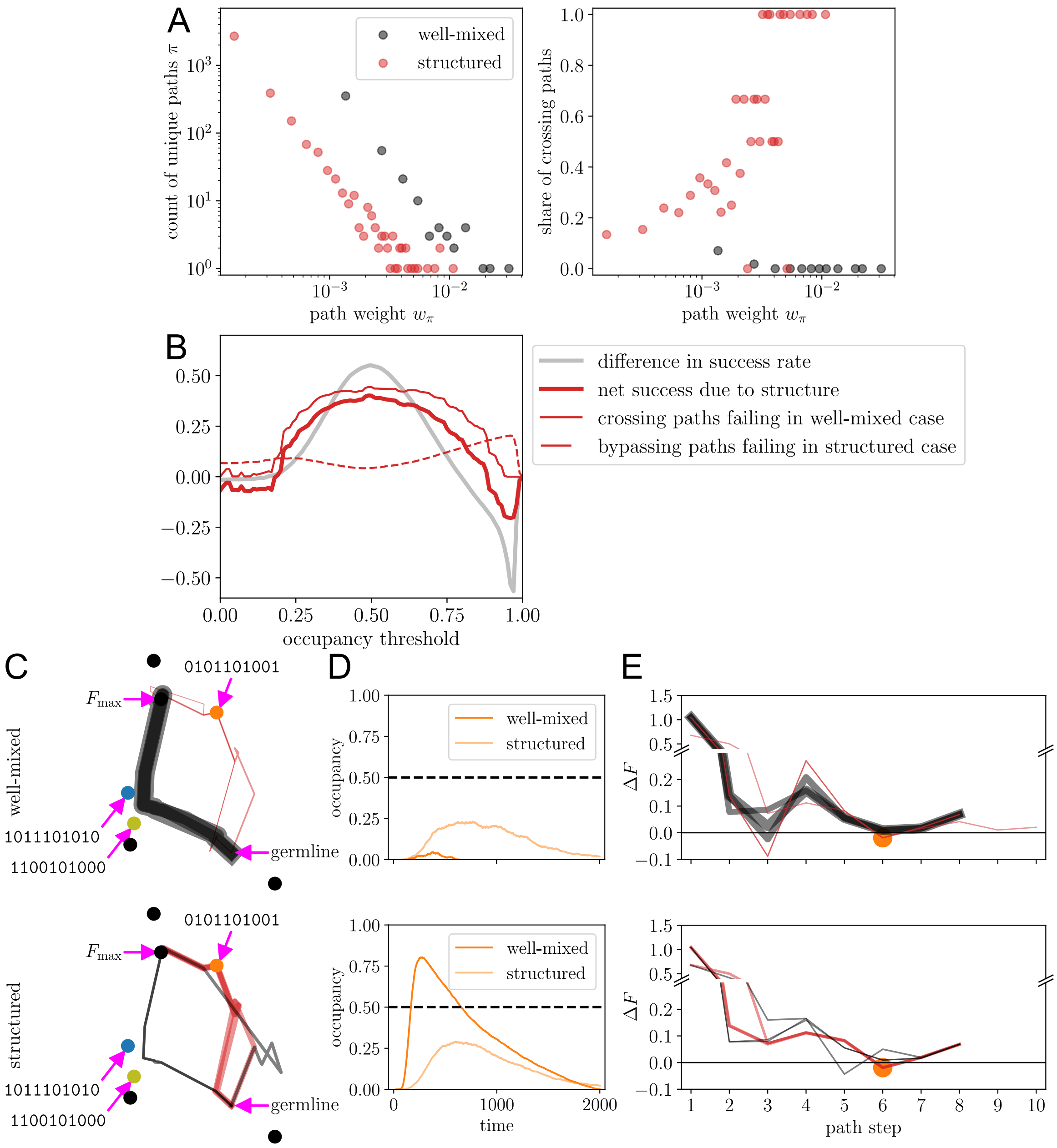

FIG. S7. **Spatial structure boosts $F_{\max}$ evolvability by permitting transient occupancy of a stepping-stone local optimum. (A)** (Left) Path weight spectrum (at an occupancy threshold 0.5) shows how many successful unique paths π carry a certain weight w_π among replicate populations. While well-mixed populations are dominated by a few high-weight paths, spatial structure opens many low-weight paths. (Right) Fraction of successful paths that cross a stepping-stone local optimum at each given path weight. With spatial structure, paths of high weight change from road-block bypassing paths to stepping-stone crossing paths (also see panel C). **(B)** Difference in success rate between structured and well-mixed conditions as a function of occupancy threshold (grey curve; calculated from Fig. 6A). Net success due to structure (thick red) can be explained to a large extent by subtracting the modest contribution of road-block bypassing paths failing in structured populations (dashed red) from the dominant contribution of stepping-stone crossing paths failing in well-mixed populations (thin red). **(C)** Top-three stepping-stone crossing paths (red) and top-three road-block bypassing paths (black) shown in t-SNE layout for successful populations at occupancy threshold 0.5. Line width is proportional to path weight. The orange dot marks the local fitness optimum acting as a stepping stone for top direct paths in structured populations. **(D)** Occupancy of the stepping stone (orange dot in C) over time along the top path in well-mixed and structured populations, averaged over all populations (success or failure) taking that path. Note the asymmetry: the top path without structure (bypassing) is likely to remain viable with structure (upper panel, lighter orange curve staying below the occupancy threshold), whereas the top path with structure (crossing) is highly prone to failure without structure (lower panel, darker orange curve rising above the occupancy threshold). **(E)** Fitness effect of successive mutations along the paths shown in C. The orange dot marks the stepping-stone genotype.

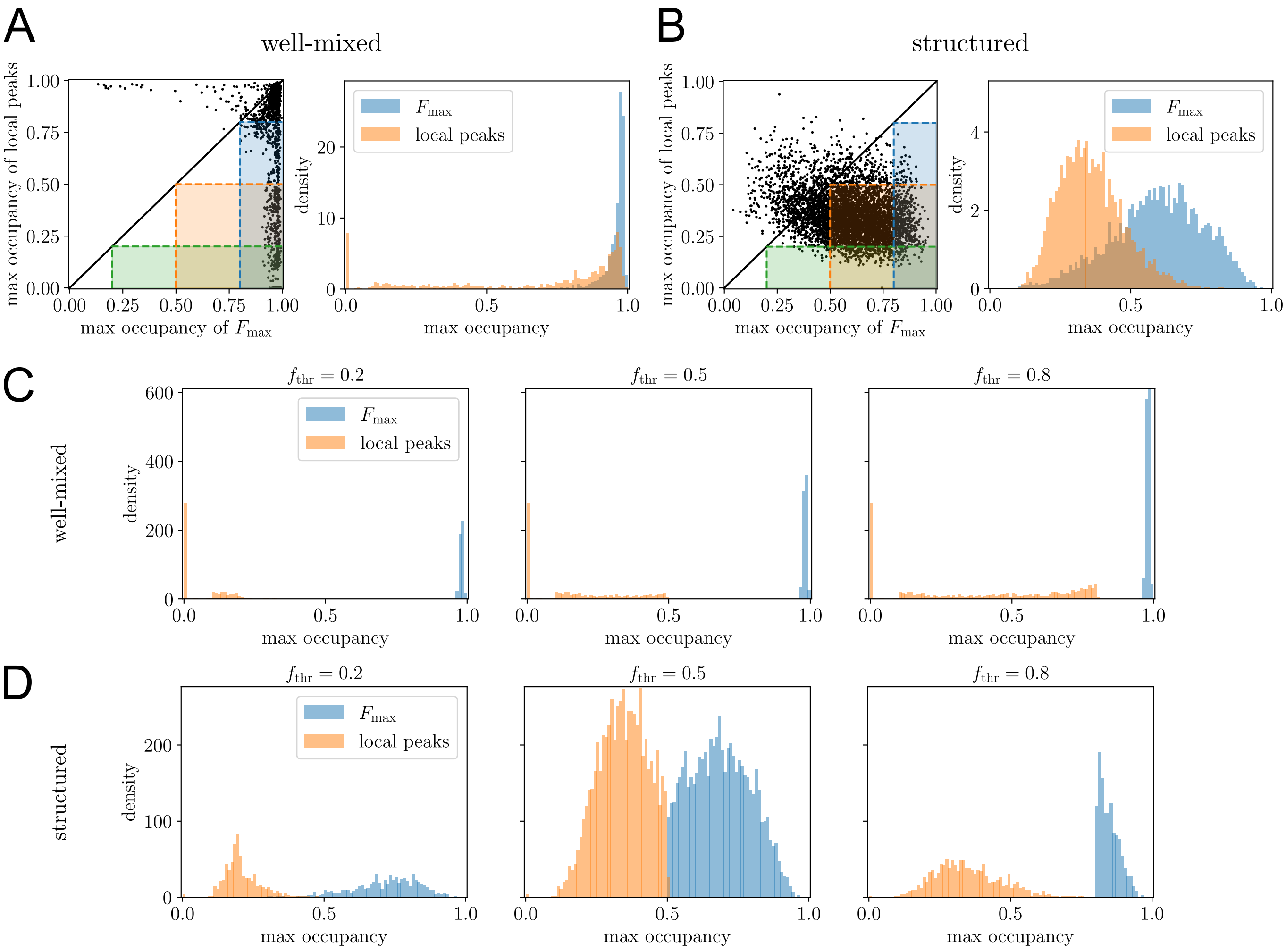

FIG. S8. **Understanding the boost of success by spatial structure via maximum occupancy of fitness optima.** **(A)** (Left) Maximum occupancy of the global optimum $F_{\max}$ versus maximum occupancy among suboptimal peaks, along unique paths in well-mixed populations, averaged over all populations (success or not) taking that path. Each dot represents a unique path. Colored rectangles delineate the window of success at different occupancy thresholds, $f_{\text{thr}} = 0.2$ (green), 0.5 (orange), and 0.8 (blue), respectively. (Right) Marginal distributions of maximum occupancy of $F_{\max}$ (blue) and suboptimal peaks (orange) obtained from projecting the scatter plot on the left to the horizontal and vertical axes, respectively. **(B)** Same as (A), for spatially structured populations. **(C)** Marginal distributions of maximum occupancy of $F_{\max}$ (blue) and suboptimal peaks (orange) for successful well-mixed populations (instead of paths). Zero maximum occupancy means that not a single suboptimal peak is ever visited by any population; a finite density is seen under well-mixed conditions (orange histograms). The area under the histogram (equal between blue and orange) corresponds to the success rate (shown in Fig. 6A for $f_{\text{thr}} = 0.5$). **(D)** Same as (C), for spatially structured populations.

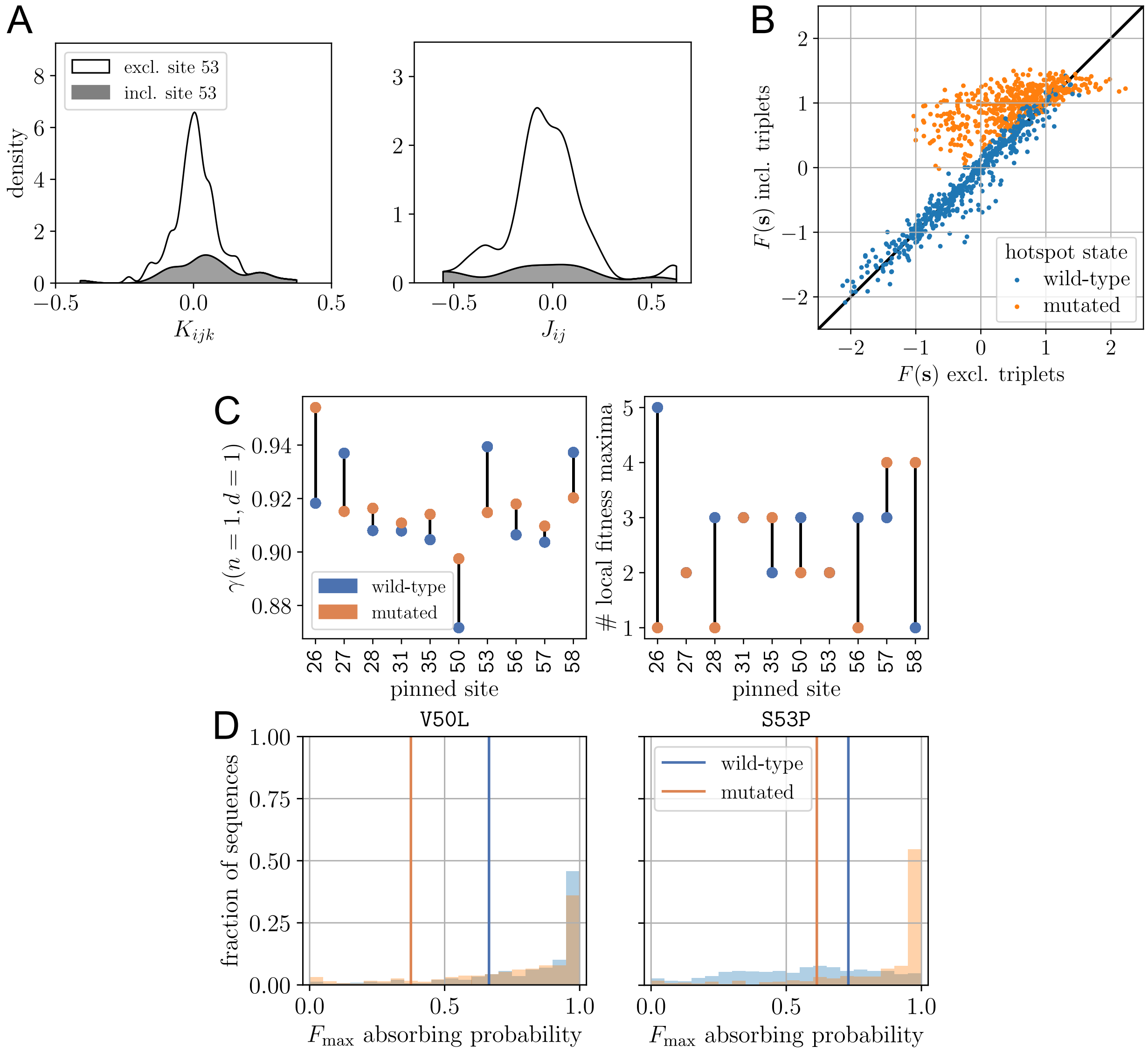

FIG. S9. **Higher-order epistasis is required for the simultaneous increase in ruggedness and accessibility upon hotspot mutation.** **(A)** Distribution of the third-order coefficients K_{ijk} (left) and that of the pairwise coefficients J_{ij} (right) from the inferred specific epistasis model described by Eq. (2). The shaded portion corresponds to when the hotspot is included among the triplet or pair of sites. Hotspot-associated triplet interactions can make strong positive fitness contributions. **(B)** Scatter plot of the inferred fitness values $F(\mathbf{s})$ of individual genotypes $\mathbf{s}$ when the triplet terms are included (y-axis) vs. excluded (x-axis). Blue/orange: the hotspot is wild-type/mutated. Note that fitness values of the second-order and third-order models are correlated when the hotspot is wild-type. Such correlation is lost when the hotspot is mutated, as this mutation turns on considerable fitness gain from triplet interactions — consistent with (A). **(C, D)** Ruggedness and $F_{\max}$ accessibility when third-order epistasis is excluded from the inferred model; to be compared with Figs. 3A-B where third-order terms are included. **(C)** Ruggedness of 9-site sublandscapes. Compared to Fig. 3A, without higher-order epistasis, hotspot mutation has very modest influence on ruggedness level (γ value) without changing the number of fitness peaks. **(D)** $F_{\max}$ accessibility measured by the absorbing probabilities under Monte-Carlo evolutionary dynamics. Vertical lines indicate starting from the antibody germline. Compared to Fig. 3B, without higher-order epistasis, hotspot mutation now reduces, rather than enhance, $F_{\max}$ accessibility starting from the germline (blue to orange line).

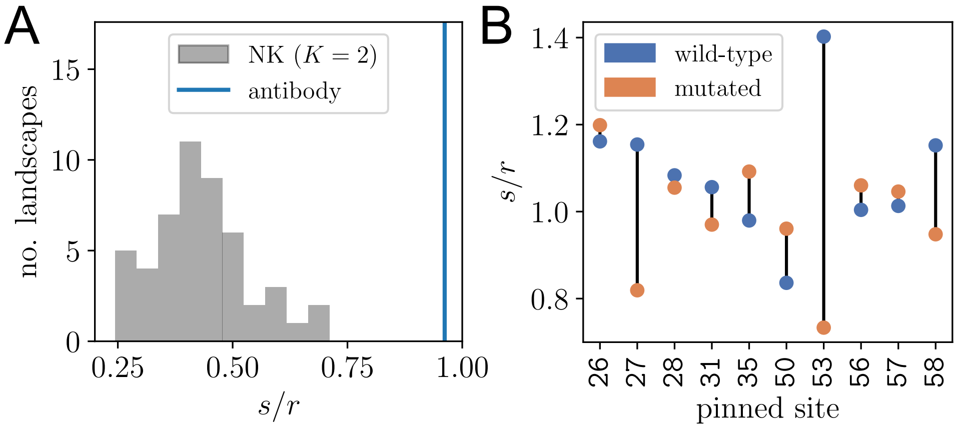

FIG. S10. **An inverted funnel-like shape of the antibody stability landscape. (A)** Slope-to-roughness ratio s/r, a measure of the global degree of smoothness, is higher for the antibody landscape (blue line) compared to those of constrained NK landscapes (gray histogram) that have the same number of fitness peaks as the antibody landscape, as well as the same distance between the wild-type genotype and the global fitness optimum. **(B)** Slope-to-roughness ratio s/r of 9-site sublandscapes with the indicated site being pinned in the wild-type state (blue) vs. the mutated state (orange). Mutation of the hotspot (site 53) induces the most significant change in s/r among 10 sites.

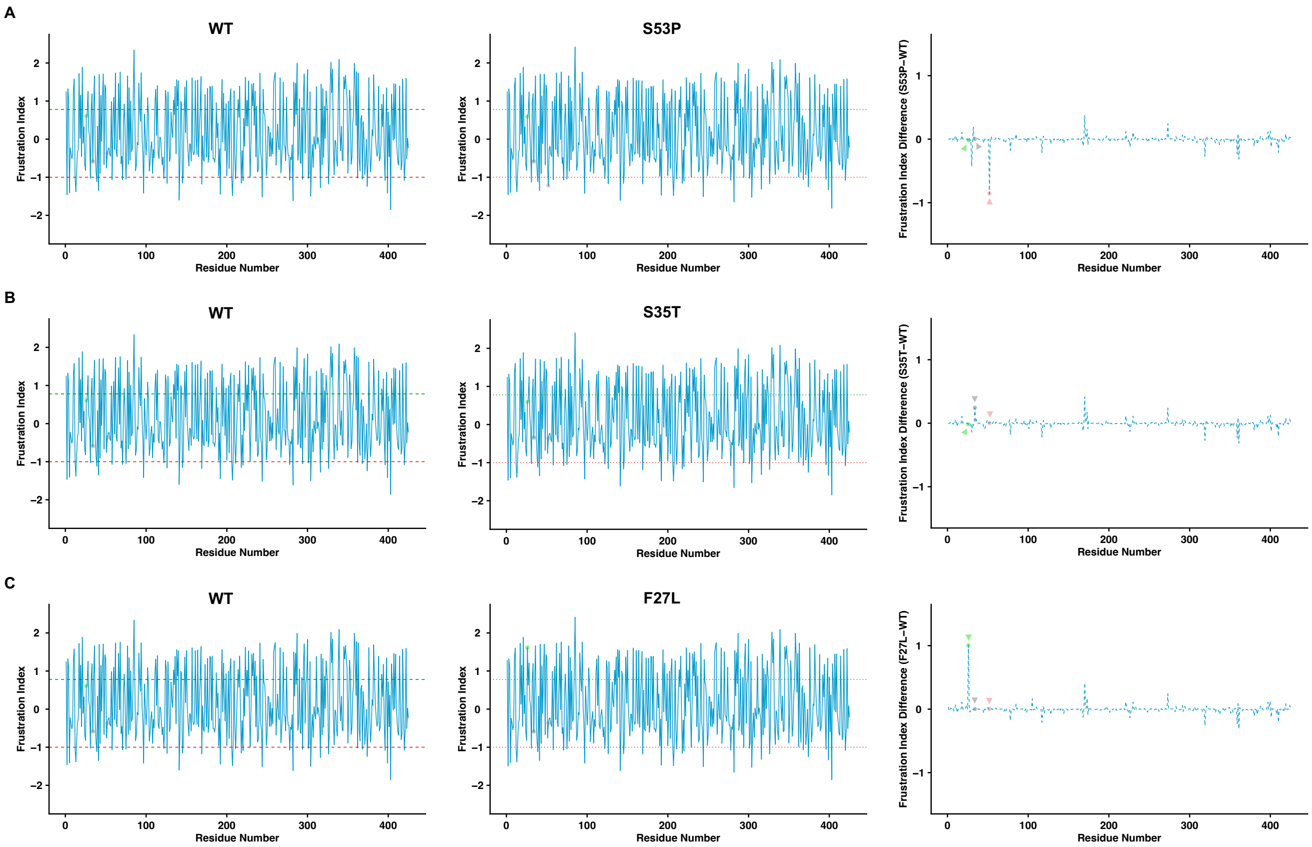

FIG. S11. **Single-residue frustration varies among sites of different epistatic importance.** Single-residue frustration indices (left two columns) and differences (right column) for fixed backbone of wild-type COV107-23, and **(A)** S53P mutant, **(B)** S35T mutant, and **(C)** F27L mutant. Green, grey, and pink arrows in right panels point to sites 27, 35, and 53, respectively. Green and red horizontal lines at frustration indices of 0.78 and −1 indicate the cutoff for a minimally frustrated residue and a highly frustrated residue, respectively.

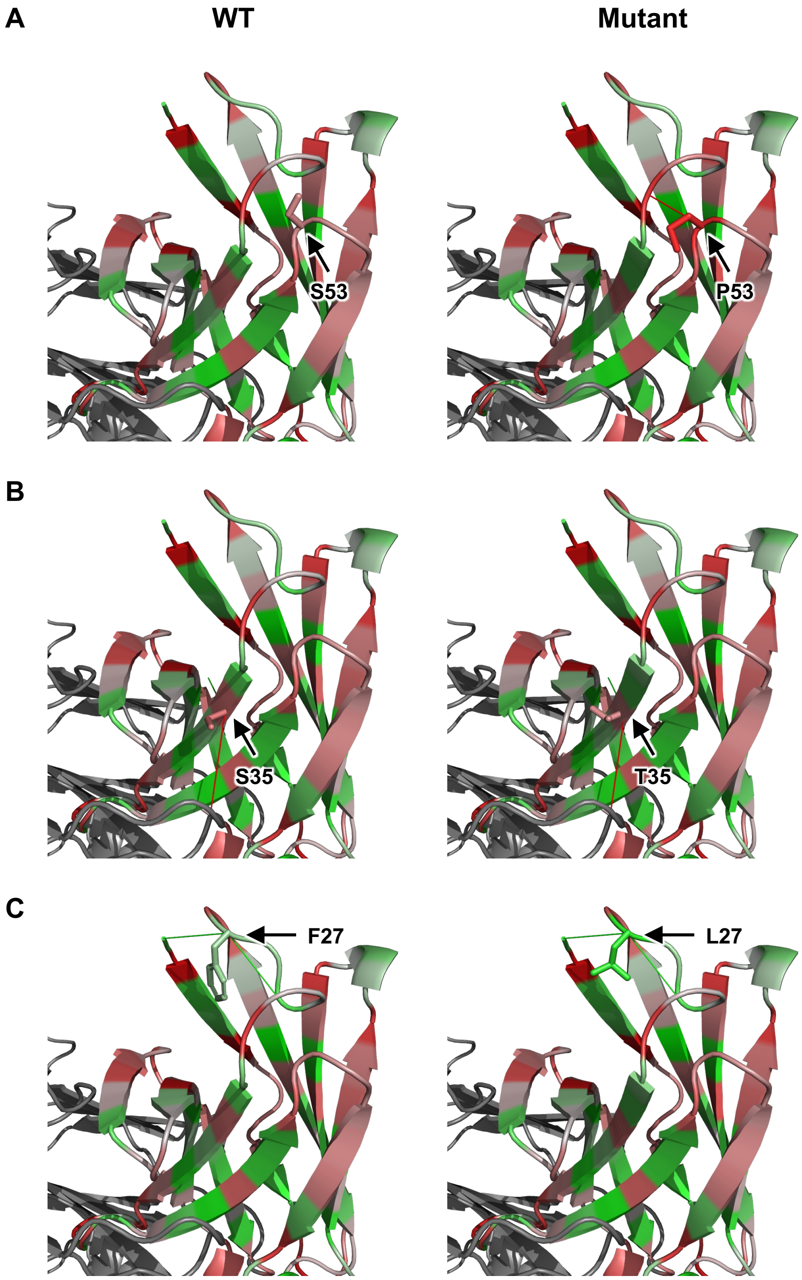

FIG. S12. **Contact frustration.** Frustration analysis of contacts of three sites for wild-type or mutant COV107-23 with **(A)** P53, **(B)** T35, or **(C)** L27 point mutation. Protein backbone is colored according to frustration index. Green and red backbone segments indicate minimally frustrated residues (index > 0.78) and highly frustrated residues (index < -1), respectively, according to single-residue frustration analysis. Green and red contacts indicate minimally frustrated (Z score > 0.78) and highly frustrated (Z score < -1) interactions, respectively.

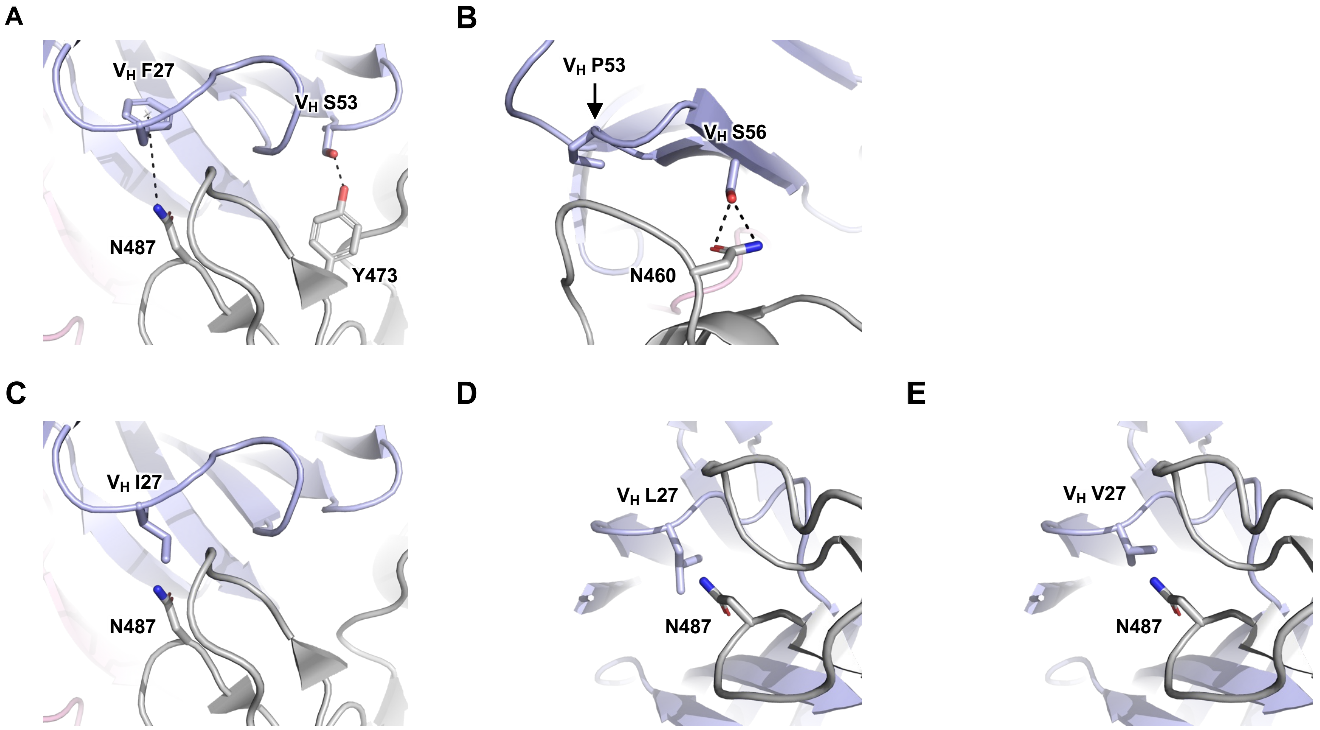

FIG. S13. **Structural interpretation of frustration.** Interactions between heavy chain of COV107-23 and spike protein receptor-binding domain (RBD). The crystal structure of wild-type or mutant COV107-23 was aligned with that of CC12.1 (PDB: `6XC2`) to determine interactions between antibody and spike protein. Both antibodies use the same heavy and light chain variable genes. All structures used are from fixed-backbone mutagenesis. **(A)** N487 forms a cation-pi interaction with F27 of antibody heavy chain. Y473 forms a hydrogen bond with S53 of antibody heavy chain. **(B)** P53 can rigidify the flexible HCDR2 loop so that S56 of the heavy chain can stably form hydrogen bonds with N460 of spike RBD. **(C-E)** Van der Waals interactions between **(C)** I27, **(D)** L27, or **(E)** V27 of COV107-23 and N487 of spike RBD. Grey: spike protein receptor-binding domain; blue: heavy chain; pink: light chain.
